# Strategies for knowledge translation of a palliative approach outside specialized palliative care services: a scoping review

**DOI:** 10.1186/s12904-022-00929-0

**Published:** 2022-03-22

**Authors:** Joakim Öhlén, Susanna Böling, Hanan HamdanAlshehri, Margareta Brännström, Ingela Henoch, Eva Hessman, Stefan Nilsson, Anneli Ozanne

**Affiliations:** 1 University of Gothenburg, Sahlgrenska Academy, Institute of Health and Care Sciences and Centre for Person-Centred Care, and Palliative Centre at the Sahlgrenska University Hospital Region Västra Götaland, Gothenburg, Sweden; 2 University of Gothenburg, Sahlgrenska Academy, Institute of Health and Care Sciences, Gothenburg, Sweden; 3 Princess Nourah, Bint Abdulrahman University, Medical-Surgical Department of Nursing College, Riyadh, Saudi Arabia; 4 Umeå University, Department of Nursing, Skellefteå, Sweden; 5 University of Gothenburg, Biomedical Library, Gothenburg University Library, University of Gothenburg, Gothenburg, Sweden; 6 University of Gothenburg, Sahlgrenska Academy, Institute of Health and Care Sciences and University of Gothenburg Centre for Person-Centred Care (GPCC), Gothenburg, Sweden; 7 University of Gothenburg, Sahlgrenska Academy, Institute of Health and Care Sciences, and Department of Neurology, Sahlgrenska University Hospital, Gothenburg, Sweden

**Keywords:** Delivery of health care, integrated, Diffusion of innovation, Implementation science, Palliative care, Review

## Abstract

**Objectives:**

Research suggests palliative care to be translated and integrated in non-specialized palliative care services throughout the palliative care continuum across populations and settings. A need has been identified to build on the existing research literature in order to design strong knowledge translation strategies that can be evaluated in future research. The aim was to map strategies for knowledge translation of a palliative approach to care into non-specialized palliative care services for adult patients. The objectives were to explore the primary research activities, the specific type of knowledge translation strategies used, the research designs and study settings for such evaluations along with the major results thereof, and to identify major research gaps in this area.

**Methods:**

A scoping review was performed to map the volume and characteristics of research literature (project registered in PROSPERO #2018 CRD42018100663). The ten-year period 2010 to 2019 was searched in six major databases for original articles published in English in which the knowledge translation of a palliative approach for adult patients was evaluated in non-specialized palliative healthcare settings, and all type of empirical data-based research designs. We excluded non-English, non-empirical articles, non-evaluation of knowledge translations, specialized palliative care settings, and other types of publications (i.e. non-original articles).

**Results:**

Most of the 183 included articles focused on patients with cancer who were dying in hospitals and in high income countries. Only 13 articles focused on early palliative care. A palette of different strategies was used to implement palliative care in non-specialist palliative settings; no strategy was identified as outstanding. The majority of the articles had unspecified essential components of the research designs.

**Conclusion:**

Previous suggestions for utilization of implementation science for knowledge translation of a palliative approach to care into non-specialized palliative care services are confirmed, and established knowledge translation theories can strengthen the field. To advance this specific field of knowledge, meticulously detailed reporting of studies is required as related to research designs, clarifications of contextual influences and mechanisms at work. Specific systematic reviews and meta-syntheses in the field are merited.

**Supplementary Information:**

The online version contains supplementary material available at 10.1186/s12904-022-00929-0.

## Introduction

Today, it is well known that the vast majority of populations across countries will have need of palliative care due to a variety of long-term conditions for longer and shorter periods at the end-of-life [1]. Enhancing quality of life through appropriate care in this part of life is an issue related to human rights [2], public health and the striving for equity in care at end-of-life [3]. To reach this human rights goal, there is global consensus about prioritizing citizens’ access to palliative care [4]. Here, we refer to palliative care as an “active holistic care” to relieve suffering and distress for people with progressive life-limiting illness or “a condition that carries a high risk of mortality, negatively impacts quality of life and daily function, and/or is burdensome in symptoms, treatments, or caregiver stress and especially of those near the end of life” and that it “is applicable throughout the course of an illness” [[5], p. 761]. This will be an increasingly challenging societal issue, as the population in need of care in the latter part of life is estimated to increase due to an increase in annual deaths [6].

Fittingly, there is increasingly growing research-based evidence for ‘organized responses to end of life issues’ to accomplish this, i.e. a broad scope of palliative care interventions [[7], p. 1]. This emphasizes the need to effectively move this research-based knowledge to palliative care actions across healthcare services and especially outside those specialized in palliative care [2]. This can be realized by the practices of integrating a palliative approach to the care provided [8], and based on current research this is repeatedly argued for [9] and facilitated in several continents and countries [10] but found challenging [11, 12]. Evidence from current research especially supports such integration earlier on for patients [13, 14] in contrast to the more common practice of applying a palliative approach mostly for people who are dying [15]. Indeed, there are examples of successful early integration of palliative care [16]. However, contradictory results are also reported in the way physicians acknowledge the relevance of early palliative care, while at the same time only a minority of patients who might benefit from it are actually receiving it, even though integrated palliative care may have been of interest to them [17].

Given the current recommendations for early integration of a palliative approach to care for patients with cancer [18] and the recent suggestions to extend palliative care to serious health-related suffering [5], there is a special need for appropriate knowledge translation strategies applicable to palliative care, and particularly to early integration in the trajectories of patients with progressive life-limiting conditions. In this way, the scope of palliative care is wider than advance care planning, for example, with a specific literature [19]. However, while the research on implementation of palliative care in long-term care facilities has been synthesized [20], an organized overview of individual studies on knowledge translation strategies for a palliative approach to care across settings is lacking.

The literature about spreading new knowledge and sustaining innovations into action in healthcare services and organizations is vast [21] and a number of frameworks have been put forward to guide its processing. Here we use knowledge translation and implementation to facilitate the sustainable use and integration of new knowledge (i.e. innovation) synonymously with “a dynamic and iterative process that includes synthesis, dissemination, exchange and ethically-sound application of knowledge to improve the health […], provide more effective health services and products and strengthen the health care system” [22]. Determining the gap between what is known and what is done in practice is fundamental, with subsequent development and application of appropriate strategies that will overcome barriers to using this knowledge and facilitate its actual use [23]. In the knowledge translation process, it is especially important to consider interrelationships between evidence, context and facilitation [24]. The relevance of such implementation science perspectives for palliative care have been demonstrated [25]. Moreover, there is empirical research into strategies for knowledge translation of a palliative approach in non-specialized palliative care services but barriers to its integration have been identified across education, clinical knowledge translation and policy domains [26]. There is a need to build on the existing research literature in order to design strong knowledge translation strategies that can be evaluated in future research.

### Aim and objectives

The aim was to map strategies for knowledge translation of a palliative approach to care into non-specialized palliative care services for adult patients. The objectives were to explore the primary research activities, the specific type of knowledge translation strategies used, the research designs and study settings for such evaluations along with the major results thereof, and to identify major research gaps in this area.

### Research questions

1. What are the study aims? What research designs have been applied? Who are the participants and what are the study settings? Which part of the palliative care continuum is focused on?
2. What are the sources of evidence for the palliative approach? What are the strategies used to support knowledge translation of a palliative approach into non-specialized palliative care services and the sources of evidence for these strategies?
3. What are the outcomes of these strategies to support knowledge translation?

## Methods

### Design

As part of an initiative to identify and synthesize knowledge pertaining to efficacy and processes for knowledge translation of a palliative approach, we designed a scoping review [27–29] to map the volume and characteristics of research literature about knowledge translation strategies in the decade 2010–2019 with the influence of early integrated palliative care [13]; as part of an overall review project (registered in PROSPERO #2018 CRD42018100663). We especially accounted for complexities and heterogeneities in the literature, and aimed to present an inclusive representation of relevant studies [27]. All phases of the literature review process were discussed and decided in the research team, including challenges and uncertainties [29]. For the reporting, the Preferred Reporting Items for Systematic Reviews and Meta-Analysis (PRISMA) extended literature review guidelines for scoping reviews [30] were used. From the overall project, a systematic review related to knowledge translation of palliative care for children is reported elsewhere [31] and here we focus on the more extensive research related to adults.

### Search strategy

Two expert healthcare librarians were included in the team for the design of the electronic search strategy and these librarians also conducted the searches, all with the input of the other researchers with expertise in palliative care and review methodology. The search strategy was reviewed for accuracy using the Peer Review of Electronic Search Strategies criteria [32] and developed according to population, concept and context:

- Population: adult patients in need of health care

- Concept: knowledge translation strategies of a palliative approach, and

- Context: non-specialized palliative care services.

Accordingly, the concepts “knowledge translation”, “palliative care” and “health services” and their corresponding index terms with synonyms were applied for the search strategies (Table 1). We searched the databases PubMed, Scopus, PsycINFO, CINAHL, Cochrane and AgeLine from January 1, 2010 to December 31, 2019 to identify relevant studies, and the search terms applied to the databases are provided in Supplementary file 1. The delimitation to literature post 2009 was motivated by the seminal publication by Temel and co-workers in 2010 [13], possible influencing a shift in the field. Language restriction was English.

**Table 1:** Search concepts

Knowledge translation	AND	Palliative care	AND	Health services
implementation science or implementation research or diffusion of innovation or knowledge translation or knowledge transfer or knowledge exchange or improvement science or translational medical research or program evaluation or quality improvement or implementation or implementing		palliative care or palliative or hospice or hospices or hospice and palliative care nursing or palliative medicine or terminal care or hospice care or end of life or withholding treatment or supportive care or comfort care		health service or health services or university medical center or university medical centers or academic medical center or academic medical centers or hospital or hospitals or hospital unit or hospital units or nursery or nurseries or residential facility or residential facilities or primary healthcare or primary health care or primary care or community health services

For the detailed search strings, see Supplementary file 1

### Data sifting

Eligibility criteria were (a) original articles published in English between 2010 and 2019, (b) in which the *concept* knowledge translation of a palliative approach (c) for adult patients (18 years and older) (*population*) was (d) investigated in non-specialized palliative healthcare settings (*context*), and (e) investigated in all types of empirical data-based research designs. We excluded non-English, non-empirical (theoretical and discussion) articles, non-evaluation of knowledge translations, specialized palliative care settings and brief research reports, editorials, theses, abstracts, proceedings, books and book chapters.

Citations were directly imported to a citation managing software to remove duplicates. The list of all titles and abstracts identified were divided into two parts, and two researchers separately and independently screened each part of the list using the text mining tool RAYYAN (rayyan.qcri.org) [33]. The identified citations were then assessed for eligibility based on full text articles, also carried out by two independent researchers. At all stages, discrepancies and uncertainties in the researchers’ assessments were discussed and resolved, firstly between the two assessors and subsequently by the research team in order to reach consensus. Finally, an additional manual search based on the citations and references for included articles was performed.

### Data charting process

For the data extraction (in scoping methodology labelled data charting) details from eligible articles were extracted according to pre-designed data extraction codes using the software programme NVivo 12. The codes used were chosen to organize the data according to the review’s objectives in a stepwise process of trying out and refining the codes. The guiding principle was to retrieve no more data than was needed to achieve the objectives of this scoping review. The use of the software programme facilitated the charting of explicit statements in the articles and avoided interpretations. No contact was made with the authors of the articles. One researcher was responsible for charting all the included articles, but research team meetings were regularly held during this process to discuss the data charting and identify especially difficult cases and items to be re-assessed for eligibility.

### Analysis and synthesis of results

The included articles were collated and summarized, with a report of the results. The studies were categorized according to research approach, research design, disease population, sampling principles, participant categories, settings, and data sources. To categorize study-focus in relation to a palliative care continuum the studies were grouped into unspecified palliative care, end-of-life care or care for the dying, palliative care and end-of-life care, and early palliative care (including a palliative approach). Sources of evidence for the palliative care approach and for palliative knowledge translation strategies were extracted from the studies.

To categorize the knowledge translation strategies we applied a range of strategies found to be useful according to Wallin [34], adapted from Grol and Grimshaw [35] and grouped into: (a) dissemination or educational strategies, (b) social interaction strategies, (c) decision-support strategies and (d) organizational strategies. We added a fifth group for “other” strategies. When necessary, the specified strategies within each of the four groups were adapted to palliative knowledge translation. In this way, we added consultations, facilitators, support services or helplines as a social interaction strategy, and also added “other strategies” to each of the four strategy groups.

The knowledge translations outcomes were grouped into successful, mixed and failed impact/effect of the strategies investigated. A strategy was deemed successful if there was significant improvement in the outcome variable, mixed if some parts were successful but not all, and failed if the outcome variable did not improve; when primary and secondary outcomes were stated this was based on the primary outcome, and when there was no distinction between primary and secondary outcomes this categorization was based on the majority of the outcome variables. An additional group of outcomes was the explication of knowledge translation facilitators and/or barriers. All these categorizations were based on expressions as reported in the articles.

## Results

**Fig. 1:**
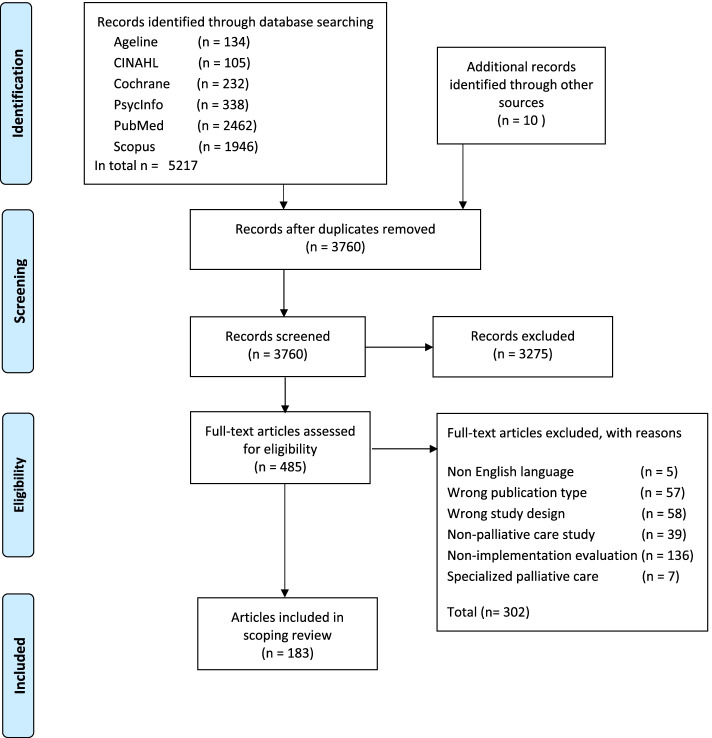
Review flow diagram

The total searches resulted in 5 227 citations, and following screening and full-text assessments 183 original empirical data-based articles were included for review (Fig. 1; see Supplementary File 2 for an overview of these articles, Supplementary File 3 for references, and Table 2 for study characteristics). During the ten-year period under review, between 12 and 27 articles were published annually, with 14 in 2010, 27 in 2017 and 12 in 2019.

**Table 2:** Characteristics of studies included (*n* = 183)

Study characteristic	Total
**Aim**^b^	
To examine impact of a PKTS^a^	70 (38.3%)
To evaluate effectiveness of a PKTS	29 (15.8%)
To optimize end-of-life care	22 (12.0%)
To describe professionals’ experience of a PKTS	22 (12.0%)
To assess feasibility/acceptability of a PKTS	16 (8.7%)
To present a PKTS	14 (7.7%)
To describe facilitators/barriers of a PKTS^b^	10 (5.5%)
**Research approaches**^c^	
Quantitative	90 (49.2%)
Qualitative	34 (18.6%)
Mixed-methods	59 (32.2%)
**Research designs**^d^	
Quasi-experimental^e^	24 (13.1%)
Experimental	14 (7.7%)
Quality Improvement	14 (7.7%)
Descriptive/explorative	7 (3.8%)
Observational	7 (3.8%)
Participatory	4 (2.2%)
Case study	4 (2.2%)
Other	16 (8.7%)
Unspecified	93 (50.8%)
**Sampling principle(s)**^d^	
Representative (random)	41 (22.4%)
Strategic/purposeful	28 (15.3%)
Convenient	18 (9.8%)
Consecutive	7 (3.8%)
Other	7 (3.8%)
Unspecified	108 (59.0%)
**Participant category**^d^	
Healthcare professionals	128 (70.0%)
Patients	86 (47.0%)
Family members	26 (14.2%)
Other	35 (19.1%)
**Disease population**^d^	
Cancer	55 (30.1%)
Dementia	16 (8.7%)
Cardiovascular diseases	11 (6.0%)
Other conditions	42 (22.9%)
Unspecified/unclear	102 (55.7%)
**Settings**^d^	
Hospital	103 (56.3%)
Residential care facilities	39 (21.3%)
Home care services	9 (4.9%)
Other	34 (18.6%)
Unspecified/unclear	24 (13.1%)
**Data sources**^d^	
Surveys	95 (51.9%)
Interviews, individual	66 (36.1%
Interviews, groups	33 (18.0%)
Patient medical records	34 (18.6%)
Existing register data	30 (16.4%)
Observations	18 (9.8%)
Other	57 (31.1%)
Unspecified/unclear	7 (3.8%)
**Palliative care continuum focus**^c^	
End-of-life care or care for the dying	57 (31.1%)
Palliative care *and* end-of-life care	25 (13.7%)
Early palliative care	13 (7.1%)
Palliative care unspecified	88 (48.1%)
**Sources of evidence for the palliative approach**^d^	
References to other papers	122 (66.7%)
Guidelines, laws, regulations	36 (19.7%)
WHO-definition	19 (10.4%)
No evidence for palliative approach	13 (7.1%)
**Sources of evidence for the PKTS**^d^	
References to other papers	73 (39.9%)
Frameworks	7 (3.8%)
Guidelines	3 (1.6%)
No evidence for implementation strategy	103 (56.3%)
**Types of knowledge translation strategies**^d^	
Dissemination or educational strategies	112 (61.2%)
Social interaction strategies	99 (54.1%)
Decision-support strategies	50 (27.3%)
Organizational strategies	87 (47.5%)
Other types of strategies	32 (17.5%)
**Results from evaluation of PKTS**^c^	
Successful	93 (50.8%)
Mixed	59 (32.2%)
Failed	16 (8.7%)
Results explicating influencing factors	15 (8.2%)

^a^PKTS; palliative knowledge translation strategy. ^b^The study

results explicated factors functioning as facilitators and/or barriers to

a PKTS. ^c^Each of the articles occur only once in these categories

^d^More than one of the categories can occur in each of the articles

^e^Includes before-and-after (pre-test, post-test) design

### Study aims, research designs, participants and study settings

Of the 183 articles reviewed, 54.1% aimed to investigate the impact or effectiveness of a knowledge translation strategy, 12.0% to describe professionals’ experience of applying one and the remaining to describe factors influencing it (5.5%), present such a strategy (7.6%) and optimize end-of-life outcomes (12.0%). A range of research designs were applied, of which quasi-experimental design was the most commonly specified (13.0%), while 50.8% of the studies had unspecified research designs (Table 2). Of the 99 articles identified with the aim of examining impact or effectiveness of a knowledge translation strategy, 34.3% were performed with either experimental or quasi-experimental research designs and 48.5% with unspecified research designs. Of the 22 articles aiming to describe professionals’ experience of knowledge translation strategies, 50.0% had unspecified research designs (Table 3). Sampling principles were unspecified in 59.0% (Table 2). The data sources for evaluating the knowledge translation strategies included interviews (54.1%; individual or group), surveys (50.3%), patient medical records (18.0%), existing registry data (15.9%) and observations (9.5%).

**Table 3:** Type of research designs in relation to categorization of study aims and type of knowledge translation strategies (*n* = 183)

	Types of Research Designs									
	Descriptive^a^	Observational	Experimental	Quasi- experimental	Participatory^b^	Case study	Quality improvement	Other	Unspecified	Total
**Aim categories**^c^										
To optimize EoL^d^ care	1 (0,5%)	0 (0.0%)	1 (0,5%)	1 (0,5%)	0 (0.0%)	0 (0.0%)	6 (3.3%)	0 (0.0%)	13 (7.1%)	22 (12.0%)
To present a PKTS	1(0,5%)	0 (0.0%)	0 (0.0%)	0 (0.0%)	0 (0.0%)	0 (0.0%)	2 (1.1%)	0 (0.0%)	11 (6.0%)	14 (7.6%)
To assess feasibility or acceptability of a PKTS	1 (0,5%)	1 (0,5%)	1 (0,5%)	0 (0.0%)	2 (1.1%)	1 (0,5%)	3 (1.6%)	2 (1.1%)	5 (2.7%)	16 (8.7%)
To describe professionals’ experience of PKTS	1 (0,5%)	2 (1.1%)	0 (0.0%)	1 (0,5%)	1 (0,5%)	1 (0,5%)	0 (0.0%)	5 (2.7%)	11 (6.0%)	22 (12.0%)
To examine impact of a PKTS	2 (1.1%)	3 (1.6%)	1 (0.5%)	14 (7.6%)	1 (0,5%)	1 (0,5%)	3 (1.6%)	7 (3.8%)	38 (20.8%)	70 (38.2%)
To evaluate effectiveness of a PKTS	0 (0.0%)	0 (0.0%)	11 (6.0%)	8 (4.4%)	0 (0.0%)	0 (0.0%)	0 (0.0%)	0 (0.0%)	10 (5.4%)	29 (15.8%)
To describe facilitators or barriers of a PKTS	1 (0,5%)	1 (0,5%)	0 (0.0%)	0 (0.0%)	0 (0.0%)	1 (0.5%)	0 (0.0%)	2 (1.1%)	5 (2.7%)	10 (5.5%)
**Types of knowledge translation strategies**^e^										
Dissemination or educational strategies	4 (2.2%)	1 (0.5%)	14 (7.6%)	15 (8.2%)	1 (0.5%)	1 (0.5%)	8 (4.4%)	7 (3.8%)	61 (33.3%)	112 (61.2%)
Social interaction strategies	5 (2.7%)	1 (0.5%)	6 (3.3%)	17 (9.3%)	3 (1.6%)	3 (1.6%)	7 (3.8%)	13 (7.1%)	44 (24.0%)	99 (54.1%)
Decision-support strategies	0 (0.0%)	3 (1.6%)	4 (2.2%)	9 (4.9%)	0 (0.0%)	1 (0.5%)	8 (4.4%)	4 (2.2%)	21 (11.5%)	50 (27.3%)
Organizational strategies	4 (2.2%)	1 (0.5%)	9 (4.9%)	7 (3.8%)	1 (0.5%)	3 (1.6%)	6 (3.3%)	10 (5.5%)	46 (25.1%)	87 (47.5%)
Other types of strategies	1 (0.5%)	2 (1.1%)	2 (1.1%)	1 (0.5%)	0 (0.0%)	0 (0.0%)	0 (0.0%)	3 (1.6%)	23 (12.6%)	32 (17.5%)

^a^Includes explorative designs. ^b^Includes action research designs. ^c^Each of the articles occur only once in these categories. ^d^EoL; end of life. ^e^More than one of the categories can occur in each of the articles

Healthcare professionals were the most common stakeholder group involved as participants in 70.0% of the articles (21.9% involved professionals and also one or several other groups), while 47.0% involved patients (13.7% involved patients and also one or several other groups). Among specified populations, cancer was the most common and involved in 30.1% of the articles (5.5% had cancer samples combined with other populations). Populations with dementia and cardiovascular diseases were specified in 8.7% and 6.0% of the articles respectively. Unspecified or unclear patient populations were included in 55.7% of the articles (Table 2).

**Fig. 2:**
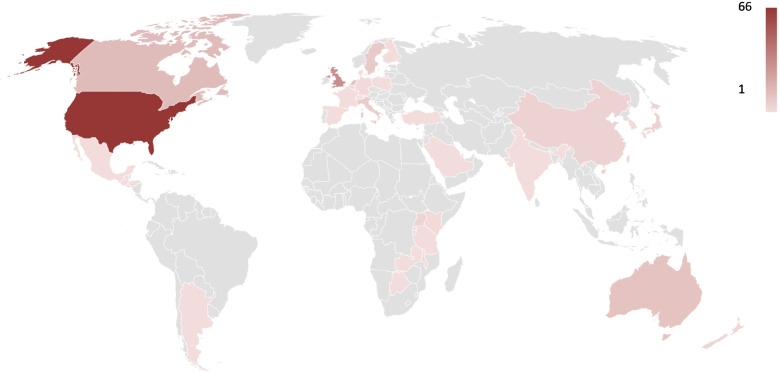
Countries represented with data in the studies reviewed; from 1 to 66 (red colour scale; grey = country not represented in any article) articles per country (*n* = 183)

All continents and 35 countries were represented in the studies, with 43.7% of data coming from North America and 39.9% from Europe (Fig. 2). In 97.8% of the articles one country was included (four articles had data from 4–10 countries). In terms of the global Human Development Index (HDI), of the 35 countries represented, 22 were very high, 3 high, 6 middle and 4 low country levels. The three most common countries were USA, United Kingdom and Canada with 36.1%, 17.5% and 7.7% respectively. Hospital was included as a setting in 56.3% and residential care (including nursing homes) in 21.3% of the articles.

### Focus of the palliative care continuum and sources of evidence for its interventions

In terms of a palliative care continuum, 48.0% investigated unspecified palliative care, 31.1% end-of-life care or care for the dying, 13.7% palliative and end-of-life care, and 7.1% focused on early palliative care (Table 2). There were seven (53.8%) articles with unspecified research designs among the 13 articles focusing on early palliative care, and a breadth existed in how early was practiced. Some considered palliative care applicable at diagnosis of a serious disease, others when patients were considered to have an advanced illness. There were examples of studies applying palliative care alongside curative treatment or interventions. Moreover, estimated life expectancy (e.g. < 1 year) or symptom burden was also used to distinguish when to initiate palliative care.

The evidence base for the palliative care interventions was stated in 92.9% of the articles, mostly referring to other studies (66.7%), to guidelines, laws, and regulations (19.7%), and to the WHO definition of palliative care (10.4%; Table 2). Seven articles (3.8%) referred to more than one source of evidence. All 13 articles focusing on early palliative care stated a source of evidence for the palliative care intervention and 11 (84.6%) also had a reference to other studies.

### Sources of evidence and knowledge translation strategies

The evidence base for the implementation strategy was presented in less than half (45.3%) of the articles, mostly referring to other studies (39.9%). A minority referred to frameworks (3.8%) or guidelines (1.6%) (Table 2). Three articles (1.6%) referred to more than one source of evidence. Only three articles focused on early palliative care, stating a source of evidence for the strategy, and all three included a reference to other studies.

All the categorized knowledge translation strategies [34] were represented in the articles (Table 4), for which single strategies were used (31.7%), as well as a combination of up to eight strategies (0.5%) with a median of five strategies (Table 5). *Conferences, courses and workshops* was the most commonly used knowledge translation strategy in 50.3% of the studies, as part of a multimodal strategy or a single strategy. *Consultations, facilitators, support services or helplines*, *Multi-professional group meetings*, *Guidelines, toolkits, policies, tech tools* and *Other social interaction strategies* were used in 29.5%, 23.0%, 21.9% and 20.8% of the studies respectively (Table 4). Of the 13 articles focusing on early palliative care, 8 (61.5%) used *Conferences, courses, workshops*, 3 (23.1%) had a combination of two strategies and 7 (53.8%) had four to six strategies combined.

**Table 4:** Distribution of strategies used for the knowledge translation (*n* = 183)

**Knowledge translation strategies**^a^	**Frequencies**	**Results of the strategies**^**b,c**^			
		Successful	Mixed	Failed	Explicating influencing factors^d^
**Dissemination or educational strategies**					
Conferences, courses, workshops	92 (50.3)	54 (58.7%)	28 (30.4%)	7 (7.6%)	3 (3.3%)
Educational materials	25 (13.7%)	12 (48.0%)	9 (36.0%)	3 (12.0%)	1 (4.0%)
Educational outreach visits	17 (9.3%)	11 (64.7%)	6 (35.3%)	0 (0.0%)	0 (0.0%)
Mass media campaigns or other public campaigns	3 (1.6%)	3 (100.0%)	0 (0.0%)	0 (0.0%)	0 (0.0%)
Other dissemination or educational strategies	29 (15.8%)	18 (62.1%)	9 (31.0%)	1 (3.4%)	1 (3.4%)
**Social interaction strategies**					
Consultations, facilitators, support services or helplines	54 (29.5%)	32 (59.3%)	16 (29.6%)	3 (5.6%)	3 (5.6%)
Multi-professional group meetings	42 (23.0%)	20 (47.6%)	17 (40,5%)	3 (7.1%)	2 (4.8%)
Feedback to stakeholders	5 (2.7%)	1 (20.0%)	4 (80.0%)	0 (0.0%)	0 (0.0%)
Other social interaction strategies	38 (20.8%)	18 (47.4%)	14 (36.8%)	0 (0.0%)	6 (15.8%)
**Decision-support strategies**					
Reminders or triggers, check-lists, templates	26 (14.2%)	13 (50.0%)	11 (42.3%)	1 (3.8%)	1 (3.8%)
Computerized decision support	2 (1.1%)	1 (50.0%)	1 (50.0%)	0 (0.0%)	0 (0.0%)
Other decision-support strategies	27 (14.8%)	13 (48.1%)	11 (40.7%)	2 (7.4%)	1 (3.7%)
**Organizational strategies**					
Guidelines, toolkits, policies, tech tools	40 (21.9%)	18 (45.0%)	18 (45.0%)	3 (7.5%)	1 (2.5%)
Pathways or programmes	31 (16.9%)	14 (45.2%)	11 (35.5%)	0 (0.0%)	6 (19.4%)
Financial interventions	6 (3.3%)	4 (66.7%)	1 (16.7%)	0 (0.0%)	1 (16.7%)
Other organizational strategies	25 (13.7%)	9 (36.0%)	12 (48.0%)	2 (8.0%)	2 (8.0%)
Other types of strategies	32 (17.5%)	18 (56.3%)	10 (31.3%)	3 (9.4%)	1 (3.1%)

^a^Strategies and grouping according to Wallin; adapted from Grol and Grimshaw. ^b^Results from studies include the strategy as

both a single or multiple strategy; more than one of the strategy variables can occur in each of the articles. ^c^Results from studies with

experimental, quasi-experimental, comparative and theorizing/interpretive study designs include the strategy as both a single or multiple

strategy; more than one of the strategy variables can occur in each of the articles. ^d^Factors influencing knowledge translation processes;

most often grouped into facilitators and barriers

**Table 5:** Evaluation results in relation to the number of knowledge strategies that were combined in one evaluation (*n* = 183)

		**Results of the strategies**			
Number of knowledge translation strategies combined	Frequency	Successful	Mixed	Failed	Explicating influencing factors
1	58 (31.7%)	29 (50.0%)	14 (24.1%)	6 (10.3%)	9 (15.5%)
2	42 (23.0%)	17 (40.5%)	15 (35.7%)	8 (19.0%)	2 (4.8%)
3	33 (18.0%)	18 (54.5%)	11 (33.3%)	2 (6.1%)	2 (6.1%)
4	23 (12.6%)	16 (69.6%)	6 (26.1%)	0 (0.0%)	1 (4.3%)
5	9 (4.9%)	4 (44.4%)	5 (55.6%)	0 (0.0%)	0 (0.0%)
6	11 (6.0%)	5 (45.5%)	5 (45.5%)	0 (0.0%)	1 (9.1%)
7	6 (3.3%)	4 (66.7%)	2 (33.3%)	0 (0.0%)	0 (0.0%)
8	1 (0.5%)	0 (0.0%)	1 (100.0%)	0 (0.0%)	0 (0.0%)

### Outcomes

In total, the results of the evaluations of the knowledge translation strategies were reported as successful in 50.1%, mixed in 32.8% and failed in 8.7% of the studies, while in 8.2% of the articles, factors influencing knowledge translation processes were identified (Table 2). Although 56.3% of the articles lacked evidence for the knowledge translation strategy applied, the results of about half the total number of articles (49.5%) were reported as successful. Single knowledge translation strategies were reported successful in 50.0% of the articles and the highest proportion of successful results was derived from a combination of four strategies. Of the remaining studies employing combined knowledge translation strategies, 40.5% to 66.7% reported successful results, apart from the study with a combination of eight strategies, which had mixed results (Table 5). Of the 13 articles focusing on early palliative care, 7 (53.8%) reported successful results and 5 mixed (38.5%). The successful results were reported as related to both single (2 articles) and multimodal (5 articles; combinations of 4–6) strategies. For the 14 articles with experimental and quasi-experimental designs successful results were derived from a combination of 2–8 of a total 9 different strategies (Table 3). The successful results also derived from seven quality improvement designs and 59 unspecified research designs.

Successful results were reported for all types of knowledge translation strategies but almost all also had mixed and failed results, with some articles identifying influencing factors on the knowledge translation process. All strategies were frequently used and reported as successful in between 45.0% and 66.7% of the articles. However, there were three exceptions: *Mass media campaigns or other public campaigns* were successful in all three articles that evaluated these, and *Feedback to stakeholders* and *Other organizational strategies* were primarily reported with mixed results (80.0% and 48.0% respectively). Seven of the knowledge translation strategies were not reported as failed in any articles and no strategy was failed in more than 12.0% of the articles (Table 4). In the 13 articles focusing on early palliative care with successful results, the most common strategies used were *Conferences, courses and workshops* (5 articles, 38.4%), *Consultations, facilitators, support services or helplines* (4 articles, 30.8%) and *Multi-professional group meetings* (3 articles, 23.1%).

## Discussion

### Main results

This scoping review reveals that although many different strategies were used to implement palliative care in non-specialist palliative settings, no strategy was identified as outstanding. Still, among the strategies used in more than 20% of the studies there were several with primarily successful results (> 45% successful): Conferences, courses, workshops; Consultations, facilitators; support services or helplines; Multi-professional group meetings; Other social interaction strategies; and Guidelines, toolkits, policies, tech tools. A combination of such strategies might be useful to further inquire. The majority of the articles (50.8%) had unspecified research designs, sampling principles (59.0%) and disease populations (55.7%). Moreover, most papers concerned implementation in hospitals (56.3%) and only a few (7.1%) were dedicated to an early palliative phase. However, among the 13 articles focusing on early palliative care, the tendency was to combine two or several strategies.

About half of the 183 articles reviewed had broad study aims related to the “impact” of a knowledge translation strategy (38.3%) or only stated a clinical outcome without clarifying a research objective (12.0%). Although 54% of the papers aimed to investigate impact or effectiveness of a knowledge translation strategy, a minority had experimental or quasi-experimental design. Hence, the results paint a picture of a research field in need of increased rigour.

Participants were patients in only 47% of the studies, which could be because patients with palliative care needs are vulnerable and might not have the strength to participate. In contrast, 70.0% of the articles included health professionals as participants, meaning this stakeholder group was well represented. Including health professionals’ perspectives in the evaluation of knowledge translation strategies is unequivocally appropriate, as the whole aim is to implement strategies in practice. However, in order to provide person-centred care, the patients’ care needs have to be acknowledged, and a strategic infrastructure approach developed to facilitate patient and public involvement in the research [36].

The vast majority of the reviewed articles retrieved data from high income countries – only ten middle- and low-income countries were represented. This reiterates the importance of increasing the evidence for palliative care as related to contextual features in diverse countries and especially countries with limited incomes [37, 38]. Moreover, in the reviewed articles it was difficult to identify contextual features that might influence how to successfully design a strategy for palliative care knowledge translation, for example, financing systems for healthcare or sociocultural values related to informal and volunteer care. The results also raise questions about what contextual features affect and might be affected in palliative care knowledge translation, possible interaction effects between the mechanisms of impact in the palliative intervention and the knowledge translation strategies, and if and how they influence outcomes [39].

Hospital was the setting for the majority of the reviewed articles (56.3%) and this is understandable considering that some patients during their final period of life will need healthcare responses to sudden onset of distress and symptoms, irrespective of whether these are critical or manageable. This could motivate the integration of palliative care across clinical specialities in hospitals, including emergency departments [40] and intensive care [41]. Such knowledge translation in hospitals is relevant. However, to facilitate integration of early palliative care there is also a special need to include residential care settings [42] and primary care services [43] as arenas for this knowledge translation.

It is problematic that contextual features are unsystematically reported, especially from a knowledge translation perspective, giving special emphasis on careful elaboration of context [23, 24] in the direct study setting, as well as how this is mediated societally and culturally – the latter perhaps being of special importance in a value loaded field like palliative care. For consideration of contextual influences in knowledge translation processes, the use of quality improvement, participatory action research and other types of processual research designs might be feasible for taking contextual features into account. Accordingly, research designs are proposed to include methodologies that guide, for example, how to navigate complex social systems and facilitate co-creation with various stakeholders [44, 45]. In this way, the knowledge translation process can be related to practice concerns, views of reality, sources of evidence and tensions in health systems and similar influencing aspects [44].

A public health perspective on palliative care [3] can also shed light on the importance of palliative knowledge translation in primary care, which entails not a replacement of but an addition to collaboration with specialized palliative care services and consultation teams [46, 47]. More knowledge is needed as to which strategies are generic and feasible across various types of settings and palliative interventions, and which strategies and interventions are context specific.

In most studies, the palliative care continuum was not specified, which meant it could be difficult to evaluate the appropriateness of the implementation for both early palliative care and for people who are dying. Early palliative care is also an exception in this field, represented in only 7.1% of the articles. This will probably hamper the use of the recommendations for early and integrated palliative care [18], at least for research-based integration and knowledge translation.

Descriptions of the palliative care interventions to be translated and/or integrated into care practice were often too broad, and lacked the specific detail needed to achieve transparency to guide practice. To resolve this, the combined empirically and conceptually based taxonomy by Clark and co-workers [7] distinguishing ten types of palliative care interventions could be useful. For similar purposes, we echo previous recommendations for future research to explicate the specific knowledge translation strategies applied [20], and to conceptually relate these to an established scientific implementation framework [34, 35].

The most commonly used types of strategies were *Dissemination or educational strategies* and *Social interaction strategies,* and the vast majority of the reviewed articles combined two or more strategies. Educational strategies that enable social interactions might be especially useful in facilitating learning, while opportunities to create contextual change can be important in helping participants enact according to the knowledge acquired through the educational initiative [48]. Moreover, the inclusion of educational strategies can be regarded as a response to the well identified multiple perspectives of healthcare professionals on what palliative care entails [49]. However, it raises questions about the kind of learning activities that are applied in ways that allow for change in perspectives related to palliative care and end of life care. All learning activities may not pave the way for transformative understanding related to aspects such as suffering, death, dying and grief. This emphasises that the applied strategies should be in line with a knowledge translation perspective [23] rather than knowledge transfer.

A wide range of specific knowledge translation strategies have been evaluated, with no clear identifiable pattern for strategies to be useful. Indeed, roughly every other reviewed study did not report evidence for the choice of strategies applied. In addition, a variety of outcomes were reported and primary outcomes were not always stated, meaning meta-analysis of specific knowledge translation strategies will be challenging. There is no clear identifiable pattern for strategies to be recommended from this scoping review. In future research, there is a need for careful consideration of which strategies should be combined and what outcomes could be achieved.

Of the strategies that were used in more than 20 papers, between 36–59% were successful. Between 30–48% had mixed results and 4–12% failed to improve palliative care. The reason for success in only about half of the studies could be weak design, unspecified phase of palliative care continuum or unclear palliative care intervention and knowledge translation strategies. Another reason for not being successful could be that the evidence base for the implementation strategy was presented in only 43.7% of the articles. We reiterate previous suggestions for considering how implementation science and established knowledge translation theories can be applied to strengthen the field, alongside with clarifications of contextual influences and mechanisms at work [50, 51]. In so doing, it is important to consider the inherent limitations in the metaphor of ‘translation’, as it is highly challenging to drive new knowledge into practice in the ways evidence tends to be negotiated – as in the case of palliative care in non-specialized settings. For this reason, various perspectives of knowledge as action [44] will be useful, including professionals’ practical wisdom and clinical judgement, as well as partnerships between different stakeholders on meso and macro levels [52]. Innovative strategies may also be considered, such as large-scale virtual events [53].

### Limitations

The review is based on research from only one decade; however this was especially motivated by the influential work on early integrated palliative care published in 2010 [13]. This provides motivation for future reviews to analyse how this field is developing. Due to logistic reasons, the language of the included articles was restricted to English, possibly omitting other valuable perspectives and societal contexts. In our searches, we used the term ‘knowledge translation’, which could have resulted in a large body of general papers that did not explicitly report on implementation strategies. The omission of knowledge translation as a search term could have contributed to a more stringent result. However, the search strategy was developed to review the broader field of palliative knowledge translation, and for this reason we did not delimit the searches to, for example, implementation strategies with related facilitators and barriers. This might explain why only 5.5% of studies reported explication of factors influencing a palliative care knowledge translation strategy. Moreover, the identified research was diverse, with approximately half the studies reporting unspecified research designs, sampling principles, disease population and clarification of the source of evidence for the evaluated knowledge translation strategies.

Notably, as a result of the scoping methodology applied, this review does not incorporate assessments of the quality or weight of evidence in the included articles. Likewise, there is no assessment of how research ethics was applied. In several articles the primary and secondary outcomes were unclear, while in others it was not clear if the results were to be considered successful, mixed or failed. The majority of articles did not distinguish between primary and secondary outcomes at all.

All phases of the literature review process were discussed and decided upon by the research team. In this way, the formal requirements for inclusion and interpretation were complemented by informal processing within the team. For purposes of transparency, we repeatedly strove to unpack considerations and decisions and update the method applied with clarifications where relevant to avoid unexplicated informal judgements [54].

### Conclusion

Integrating a palliative approach in the scope of healthcare services available to patients requiring palliative care earlier on in their disease trajectories is a well-documented need. However, the results of this review do not promise to facilitate this through research activities. The vast majority of research in this field has evaluated knowledge translation in hospitals for patients with cancer who are dying or in end-of-life care. However, questions can be raised as to how applicable the results of these studies are in meeting the need for translating knowledge of a palliative approach to care earlier on in the trajectory for patients with various progressive and life-limiting conditions. Indeed, there is a special need to contribute with knowledge in order for this group to be cared for in their preferred location of care, which is more commonly ordinary homes and residential care settings rather than hospitals. Thus, to increase applicability, this research also needs to be performed in patients’ preferred locations of care and within the various healthcare services in place.

Lack of stringency in the reporting is an overarching characteristic of this specific field that will affect the extent to which knowledge synthesis can inform practice, as well as policy. One hypothesis is that contextual features that were not reported may be important in the knowledge translation of a palliative approach to non-specialized palliative services. This review thus reiterates Greenhalgh and co-workers’ [21] methodological recommendations for knowledge translation research, based on their comprehensive review from 2004, that call for meticulously detailed reporting of studies that are theory-driven, process oriented, collaborative and coordinated evaluations in a variety of contexts, and that feature participatory, multidisciplinary and multi-method design. Specific systematic review(s) of studies with evaluative research designs and study aims about evaluation of impact and effectiveness of knowledge translation strategies, as well as meta-synthesis of factors influencing knowledge translation processes, are merited. In addition, a systematic review of knowledge translation of early palliative care is important, even if there seems to be limited research in this specific field.

## Supplementary Information

**Additional file 1: Supplementary File 1.** Search strings applied in the six data bases. **Supplementary File 2.** Overview of the included studies. **Supplementary File 3.** References to the included studies.

## Data Availability

The datasets used and/or analyzed during the study are available from the corresponding author upon reasonable request.

## Abbreviations

PKTS: Palliative knowledge translation strategy
EoL: end of life
