# Supplementary material for: Strategies for knowledge translation of a palliative approach outside specialized palliative care services: a scoping review
Source: BMC Palliat Care. 2022 Mar 22;21:39. doi: 10.1186/s12904-022-00929-0 (PMC8939083; doi:10.1186/s12904-022-00929-0)
Supplement: Supplementary file 1 — Additional file 1: Supplementary File 1. Search strings applied in the six data bases. Supplementary File 2. Overview of the included studies. Supplementary File 3. References to the included studies. [file 12904_2022_929_MOESM1_ESM.docx]

Supplementary File 1. Search strings applied in the six data bases

**PubMed**

#1 “Health Service”[tiab] OR “Health Services”[tiab] OR “University Medical Center”[tiab] OR “University Medical Centers”[tiab] OR “Academic Medical Centers”[tiab] OR “Academic Medical Center”[tiab] OR “hospital”[tiab] OR “Hospitals”[tiab] OR “Hospital unit”[tiab] OR “Hospital units”[tiab] OR “Nursery”[tiab] OR “Nurseries”[tiab] OR “Residential Facilities”[tiab] OR “Residential Facility”[tiab] OR “Primary Healthcare” [tiab] OR “Primary care”[tiab] OR “Primary Health care”[tiab]

#2 (Health Services[**MeSH Terms**] OR Academic Medical Centers[**MeSH Terms**] OR Hospital Units[**MeSH Terms**] OR Hospitals[**MeSH Terms**] OR Nurseries[**MeSH Terms**] OR Residential Facilities[**MeSH Terms**] OR Primary Health Care[**MeSH Terms**] OR Community Health Services[**MeSH Terms**])

#3 #1 OR #2

#4 **(implementation science[MeSH Terms] OR diffusion of innovation[MeSH Terms] OR translational medical research[MeSH Terms] OR program evaluation[MeSH Terms] OR quality improvement[MeSH Terms])**

#5 **(implementation[tiab] OR implementing[tiab] OR "diffusion of innovation"[tiab] OR "knowledge translation"[tiab] OR "knowledge transfer"[tiab] OR "knowledge exchange"[tiab] OR "improvement science"[tiab])**

**#6 #4 OR #5**

#7 (Palliative Care[MeSH Terms] OR Hospice and Palliative Care Nursing[MeSH Terms] OR Palliative Medicine[MeSH Terms] OR Terminal Care[MeSH Terms] OR Hospice Care[MeSH Terms] OR Hospices[MeSH Terms])

#8 (Palliative[tiab] OR Hospice[tiab] OR Hospices[tiab] OR "End of life"[tiab] OR "End-of-life"[tiab] OR "withholding treatment"[tiab] OR "supportive care"[tiab] OR "comfort care"[tiab])

#9 #7 OR #8

#10 #3 AND #6 AND #9

#11 Filter: English, January 1 2010 – December 31 2019

**Scopus**

#1 TITLE-ABS-KEY (“Health Service” OR “Health Services” OR “University Medical Center” OR “University Medical Centers” OR “Academic Medical Centers” OR “Academic Medical Center” OR “hospital” OR “Hospitals” OR “Hospital unit” OR “Hospital units” OR “Nursery” OR “Nurseries” OR “Residential Facilities” OR “Residential Facility” OR “Primary Healthcare” OR “Primary care” OR “Primary Health care” OR “Community Health Services”)

#2 TITLE-ABS-KEY ("implementation science" OR "implementation research" OR "diffusion of innovation" OR "knowledge translation" OR "knowledge transfer" OR "knowledge exchange" OR "improvement science" OR “translational medical research” OR “translational medicine” OR “program evaluation” OR “quality improvement” OR implementation OR implementing)

#3 TITLE-ABS-KEY (Palliative OR Hospice OR Hospices OR “Terminal Care” OR "End of life" OR "End-of-life" OR "withholding treatment" OR "supportive care" OR "comfort care")

#4 #1 AND #2 AND #3

#5 Filter: English, January 1 2010 – December 31 2019

**Cochrane Library**

#1 TI/AB/KW: “Health Service” OR “Health Services” OR “University Medical Center” OR “University Medical Centers” OR “Academic Medical Centers” OR “Academic Medical Center” OR “hospital” OR “Hospitals” OR “Hospital unit” OR “Hospital units” OR “Nursery” OR “Nurseries” OR “Residential Facilities” OR “Residential Facility” OR “Primary Healthcare” OR “Primary care” OR “Primary Health care”

#2 KW: (Health Services OR Academic Medical Centers OR Hospital Units OR Hospitals OR Nurseries OR Residential Facilities OR Primary Health Care OR Community Health Services)

#3 #1 OR #**2**

#4 TIABKW: ("implementation science" OR "implementation research" OR "diffusion of innovation" OR "knowledge translation" OR "knowledge transfer" OR "knowledge exchange" OR "improvement science" OR implementation OR implementing)

#5 KW: (diffusion of innovation OR translational medical research OR program evaluation OR quality improvement)

#6 #4 OR #5

#7 TIABKW: (Palliative OR Hospice OR Hospices OR Hospice OR Hospices OR "End of life" OR "End-of-life" OR "withholding treatment" OR "supportive care" OR "comfort care")

#8 KW: (Palliative Care OR Hospice and Palliative Care Nursing OR Palliative Medicine OR Terminal Care OR Hospice Care OR Hospices)

#9 #7 OR #8

#10 #3 AND #6 AND #9

#11 Filter: English, January 1 2010 – December 31 2019

**CINAHL (Ebsco)**

#1 MH (Health Services OR Academic Medical Centers OR Hospital Units OR Hospitals OR Nurseries, Hospital OR Residential Facilities OR Primary Health Care OR Community Health Services)

#2 TI ( ("Health Service” OR “Health Services” OR “University Medical Center” OR “University Medical Centers” OR “Academic Medical Centers” OR “Academic Medical Center” OR “hospital” OR “Hospitals” OR “Hospital unit” OR “Hospital units” OR “Nursery” OR “Nurseries” OR “Residential Facilities” OR “Residential Facility” OR “Primary Healthcare” OR “Primary care” OR “Primary Health care”) ) OR AB ( ("Health Service” OR “Health Services” OR “University Medical Center” OR “University Medical Centers” OR “Academic Medical Centers” OR “Academic Medical Center” OR “hospital” OR “Hospitals” OR “Hospital unit” OR “Hospital units” OR “Nursery” OR “Nurseries” OR “Residential Facilities” OR “Residential Facility” OR “Primary Healthcare” OR “Primary care” OR “Primary Health care”))

#3 #1 OR #2

#4 MH(Palliative Care OR (Hospice and Palliative Nursing) OR Hospices OR Hospice Patients OR Hospice Care OR Terminal Care OR Terminally Ill Patients OR End of life)

#5 TI ("implementation science" OR "implementation research" OR "diffusion of innovation" OR "knowledge translation" OR "knowledge transfer" OR "knowledge exchange" OR "improvement science" OR implementation OR implementing) OR AB ("implementation science" OR "implementation research" OR "diffusion of innovation" OR "knowledge translation" OR "knowledge transfer" OR "knowledge exchange" OR "improvement science" OR implementation OR implementing)

#6 MH (diffusion of innovation OR medical practice, research based OR program evaluation OR quality improvement)

#7 #5 OR #6

#8 #3 AND #4 AND #7

#9 Filter: Peer Reviewed, English, January 1 2010 – December 31 2019

**Ageline (Ebsco)**

#1 DE "Health Services" OR DE "Dental Services" OR DE "Dietary Services" OR DE "Emergency Health Services" OR DE "Hospital Services" OR DE "Mental Health Services" OR DE "Outpatient Services" OR DE "Patient Care" OR DE "Pharmaceutical Services" OR DE "Physician Services" OR DE "Primary Care" OR DE "Public Health Services" OR DE "Health Service Facilities" OR DE "Clinics" OR DE "Hospitals" OR DE "Mental Health Centers" OR DE "Nursing Homes" OR DE "Skilled Nursing Facilities" OR DE "Board and Care Homes" OR DE "Boarding Homes" OR DE "Assisted Living Facilities”

#2 AB("Health Service” OR “Health Services” OR “University Medical Center” OR “University Medical Centers” OR “Academic Medical Centers” OR “Academic Medical Center” OR “hospital” OR “Hospitals” OR “Hospital unit” OR “Hospital units” OR “Nursery” OR “Nurseries” OR “Residential Facilities” OR “Residential Facility” OR “Primary Healthcare” OR “Primary care” OR “Primary Health care”)

#3 TI("Health Service” OR “Health Services” OR “University Medical Center” OR “University Medical Centers” OR “Academic Medical Centers” OR “Academic Medical Center” OR “hospital” OR “Hospitals” OR “Hospital unit” OR “Hospital units” OR “Nursery” OR “Nurseries” OR “Residential Facilities” OR “Residential Facility” OR “Primary Healthcare” OR “Primary care” OR “Primary Health care”)

#4 #1 OR #2 OR #3

#5 DE "Palliative Care" OR DE "Terminal Care" OR DE "Hospice"

#6 AB(Palliative Care OR (Hospice and Palliative Nursing) OR Hospices OR Hospice Patients OR Hospice Care OR Terminal Care OR Terminally Ill Patients OR End of life)

#7 TI(Palliative Care OR (Hospice and Palliative Nursing) OR Hospices OR Hospice Patients OR Hospice Care OR Terminal Care OR Terminally Ill Patients OR End of life)

#8 #5 OR #6 OR #7

#9 (DE "Quality Assurance") OR (DE "Program Evaluations")

#10 AB("implementation science" OR "implementation research" OR "diffusion of innovation" OR "knowledge translation" OR "knowledge transfer" OR "knowledge exchange" OR "improvement science" OR implementation OR implementing)

#11 TI("implementation science" OR "implementation research" OR "diffusion of innovation" OR "knowledge translation" OR "knowledge transfer" OR "knowledge exchange" OR "improvement science" OR implementation OR implementing)

#12 #9 OR #10 OR #11

#13 #4 AND #8 AND #12

#14 Filters: English, January 1 2010 – December 31 2019

**PsycInfo (ProQuest)**

#1 MAINSUBJECT.EXACT ("Hospitals") OR MAINSUBJECT.EXACT("Residential Care Institutions") OR MAINSUBJECT.EXACT("Primary Health Care") OR MAINSUBJECT.EXACT.EXPLODE("Health Care Services") OR MAINSUBJECT.EXACT("Long Term Care") OR MAINSUBJECT.EXACT("Home Care")

#2 ti (“Health Service” OR “Health Services” OR “University Medical Center” OR “University Medical Centers” OR “Academic Medical Centers” OR “Academic Medical Center” OR “hospital” OR “Hospitals” OR “Hospital unit” OR “Hospital units” OR “Nursery” OR “Nurseries” OR “Residential Facilities” OR “Residential Facility” OR “Primary Healthcare” OR “Primary care” OR “Primary Health care”) OR ab(“Health Service” OR “Health Services” OR “University Medical Center” OR “University Medical Centers” OR “Academic Medical Centers” OR “Academic Medical Center” OR “hospital” OR “Hospitals” OR “Hospital unit” OR “Hospital units” OR “Nursery” OR “Nurseries” OR “Residential Facilities” OR “Residential Facility” OR “Primary Healthcare” OR “Primary care” OR “Primary Health care”)

#3 #1 OR #2

#4 ti (("implementation science" OR "implementation research" OR "diffusion of innovation" OR diffusion of innovation OR "knowledge translation" OR "knowledge transfer" OR "knowledge exchange" OR "improvement science" OR "translational medical research" OR "program evaluation" OR "quality improvement" OR "implementation" OR "implementing")) OR ab(("implementation science" OR "implementation research" OR "diffusion of innovation" OR diffusion of innovation OR "knowledge translation" OR "knowledge transfer" OR "knowledge exchange" OR "improvement science" OR "Translational Medical Research" OR "Translational Research" OR "Translational Medicine" OR "program evaluation" OR "quality improvement" OR "implementation" OR "implementing"))

#5 MAINSUBJECT.EXACT("Innovation") OR MAINSUBJECT.EXACT("Knowledge Transfer") OR MAINSUBJECT.EXACT("Information Dissemination") OR MAINSUBJECT.EXACT("Program Evaluation") OR MAINSUBJECT.EXACT("Health Care Delivery") OR MAINSUBJECT.EXACT("Best Practices")

#6 #4 OR #5

#7 (MAINSUBJECT.EXACT.EXPLODE("Palliative Care") OR MAINSUBJECT.EXACT.EXPLODE("Hospice") OR MAINSUBJECT.EXACT.EXPLODE("Terminally Ill Patients") OR MAINSUBJECT.EXACT.EXPLODE("Treatment Withholding")) OR (AB("Palliative Care" OR hospice* OR "hospice care" OR "Terminally Ill Patients" OR "terminally ill" OR "End of life" OR "end-of-life" OR "end-of-life care" OR "Terminal Care" OR "palliative treatment" OR "palliative medicine" OR "comfort care" OR "supportive care" OR "withholding treatment" ) OR TI("Palliative Care" OR hospice* OR "hospice care" OR "Terminally Ill Patients" OR "terminally ill" OR "End of life" OR "end-of-life" OR "end- of-life care" OR "Terminal Care" OR "palliative treatment" OR "palliative medicine" OR "comfort care" OR "supportive care" OR "withholding treatment" ))

#8 #3 AND #6 AND #7

#9 Filter: Peer review, English, January 1 2010 – December 31 2019

Supplementary File 2. Overview of the included studies; authors, publication year, research approaches and designs, study aim, country, settings, participant category and numbers, data collection methods, implementation strategies and major findings (n=183)

| **Author, year Qual/quant/mixed methods Research design** | **Aim** | **Cou ntry Setting** | **Participants (Category & Number)  Data collection** | **Implementation**  **strategies** | **Major findings** |
| --- | --- | --- | --- | --- | --- |
| Albizu-Rivera et al (2016)  Quantitative  Unspecified design | The purpose of the current study was to assess implementation of key aspects of the PC Guidelines by NCCN member institutions. | USA  Other | Health care professionals. Unclear number  Surveys | Guidelines | Implementation of PC Guidelines is incomplete and various aspects of the guidelines, such as the recommendation to screen all patients for PC needs, are applied inconsistently. Despite this, most institutions provide PC services in a manner consistent with the guidelines. |
| Arenella, Finke et al (2010)  Quantitative  Unspecified design | In 2006, the Indian Health Service (IHS) and the National Cancer lnstitute (NCI) collaborated to develop an interdisciplinary palliative training program for health professionals in the Indian health system. Their goal was to improve clinician knowledge and skills in palliative care, to train future trainers, and to increase access to palliative care for American Indians and Alaska Natives. | USA  Unspecified | Nurses, physicians, social workers, pharmacists, other. 89 participants  Surveys | Educational materials  Conferences, courses, workshops | Evaluations demonstrated increased clinician self-reported knowledge and confidence to train and high satisfaction with training. Forty-two of 67 participants completed an anonymous post-conference Web questionnaire. Nearly half had conducted or definitively planned palliative education sessions, and 57 percent started new palliative services at their practice sites. |
| Arenella, Yox et al (2010)  Mixed methods  Unspecified design | This article describes an evaluation to assess the effectiveness of one effort, a pilot partnership with Medscape to disseminate a portion of the EPEC™-O curriculum. | USA  Unspecified | Nurses, physicians, 16 803 participants  Surveys | Educational materials  Conferences, courses, workshops | Satisfaction was very high among participants, and many indicated their intention to incorporate new knowledge into practice. |
| Armstrong et al (2013)  Quantitative  Quasi-experimental design | The objective of the Palliative Care Impact Study was to evaluate the impact of a PCCS in a rural Appalachian community hospital in 4 domains: clinical, customer, operational, and financial. | USA  Hospital, in-patient | Patients, 25 participants  Survey, existing register data, other | Consultations | Findings demonstrated a statistically significant improvement in pain and symptom burden scores; customer metrics produced an excellent rating of 88.5% by patients and families and physician/providers; with an associated cost savings of $521 per patient per day after PC consultation was complete. |
| Badger et al (2012)  Mixed methods  Unspecified design | To evaluate the impact of a training programme to improve end-of-life care in nursing homes, on collaboration between nursing home staff and other health practitioners. | United Kingdom  Residential care facility | Managers, nurses, residents, family, other. 75 participants  Surveys, intervies (individual and group) | Conferences, courses, workshops  Consultations, facilitators, support services or helplines | Improved collaborations as a result of the programme were anticipated by 31% of managers. Challenges to collaboration included working with large numbers of general practitioners, out-of-hours services and access to specialist practitioners. Improved collaborations between home staff and health service practitioners were identiﬁed by 33% of managers as one of the main programme outcomes. Staff reported increased knowledge of end-of-life care, and enhanced conﬁdence, which in turn resulted in improved communication and collaboration. Post-programme, staff felt more conﬁdent initiating contact and discussing residents’ end-of-life care with general practitioners and those working in specialist palliative care services. |
| Bailey et al (2014)  Quantitative  Experimental design | To evaluate the effectiveness of a multimodal intervention strategy to improve processes of end-of-life care in inpatient settings. | USA  Hospital, in-patient | Physicians, nurses, others. 7587 participants  Patient journal or record data | Educational materials Conferences, courses, workshops Reminders or checklists, triggers, templates Guidelines, toolkits, policies, tech tools | Significant intervention effects were observed for orders for opioid pain medication (OR: 1.39), antipsychotic medications (OR: 1.98), benzodiazepines (OR: 1.39), death rattle medications (OR: 2.77), sublingual administration (OR: 4.12), nasogastric tubes (OR: 0.71), and advance directives (OR: 1.47). Intervention effects were not significant for location of death, do-not-resuscitate orders, intravenous lines, or restraints. |
| Beck et al (2013)  Mixed methods  Quasi-experimental | The aim was to investigate the effects of an intervention that applies a palliative care approach in residential care upon nurse assistants’ level of strain, job satisfaction, and view of leadership. | Sweden  Residential care facilities | Physicians, nurses, managers. 82 participants  Surveys | Conferences, courses, workshops Multi-professional group meetings | Directly after the intervention, the job satisfaction of the nurse assistants decreased and they perceived the leadership more negatively than before the intervention. Six months later, strain as a result of criticism from residents and their superiors and having difﬁculty in balancing emotional involvement had decreased |
| Beck et al (2014)  Qualitative  Unspecified design | The aim was to describe the nurse assistants’ experience of how an intervention with a palliative care approach, had inﬂuenced them in their work in residential care for older people. | Sweden  Residential care facilities | Nurse assistants, 14 participants   Individual interviews | Conferences, courses, workshops | The nurse assistants felt that, through the intervention, they had gained insight into their understanding of the importance of quality of care. This included an increased awareness of, and respect for, residents’ and relatives’ needs, and an increased understanding of the importance of the outcome of encounters with residents and their relatives. After the intervention, they also felt there was increased openness and understanding between colleagues. However, the nurse assistants also expressed frustration over obstacles to implementing a palliative care approach, such as lack of resources and supportive leadership. |
| Beernaert et al (2017)  Quantitative  Experimental | We aimed to assess the effectiveness of the Care Programme for the Last Days of Life (CAREFuL) at improving comfort and quality of care in the dying phase in elderly people. | Belgium  Hospital, in- or out-patient | Patients, 312 participants  Surveys | Educational materials Conferences, courses, workshops Guidelines, toolkits, policies, tech tools | Implementation of CAREFuL compared with control significantly improved nurse-assessed comfort (CAD-EOLD baseline-adjusted mean difference 4·30, 95% CI 2·07–6·53; p<0·0001). No significant differences were noted for the CAD-EOLD assessed by family carers (baseline-adjusted mean difference –0·62, 95% CI –6·07 to 4·82; p=0·82) or the SM-EOLD assessed by nurses (–0·41, –1·86 to 1·05; p=0·58) or by family carers (–0·59, –3·75 to 2·57; p=0·71). |
| Bekelman et al (2014)  Mixed methods  Experimental | Our aim was to determine the feasibility and acceptability of CASA and identify necessary improvements. | USA  Hospital, in-patient and out-patient | Patients. 30 participants. Surveys, Individual interviews | Conferences, courses, workshops Educational outreach visits. Multi-professional group meetings. Consultations, facilitators, support services or helplines. | Participants were male with a median age of 63 years. One withdrew early and there were <5% missing data. Overall, 85% of 87 collaborative care team medical recommendations were implemented. All participants who screened positive for depression were either treated for depression or thought to not have a depressive disorder. In the qualitative interviews, patients reported a positive experience and provided several constructive critiques. |
| Bergman et al (2015)  Quantitative  Unspecified design | The study objective was to develop an online module about end-of-life care targeted at surgeons, and to assess the effect of the module on attitudes towards and knowledge about end-of-life care. | USA  Hospital, in-patient and out-patient | Health care professionals, Others. 114 participants  Surveys | Conferences, courses, workshops | Subjects improved meaningfully in all ﬁve domains of attitude and in each of the six knowledge items. Individuals younger than 30 years of age had the greatest change in attitudes about addressing pain, addressing end-of-life goals, and being actively involved as death approached; they also had the most marked improvement in total knowledge score. Having a family member die of cancer within the last ﬁve years or a personal experience with palliative care or hospice were associated with higher change scores. |
| Berkowitz et al (2011)  Quantitative.  Quasi-experimental | To evaluate an intervention to improve discharge disposition from a skilled nursing unit (SNU). | USA  Hospital, in-patient | Patients. Unclear number.  Observations, Existing register data | Conferences, courses, workshops Consultations, facilitators, support services or helplines. Reminders or triggers, check-lists, templates Guidelines, toolkits, policies, tech tools | Discharge dispositions were signiﬁcantly differently distributed across the two periods (P5.03). Readmission to acute care declined (from 16.5% to 13.3%, a nearly 20% decline). Multivariable logistic regression, controlling for age, sex, and case-mix index and adjusting for clustering due to repeated admissions of individual patients, suggests that, during the intervention period, patients were more likely than during the baseline period to die on the unit in accordance with their wishes than to be transferred out to the hospital (odds ratio52.45, 95% conﬁdence interval51.09–5.5). |
| Bernacki et al (2012)  Quantitative  Unspecified design | Our two well established palliative care programs in large academic hospitals used an innovative quality improvement initiative to broaden access to palliative care services, particularly to noncancer patients. | USA  University hospital Hospital, in- or out-patient | Physicians, Nurses, Patients, Others. Unclear number.  Patient journal or record data | Financial intervention Other | Consultation rates in the target populations tripled following the initiative: from 16% to 46% at one hospital and from 15% to 48% at the other. Although two different screening and identiﬁcation processes were developed, both successfully increased palliative care consultations in the target cohorts. |
| Beyea et al (2013)  Quantitative  Unspecified design | Our aim was to evaluate an intervention to improve communication about advance care planning (ACP) and symptom distress, and to facilitate referral to PC and hospice. | USA  Other | Nurses, Others. 510 participants  Surveys, existing register data, other | Conferences, courses, workshops Other dissemination strategies Multi-professional group meetings Other social interaction strategies Guidelines, toolkits, policies, tech tools Other | After training, CMs identiﬁed the following areas for expected practice change: ACP (29%), identifying/ referring patients for hospice or PC (25%), supporting patients and families (21%), toolkit utilization (10%), and engaging medical providers (10%). Over one-year follow-up the percent of moderate and high-risk ABD Medicaid patients asked about ACP or symptoms increased from 7% to 31% and 8% to 41%, respectively ( p < 0.001). The cumulative number of PC or hospice referrals increased from 8 to 155. Hospice enrolment at death was unchanged (29% to 30%, p =NS [nonsigniﬁcant]). |
| Blackwell et al (2017)  Mixed methods  Participatory/action research design | To critique the feasibility of this methodology as a quality improvement intervention in complex healthcare settings, laying a foundation for future work. | UK  Hospital, in- and out-patient | Health care professionals, Patients, Families. 103 participants  Interviews, observations, other | Other social interaction strategies | the study successfully identiﬁed quality improvement priorities leading to changes in Emergency Department-palliative care processes. Further outputs were the creation of a patient-family-staff experience training DVD to encourage reﬂective discussion and the identiﬁcation and application of generic design principles for improving palliative care in the Emergency Department. There were beneﬁts and challenges associated with using Experience-based Co-design in this setting. Beneﬁts included the ﬂexibility of the approach, the high levels of engagement and responsiveness of patients, families and staff, and the impact of using ﬁlmed narrative interviews to enhance the ‘voice’ of seldom heard patients and families. Challenges included high levels of staff turnover during the 19-month project, signiﬁcant time constraints in the Emergency Department and the ability of older patients and their families to fully participate in the co-design process. |
| Bökberg et al (2019)  Quantitative  Experimental | This study’s aim was to evaluate whether an educational intervention had any effect on the staff’s perception of providing person-centred palliative care for older persons in nursing homes. | Sweden | Nurses, Other healthcare professionals, 365. Surveys | Educational materials; Conferences, courses, workshops | Both the intervention group and the control group revealed high median scores in all subscales at baseline, except for the subscale amount of organizational and environmental support in the P-CAT. The staff’s high rating level of person-centred care before the intervention provides limited space for further improvements at follow-up |
| Bove et al (2018)   Qualitative  Other design | This study aimed to explore the health professionals’ expectations and experiences of a new palliative out-patients structure for patients with advanced COPD. | Denmark | Physicians, Nurses. 12 participants. Individual interviews; group interviews | Multi-professional group meetings; Consultations, facilitators, support services or helplines; Other decision-support strategies; Pathways or programmes | Nurses and physicians considered the new structure as a quality boost and it fulfilled their hope of improving the quality of care offered to patients with advanced COPD, however with increased work-related stress as a derived consequence |
| Bradley et al (2010)  Quantitative  Quasi-experimental | This study examines the practice within the SICU related to palliative care involvement before and after the initiative with the hypothesis that the triggers increased palliative care consultations. | USA | Other health care professionals, Patients. 644 participants. Patient journal or record data. | Reminders or triggers, check-lists, templates; | Results. Triggers were rare in both groups (Group I, 5.7%; Group II, 5.5%). Palliative care consultations were also infrequent, without change before and after the intervention (Group I, 2.3%; Group II, 3.1%). There was no difference in consultations for patients meeting a trigger after the initiative (17.6% to 27.3%; P = .704). |
| Bristowe & Carey (2018)  Qualitative  Other design | Aim: To explore healthcare professionals’ perceptions of using a complex intervention (AMBER care bundle) to improve care for people approaching the end of life and their understandings of its purpose within clinical practice. | UK (England) | Physicians, Nurses, Other healthcare professionals. 20 participants. Individual interviews. | Other social interaction strategies | Results: Three views emerged regarding the purpose of a complex intervention towards the end of life: labelling/categorising patients, tool to change care delivery and serving symbolic purpose indirectly affecting behaviours of individuals and teams. All impact upon potential utility of the intervention. Participants described the importance of training and education alongside implementation of the intervention. However, adequate exposure to the intervention was essential to witness its potential added value or embed it into practice. |
| Brousseau et al (2012)  Mixed methods  Unspecified design | This article describes the SPCPH’s distinctive design, features of the public hospital PCCS, patient and team characteristics, and PCCS provider perceptions of environmental factors, and SPCPH features that promoted or impeded their success. | USA | Physicians, Nurses, Other healthcare professionals, 7,368. Surveys; Other. | Conferences, courses, workshops; Educational outreach visits; Other dissemination or educational strategies; Consultations, facilitators, support services or helplines; Other social interaction strategies; Financial interventions | the Spreading Palliative Care in Public Hospitals initiative (SPCPH) has resulted in a 3-fold increase in the number of California public hospitals providing PCCS, from 4 to 12. |
| Brown & Ashcraft (2019)  Quantitative  Unspecified design | A problem existed in the initiation of palliative care for geriatric trauma patients because the palliative care team was uncomfortable taking care of surgical trauma patients and the trauma team was uncomfortable starting primary palliative care. By implementing a Geriatric Trauma Palliative Care Program (GTPCP) using the ACS guidelines, our trauma program took the first step to integrate evidence-based palliative care in a multidisciplinary trauma team. | USA | Patients. 188 participants. Patient journal or record data. | Educational materials; Conferences, courses, workshops; Other dissemination or educational strategies; Reminders or triggers, check-lists, templates; Pathways or programmes; Guidelines, toolkits, policies, tech tools; Other | Using Person’s χ 2  test and Fisher’s exact test, our initial evaluation of the program showed statistically significant (p < .001) improvements in the measures related to the implementation of primary palliative care, pain and symptom management, and end-of-life care. The guidelines gave the team a consistent framework for implementing the basic competencies required to deliver primary palliative care, pain and symptom management, and end-of-life care to trauma patients. |
| Brown-Saltzman et al (2010)  Quantitative  Unspecified design | We describe the one-day educational program and report its short-term effects on RTs. | USA | Other healthcare professionals. 85 participants. Surveys. | Conferences, courses, workshops | RESULTS: Nearly all the RTs had recently encountered end-of-life situations, yet most had not received dedicated training and felt ill-prepared to deal with these situations; one third reported distress related to withdrawal of treatment. The 78 participants who completed both the before and after surveys had increased comfort with end-of-life care (P < .001) and their perception of their role in end-of-life care (P < .001). Knowledge about end-of-life care also increased (P < .001). |
| Calvel et al (2019)   Qualitative  Descriptive/Explorative | Identified Palliative Care Beds (Lits Identifiés Soins Palliatifs – LISPs) is a French specificity. Primarily created to integrate palliative care culture into conventional hospital units, the relevance of this measure became a controversial issue. Nowadays, hospital teams continue to frequently encounter complex situations regarding medical care for palliative patients. To the best of our knowledge, there is only one study, a quantitative one, bridging the gap about that subject. It showed failure in practicing palliative care work around LISP. Our study is based on a qualitative method that complements the quantitative study. It aimed to describe difficulties that limit palliative care practices in managing adult patients in LISP. | France | Physicians, Nurses, Other healthcare professionals. 20 participants. Individual interviews; | Other | From a quantitative perspective, the interviews revealed 305 difficulties, indicating the gaps and barriers limiting the implementation of a palliative approach in these services. From a qualitative perspective, five topics raised our attention by their recurrence in discourses: (1) partial knowledge about palliative care definition and legislation mostly due to a lack of training; (2) need for time; (3) need for human resources; (4) need for communication; (5) hard time in transitioning from curative to palliative care. |
| Campion-Smith et al (2011)   Qualitative  Unspecified design | This article describes the evaluation of a series of six  monthly meetings aimed to enable professionals providing palliative care to learn with, from and about each other and, through improved knowledge, skill and conﬁdence, to provide better care. We sought a subjective response and reports of changed practice and improved outcomes for patients through analysis of participants’ narratives. | UK (England) | Unspecified health care professionals. 19 participants. Individual interviews. | Multi-professional group meetings | Respondents reported effects including changed behaviours and beneﬁt to patients. ... Five months after the end of the course, many participants described changed professional behaviour which they believed led to improved patient outcomes. |
| Carey et al (2015)  Quantitative  Unspecified design | We describe the design, development and implementation of an innovative care bundle, ‘the AMBER care bundle’, to improve the care for patients, in the acute hospital setting, who may be in the last 1–2 months of life and whose potential for recovery is uncertain | UK (England) | Patients. 638 participants Unspecified. | Other social interaction strategies | In total 42.8% died in hospital and a further 14.5% were readmitted as emergencies within 30 days of discharge. Clinical outcome measures are in development |
| Centeno et al (2017)  Mixed methods  Unspecified design | This study aims to identify barriers and opportunities to the  I-PC in European countries according to service provision levels. | Europe | Other. Unclear (48/53 countries); Surveys; Other. | Guidelines, toolkits, policies, tech tools; Other organisational strategies | Results: In total, 48/53 (91%) European countries responded to the survey. A total of 43 barriers and 65 opportunities were identiﬁed as being related to PC integration. Main barriers were (1) lack of basic PC training, with a particular emphasis on the absence of teaching at the undergraduate level; (2) lack of ofﬁcial certiﬁcation for professionals; (3) lack of coordination and continuity of care for users and providers; (4) lack of PC integration for noncancer patients; (5) absence of PC from countries’ regulatory frameworks; and (6) unequal laws or regulations pertaining to PC within countries. Innovations in education and new regulatory frameworks were identiﬁed as main opportunities in some European countries, in addition to opportunities around the implementation of PC in home care, nursing home settings, and the earlier integration of PC into patients’ continuum of care. With increasing provision of services, more challenges for the integration are detected ( p < 0.005). |
| Chan et al (2014)  Mixed methods  Quasi-experimental | Aims and objectives. To report on the effectiveness of an eight-week palliative care programme in Hong Kong. | Hong Kong | Patients, Families. 108 participants. Surveys; Individual interviews; | Conferences, courses, workshops; Mass media campaigns or other public campaigns; Other dissemination or educational strategies; Other social interaction strategies | Results. Pearson’s chi-square tests and Wilcoxon matched paired tests show a general trend that the patients’ quality of life was improved after the programme. Their understanding and active participation in advance care planning was also improved. The hospital readmission rate and the days of hospital stays were signiﬁcantly reduced. In qualitative interview, four major themes were identiﬁed that are as follows: improvement in the communication of treatment plans and after-death arrangements, symptom management, emotional support and suggested areas of improvement. |
| Childers & Arnold (2018)   Quantitative  Unspecified design | Our primary aim was to increase the frequency with which non-PC-trained clinicians conduct and document GOC conversations in seriously ill patients. We also wished to increase these clinicians’ conﬁdence in having these discussions and ascertain whether a large-scale communication training program could be carried out on a health system level. |  | Physicians. 512 participants. Surveys; Patient journal or record data | Educational materials; Conferences, courses, workshops; Other dissemination or educational strategies; Consultations, facilitators, support services or helplines | A large-scale educational intervention involving simulated patient cases increased GOC documentation across  a health system. Other programs might consider collaboration with quality improvement specialists to measure the impact of education and situate it within other system changes to support increased GOC discussions. |
| Chi-Yin et al (2014)  Quantitative  Quasi-experimental | Objectives: The purpose of this study was to evaluate the effects of the hospital-based palliative care team on the care for cancer patients. | Taiwan | Patients, 60 participants. Surveys | Multi-professional group meetings | Results: Comparison between groups revealed that the degree change for oedema, fatigue, dry mouth, abdominal distention, and spiritual well-being in the intervention group showed signiﬁcant improvement compared to the control group (p < 0.05). However, there was no difference between groups on measures of anxiety, depression and feeling of social support. Within group analysis showed patients’ pain score, dyspnoea, and dysphagia improved in both groups (p < 0.05). In addition, the average degree of constipation and insomnia in the control group declined from baseline (p < 0.05), while the degree of oedema, fatigue, dry mouth, appetite loss, abdominal distention, and dizziness decreased signiﬁcantly in the intervention group (p < 0.05) |
| Clark, Sheward, Marshall & Allan (2012)  Mixed methods  Unspecified design | We report on the post-implementation ﬁndings of a mixed methodology (survey and focus group [FG] forums) study into staff perceptions of EOL care following the pilot implementation of the LCP into two acute wards. | New Zealand | Physicians, Nurses, Other healthcare professionals, Unspecified healthcare professionals. 18 participants. Surveys; Group interviews | Pathways or programmes; Other | Results and conclusions: Study results suggest that within acute settings staff perceive that the LCP improves EOL care overall, assists interdisciplinary communication around death and dying, and that is a useful tool to positively inﬂuence decision making and care delivery. Further research into aspects of staff communication, diagnosing dying, changing direction of care, and the physical environment is warranted. |
| Clark, Marshall, Sheward & Allan (2012)  Mixed methods  Unspecified design | Aim: To determine staff perceptions of the impact of the Liverpool Care Pathway for the dying patient (LCP) in three aged residential care facilities in New Zealand. | New Zealand | Physicians, Nurses, Other healthcare professionals, Unspecified healthcare professionals. 15 participants. Surveys; Individual interviews; Group interviews | Pathways or programmes | The participating staff perceived multiple benefits to residents, family, and staff following implementation of the LCP. |
| Clark et al (2015)  Quantitative  Quasi-experimental | The aim of this study was to examine whether or not the quality initiative had any effect on the ward nurse’s attitudes and self-assessed competency to care for dying patients. | Australia | Nurses. 64 participants. Surveys | Other social interaction strategies; Reminders or triggers, check-lists, templates | Results: Over the 6 months the bundle was piloted, 74.5% of people who died did so with the bundle in place. While this was seen as clinically useful by nearly half the nurses who responded, there was not a significant change in the staff’s attitudes or self-assessed competency to care for dying patients. There was a minor change in the Thanatophobia Scale (pre 18.2: SD±9.0 versus post 16.8: SD 7.8; P=0.53), the Self-efficacy in Palliative Care Scale for communication (pre 47.4: SD ±17.4 versus post 54.7:SD±17.9; P=0.11) and patient management respectively (pre 54.3: SD ±12.9 versus 59.1: SD ±12.6; P=0.15). |
| Clark et al (2017)  Mixed methods  Observational | This work was conducted with the aim of exploring the feasibility of including bereaved relatives’ experiences as part of a larger project exploring the use of a care bundle to improve care of the dying inpatients. | Australia | Families. 20 participants. Individual interviews; Other | Other social interaction strategies; Reminders or triggers, check-lists, templates | No major differences in the prescores and postscores were noted. When invited to share their experiences, without prompting, families spoke of consistent concerns that included communication, place of death, and symptom control. |
| Collins et al (2016)  Mixed methods  Observational | Objectives To determine the views and experiences of health and social care professionals on using integrated care pathways (ICPs) for caring for people in the last days to hours of life. | UK | Physicians, Nurses, Other healthcare professionals, Unspecified healthcare professionals. 1331. Surveys | Other decision-support strategies | Results 1331 professionals returned completed questionnaires. Ninety-three per cent (1138/1228) of respondents used the Liverpool Care Pathway (LCP) or local variant. Eighty-eight (1089/1234) felt ICPs enabled professionals to provide better care for individuals and their families/carers. ICPs were viewed as promoting patient-centred holistic care, improving pain and symptom control, providing guidance and standards and improving communication with patients/families. Sixty-two per cent (770/1234) had no concerns regarding the use of ICPs. Areas of concern included incorrect use and implementation of the ICP, poor communication with families, junior level staff making decisions and insufficient education and support |
| Corcoran (2016)  Mixed methods  Unspecified design | The purpose of this project was to deliver and evaluate a workshop to professional caregivers that enabled attendees to meet the needs of patients, families, and themselves during the end-of-life (EOL) process. | USA | Nurses, Other healthcare professionals. 33 participants. Surveys | Conferences, courses, workshops | Results suggest the workshop was effective in improving nurses’ comfort levels with delivering EOL care. The program’s success provides a foundation for future education offerings related to EOL care. |
| Cornetta et al (2015)  Quantitative  Unspecified design | This study reviews the implementation of a palliative care service based at the Moi Teaching and Referral Hospital in Eldoret, Kenya, and describes the current scope and challenges of providing palliative care services in an East African tertiary public referral hospital. | Kenya | Patients. 1244 participants. Individual interviews; Other | Conferences, courses, workshops | Barriers to providing optimal palliative cancer care include distance to pharmacies that stock opioids, limited selection of opioid preparations, education of health care workers in palliative care, access to palliative chemoradiation, and limited availability of outpatient and inpatient hospice services. |
| Costantini, Romoli et al (2014)  Quantitative  Experimental | We tested  the hypothesis that outcomes for patients and families could be improved through procedural changes by the introduction of the LCP-I programme (the Liverpool Care Pathway programme translated and adapted to the Italian context). | Italy | Patients, Families, Unspecified healthcare professionals. Unclear number. Surveys; Individual interviews; Existing register data; Patient journal or record data | Conferences, courses, workshops; Other dissemination or educational strategies; Multiprofessional group meetings; Consultations; facilitators, support services or helplines; Other decision-support strategies; Guidelines, toolkits, policies, tech tools | Findings During the postintervention assessment, data were gathered for 308 patients who died from cancer (147 in LCP-I programme wards and 161 in control wards). 232 (75%) of 308 family members were interviewed, 119 (81%) of 147 with relatives cared for in the LCP-I wards (mean cluster size 14·9 [range eight to 22]) and 113 (70%) of 161 in the control wards (14·1 [eight to 22]). After implementation of the LCP-I programme, no signiﬁcant diﬀerence was noted in the distribution of the overall quality of care toolkit scores between the wards in which the LCP-I programme was implemented and the control wards (score 70·5 of 100 vs 63·0 of 100; cluster-adjusted mean diﬀerence 7·6 [95% CI –3·6 to 18·7]; p=0·186). |
| Costantini, Pellegrini et al (2014)  Quantitative  Quasi-experimental | Preliminary assessment of the effectiveness of the Liverpool Care Pathway on the quality of end-of-life care provided to adult cancer patients during their last week of life in hospital. | Italy | Patients, Families. 190 participants. Individual interviews | Multi-professional group meetings; Consultations, facilitators, support services or helplines; Other decision-support strategies | Following Italian version of Liverpool Care Pathway implementation, there was a significant improvement in the mean scores of four Toolkit scales: respect, kindness and dignity (+16.8; 95% confidence interval = 3.6–30.0; p = 0.015); family emotional support (+20.9; 95% confidence interval = 9.6–32.3; p < 0.001); family self-efficacy (+14.3; 95% confidence interval = 0.3–28.2; p = 0.049) and coordination of care (+14.3; 95% confidence interval = 4.2–24.3; p = 0.007). No significant improvement in symptom’ control was observed. |
| Cox et al (2017)   Mixed methods  Explorative | The aim of the study was to (1) Increase the confidence and competence of care home staff in EoLC; and (2) Enable more residents the opportunity to experience EoLC in their care home rather than an acute setting. | UK (England) | Patients, Other healthcare professionals. 78 participants. Surveys | Conferences, courses, workshops; Multi-professional group meetings; Guidelines, toolkits, policies, tech tools | The development, implementation and evaluation of a collaborative end-of-life care intervention for care homes. |
| Creutzfeldt et al (2015)  Quantitative  Unspecified design | Instead of identifying potential need for palliative care consultation through diagnosis-based or length-of-stay triggers, we aimed to determine the prevalence of unmet palliative care needs in our patient population and to explore the effect of a palliative care needs screening tool (PNST) on patient care and outcomes. | USA | Patients. 262 participants. Patient journal or record data; Other | Other social interaction strategies; Reminders or triggers, check-lists, templates | Palliative care needs were identified in 62% of screened patients (80/130). Needs were mainly social support (53%) and establishing goals of care (28%). Screening was associated with more documented family conferences (p=0.019) and a trend towards more palliative care consultations (p=0.056). |
| Cronfalk et al (2015)  Qualitative  Unspecified design | Our objective was to describe nursing home staff’s attitudes to competence-building programs in palliative care. | Sweden | Nurses, Other healthcare professionals, Unspecified healthcare professionals. 852 participants. Group interviews | Conferences, course workshops, Other decision-support strategies | The results suggest that staff reported positive experiences as they gained new knowledge and insight into palliative care. The experiences seemed to be similar independent of the educational program design. Our results also show that staff experienced difﬁculties in talking about death. Enrolled nurses and care assistants felt that they carried out advanced care without the necessary theoretical and practical knowledge. Further, the results also suggest that lack of support fromward managers and insufﬁcient collaboration and of a common language between different professions caused tension in situations involved in caring for dying people. |
| Cross et al (2012)  Qualitative  Unspecified design | This paper aims to capture the learning from the project, focusing on how successful and constructive were each of these elements. It will be of particular interest to commissioners and providers of health and social care services for people with ID, and to both general and specialist palliative care services; those concerned with developing and implementing end-of-life care strategy locally and nationally; and key training providers working with care staff and professionals in both ID and palliative care services | UK (England) | Unspecified healthcare professionals, Other. Unclear number. Individual interviews; Other | Conferences, courses, workshops; Other dissemination or educational strategies; Other social interaction strategies | In general, the study found that there was a lack of understanding of each other’s role between palliative care professionals and ID staff, with each unsure of what the other service is providing and how it is run. |
| Curtis et al (2011)  Quantitative  Experimental | We evaluated the effectiveness of a quality-improvement intervention to improve intensive care unit (ICU) end-of-life care. | USA | Nurses, Patients, Families. 2238 participants. Surveys; Existing register data; Patient journal or record data | Conferences, courses, workshops; Other dissemination or educational strategies; Feedback to stakeholders; Other social interaction strategies; Guidelines, toolkits, policies, tech tools; Other | The primary outcome, family-QODD, showed no change with the intervention (P 5 0.33). There was no change in family satisfaction (P5 0.66) or nurse-QODD (P5 0.81). There was a nonsigniﬁcant increase in ICU days before death after the intervention (hazard ratio 5 0.9; P5 0.07). Among patients undergoing withdrawal of mechanical ventilation, there was no change in time from admission to withdrawal (hazard ratio 5 1.0; P5 0.81). |
| DeMiglio et al (2012)  Qualitative  Unspecified design | To assist in developing and sustaining interdisciplinary PC teams working in a shared care mode … explored the challenges and barriers that teams encountered in Ontario, Canada. | Canada | Other healthcare professionals. Unclear number. Group interviews | Multiprofessional group meetings; Consultations, facilitators, support services or helplines | Teams circumvent local level barriers through four enabling factors: team characteristics, geography, adaptation of practice, and relationship building. Understanding these factors and strategies to foster them will assist other jurisdictions wanting to establish a similar shared care service delivery model. |
| Di Leo et al (2011)  Qualitative  Other design | This study is aimed at exploring the expectations about and the impact on healthcare staff of the Liverpool Care Pathway for the dying patient (LCP) in an Italian hospital. | Italy | Physicians, Nurses. 13 participants. Group interviews | Conferences, courses, workshops; Educational outreach visits; Consultations, facilitators, support services or helplines; Other decision-support strategies | Five major topics were identified: managing pain and discontinuing inappropriate treatments, communicating with patients, communicating with relatives, communicating between professionals and practical issues. As compared with those reported in the initial FGs, responses from the final FGs highlighted that physicians felt more confident with pain management and with discontinuing inappropriate treatment, and were more inclined to recognize the value of the nurses’ work. Nurses underlined advantages in using pro re nata medication, but stressed lack of personnel and time as obstacles in consistent improvement of end-of-life care. All participants seemed to acquire greater awareness of their difficulties in communication and, paradoxically, became more uncertain of their ability to liaise with dying patients and their families. |
| Di Leo et al (2015)  Qualitative  Other design | To explore the views of professionals who, during the hospital implementation of the Italian version of the Liverpool Care of the Dying Pathway (LCP-I), voiced or showed concerns towards it. | Italy | Physicians, Nurses. 11 participants. Individual interviews | Conferences, courses, workshops; Multi-professional group meetings; Consultations, facilitators, support services or helplines; | A total of 12 categories were identified, referring to four topics: the Implementation Programme, the LCP-I clinical documentation, the hospital environment and the educational and professional background of hospital healthcare staff. Issues raised by participants concerned both ‘real’ characteristics of the LCP-I and a misinterpretation of the LCP-I approach and clinical documentation. Furthermore, difficulties were reported which were not linked to the Programme but rather to end-of-life care. |
| DiMartino et al (2017)   Mixed methods  Unspecified design | . A promising approach to improve integration is a triggered palliative care consultation (TPCC). This study evaluated the impact of two TPCC approaches on consistency and quality of consult implementation, operationalized as uptake and timeliness, on solid tumour medical and gynaecologic oncology services at an academic hospital. | USA | Physicians, Unspecified healthcare professionals, Patients. 9760. Existing register data; Unspecified | Conferences, courses, workshops; Educational outreach visits; Consultations, facilitators, support services or helplines; Reminders or triggers, check-lists, templates | Overall, 8.8% of medical oncology and 11.0% of gynaecologic oncology inpatient encounters involved palliative care consultation. In regression analyses, TPCC supported by a single strategy in gynaecologic oncology was associated with greater uptake vs. usual care (aRR: 1.45, p<.05), and TPCC supported by multiple strategies in medical oncology was associated with greater uptake vs. a single strategy (aRR: 2.34, p<.001). |
| DiMartino et al (2018)  Quantitative  Case study | We used the Organizational Theory of Innovation Implementation to further understand the role of formal and informal implementation policies and practices as determinants of implementation effectiveness. We examined their role within the context of initiatives to increase palliative care consultation in inpatient oncology. | USA | Unspecified healthcare professionals, Patients. 26 participants. Individual interviews; Existing register data | Conferences, courses, workshops; Educational outreach visits; Multi-professional group meetings; Consultations, facilitators, support services or helplines; Other decision-support strategies; Guidelines, toolkits, policies, tech tools | Medical oncology employed multiple formal policies and practices including training and clinician prompting to support palliative care consultation and a top-down approach, yet most clinicians were unaware of the policies and practices, contributing to a weak implementation climate. In contrast, gynaecologic oncology employed one formal policy (written guideline of criteria for initiating a consult) but also relied on informal policies and practices, such as spontaneous feedback and communication; they adopted a bottom-up approach, contributing to broader clinician awareness and strong implementation climate. Both services exhibited variable, increasing consult rates over time. |
| Downing et al (2016)  Mixed methods  Unspecified design | The aim of the study was to evaluate the impact of the palliative care link nurse programme at Mulago Hospital | Uganda | Nurses, Unspecified healthcare professionals. 27 participants. Surveys; Individual interviews; Group interviews; Other | Educational outreach visits | A significant difference was seen in nurses’ confidence after the training (p < 0.001). From July 2012 to December 2013, link nurses identified 2447 patients needing PC, of whom they cared for 2113 (86 %) and referred 334 (14 %) to MPCU. Clinical guidelines/protocols were utilised in 50 % of wards. Main themes identified include: change in attitude; developing new skills and knowledge; change in relationships; improved outcomes of care, along with the challenges that they experienced in integrating PC. Since the start of the programme there has been an increase in PC patients seen at the hospital (611 in 2011 to 1788 in 2013). |
| Ellis-Smith et al (2018)  Mixed methods  Other design | We aimed to explore the mechanisms of action, feasibility, acceptability and implementation requirements of a measure, the Integrated Palliative care Outcome Scale (IPOS-Dem), used in routine care to support comprehensive assessment of symptoms and concerns of care home residents with dementia and their family members. | UK | Physicians, Nurses, Families, Other. 26 participants. Surveys; Individual interviews; Group interviews; Observations. | Guidelines, toolkits, policies, tech tools | Key mechanisms of action were: improved observation and awareness of residents, collaborative assessment, comprehensive ‘picture of the person’, systematic record keeping, improved review and monitoring, care planning and changes to care provision, and facilitated multi-agency communication. Potential benefit included improved symptom management, improved comprehensive care, and increased family empowerment and engagement. IPOS-Dem was found to be acceptable and feasible. It was perceived as quick and easy to use, with proportion of overall missing data decreasing from 2.1% to 1.1% from baseline to final time points. ‘Trust’ in the measure was important; and leadership essential to ensure integration into care processes. |
| Ersek et al (2010)  Mixed methods  Unspecified design | This paper reports the development and evaluation of an interdisciplinary, international palliative care workshop presented in Gaborone, Botswana. | Botswana | Physicians, Nurses, Other healthcare professionals, Other. 47 participants. Surveys. | Conferences, courses, workshops | Evaluation indicated high satisfaction with the workshop and signiﬁcant, though modest, gains in knowledge and self-evaluation of palliative care skills. Discussion revealed important clinical issues for attendees and underscored the need to coordinate national efforts to enhance palliative care in Botswana. |
| Ersek et al (2018)  Qualitative  Unspecified design | We conducted an implementation-focused project evaluation to describe stakeholders’ perspectives on (a) the most and least effective components of the intervention; (b) barriers to implementation; and (c) program features that promoted its adoption. | USA | Other healthcare professionals, Unspecified healthcare professionals, Families. 63 participants. Individual interviews; Group interviews; | Other | We found universal endorsement of the value of in-depth advance care planning (ACP) discussions in reducing hospitalizations and improving care. Similarly, all stakeholder groups emphasized that nursing home access to specially trained, project registered nurses (RNs) and nurse practitioners (NPs) with time to focus on ACP, comprehensive resident assessment, and staff education was particularly valuable in identifying residents’ goals for care. Challenges to implementation included inadequately trained facility staff and resistance to changing practice. In addition, the program sometimes failed to communicate its goals and activities clearly, leaving facilities uncertain about the OPTIMISTIC clinical staff’s roles in the facilities. |
| Evans et al (2019)   Mixed methods  Unspecified design | The overall goals of INTEGRATE were to (a) enhance provider knowledge and confidence in palliative care delivery, (b) identify patients with cancer who might benefit from palliative care earlier in their disease trajectory, and (c) increase the provision of palliative care and use of palliative care tools. This study assesses the extent to which these goals were met, thereby providing insight into feasibility, stakeholder experiences, and early impact of the project | Canada | Physicians, Nurses, Other healthcare professionals. 28 participants. Surveys; Individual interviews; Group interviews; Existing register data | Conferences, courses, workshops; Consultations, facilitators, support services or helplines; Reminders or triggers, check-lists, templates; Other decision-support strategies; Pathways or programmes | A total of 760 patients with cancer (lung, glioblastoma, head and neck, gastrointestinal) were included. Results suggest improvement in provider confidence to deliver palliative care and to initiate the Advanced Care Planning (ACP) conversation. The majority of patients (85%) had an ACP or goals of care (GOC) conversation initiated within a mean time to conversation of 5‐46 days (SD 20‐93) across centres. A primary care report was transmitted to family physicians 48‐100% of the time within a mean time to transmission of 7‐54 days (SD 9‐27) across centres. Enablers and barriers influencing success of the model were also identified |
| Fedel et al (2014)  Quantitative  Quasi-experimental | The purpose of this research study was to examine whether an educational intervention and implementation of a validated prognostication tool can improve inpatient acute care nurses’ knowledge of palliative care and their comfort in determining the need for palliative care and requesting a palliative care consult from the attending physician. | USA | Nurses. 39 participants. Surveys | Conferences, courses, workshops; Consultations, facilitators, support services or helplines; Other decision-support strategies | Pre- and post-test comparisons showed an overall increase in both comfort and knowledge related to palliative care. There was also a signiﬁcant improvement in the nurses’ comfort in identifying patients appropriate for palliative care. |
| Fernandes et al (2014)   Unclear  Quasi-experimental | To develop and test a culturally appropriate palliative care navigation curriculum for the RMI. | USA | Unspecified healthcare professionals, Other. 24 participants. Surveys; Group interviews; Other | Conferences, courses, workshops; Other social interaction strategies | Collaboration between a US Academic Institution and International  Ministry of Health to develop a culturally appropriate palliative care navigation curriculum. |
| Finkelstein et al (2016)  Quantitative  Unspecified design | We aimed to determine the ability of these triggers to identify patients who would beneﬁt from palliative care consultation. | USA | Patients. 492 participants. Existing register data; Other | Reminders or triggers, check-lists, templates | Factors signiﬁcantly associated with hospital death or hospice discharge were repeat SICU admission, metastatic/advanced cancer, SICU physician referral, and the matching of 2 or more secondary criteria. |
| Finucane et al (2013)   Quantitative  Unspecified design | To sustain a high standard of palliative care in seven UK nursing care homes using a lower level of support than employed during the original project and to evaluate the effectiveness of this intervention. | UK (Scotland) | Patients. 132 participants. Other | Conferences, courses, workshops; Educational outreach visits; Consultations, facilitators, support services or helplines; Other social interaction strategies; Other decision-support strategies; Pathways or programmes; | During the sustainability project, 132 residents died. In comparison with the initial intervention, there were increases in (a) the proportion of deceased residents with an anticipatory care plan in place (b) the proportion of those with Do Not Attempt Cardiopulmonary Resuscitation documentation in place and (c) the proportion of those who were on the Liverpool Care Pathway when they died. Furthermore, there was a reduction in inappropriate hospital deaths of frail and elderly residents with dementia. However, overall hospital deaths increased. |
| Frank et al (2012)  Quantitative  Quality Improvement | The purpose of this study was to pilot-test and evaluate the impact  and feasibility of a nursing-led quality improvement intervention using the CANHELP questionnaire with or without systematic symptom screening, to identify clinical areas for improvement in care for patients with a high risk of death admitted to acute-care medical units. | Canada | Patients, Families. 80 participants. Surveys; Other | Conferences, courses, workshops; Multi-professional group meetings; Other decision-support strategies | The average satisfaction of priority items improved. Also, caregiver satisfaction improved signiﬁcantly and ESAS scores improved. Using CANHELP, the nurse facilitator was able to identify opportunities for improving EoL care in patients on medical units and for making small improvements in satisfaction with care. |
| Frendak et al (2019)   Quantitative  Quasi-experimental | To describe the implementation of a new palliative care triage process and to demonstrate its impact on efficiency, teamwork, and patient care. | USA | Unspecified healthcare professionals, Patients. Unclear. Existing register data | Other dissemination or educational strategies; Multi-professional group meetings; Other organizational strategies | Across the 2 study periods, consult demand increased by 44% while the physician staffing (full time equivalent [FTE]) decreased by 38%. Penetration rate per clinical FTE increased (from 1.9%-2.4%; P ¼ .004). Monthly physician work relative value units (RVUs) per FTE increased from 909 to 1678. Physician encounters with hospitalized patients increased from 284 to 353, and total team visits increased from 596 to 891 (P < .001). Average time to consult decreased by 2.4 hours (P ¼ .54). |
| Friedrichsen et al (2017)  Quantitative  Quasi-experimental | This study aimed to study whether a PCCT can influence and change primary healthcare team members’ perceptions regarding the palliative care at the end of life they are providing to patients in their own acute wards. | Sweden | Physicians, Nurses, Other healthcare professionals. 252 participants. Surveys | Other dissemination or educational strategies | A total of 252 team members (pre-post-intervention n =132/n = 120) participated in the study. Overall, 11 of the 12 statements scored significantly higher after the intervention than before. Responses varied significantly between different professions and depending on the number of dying patients cared for during the last month. The five with the highest Wald values were as follows: the presence of a break point dialogue with a patient, where the changed aim and focus of care was discussed; early detection of impending death; adequate symptom relief and psychological and existential issues. |
| Gaertner et al (2010)  Quantitative  Unspecified design | To evaluate whether this adoption of the WHO recommendation sufﬁced to integrate PC into routine cancer care early in the course of the illness, a retrospective analysis of the ﬁrst PC consultation provided for lung cancer (LC) patients was performed. The aim of the evaluation was to assess at what point in the disease trajectory integration of PC could be achieved. | Germany | Patients. 131 participants. Existing register data; Other | Multi-professional group meetings; Pathways or programmes | Most patients were already in a reduced physical state, were experiencing burdening symptoms and many died shortly after the ﬁrst PC consultation. After a one-year period, the number of burdening symptoms identiﬁed at ﬁrst PC consultation and the admissions to the in-patient PC was decreased while non-PC physicians increasingly requested PC support for psychosocial interventions. |
| Gilbert et al (2012)  Mixed methods  Unspecified design | This article provides an overview of the PPCIP QI innovation including its organization and its impact on improvement aims and care processes, assessed using both quantitative and qualitative approaches. | Canada | Patients. Unclear number. Surveys; Individual interviews; Group interviews; Other | Other decision-support strategies; Other | Within one year of implementation, regional cancer centres saw  improvements in symptom screening (54% of lung cancer patients), symptom control (69% of patients with pain scores and 31% of patients with dyspnoea scores seven or more were reduced to six or less within 72 hours), and functional assessment (23% of all patients and 64% of palliative care clinic patients). ESAS screening rates reached29%,and functional assessment reached26%oftargetedhomecarepatients. |
| Gillet & Bryan (2016)   Mixed methods  Unspecified design | This article describes the national rollout and evaluation of the 'Quality End of Life Care for All' (QELCA) programme funded by the NHS National End of Life Care Programme. | UK | Nurses, Other. 44 participants. Surveys; Group interviews | Educational materials; Conferences, courses, workshops; Other dissemination or educational strategies; Multi-professional group meetings; Consultations, facilitators, support services or helplines; Other decision-support strategies; Pathways or programmes | Participants gave concrete examples of improvements they made to end-of-life care and the majority of those who returned post-course questionnaires believed that QELCA had changed their practice. Additional positive outcomes included better working relationships between acute trusts and hospices, and more appropriate referrals. |
| Glajchen et al (2011)  Quantitative  Unspecified design | A Rapid Two-Stage Screening Protocol for Palliative Care in the Emergency Department: A Quality Improvement Initiative | USA | Patients. 1587 participants. Individual interviews; Patient journal or record data; Other | Conferences, courses, workshops; Educational outreach visits; Consultations, facilitators, support services or helplines; Other social interaction strategies; Other decision-support strategies | 1587 patients were screened, representing 22%  of ED visits made by patients older than 65 years during this time period. Of these, 140 met functional decline criteria, and 51 of these needed palliative care consultations. Five patients were referred to hospice, 20 received palliative care, and 26 received no further service. |
| Glare et al (2013)   Quantitative  Quality improvement | The goal of this pilot project was to evaluate the feasibility of implementing the screening and referral components of the NCCN Guidelines for Palliative Care in patients admitted to the Gastrointestinal Oncology Service (GIOS) at a comprehensive cancer centre (CCC). |  | Nurses, Patients. 16; 194 participants. Surveys; Existing register data | Reminders or triggers, checklists, templates; Guidelines, toolkits, policies, tech tools; Other organizational strategies | . During the study period, 229 (90%) total admissions were screened, with 169 (73%) having positive results. Of the Team A admissions, 72 (64%) met the referral criteria. More consults occurred for patients in Team A {47 vs 15; P=.001). In 30% of the referral criteria-triggered consults, the PC needs were manageable by the primary team. Nurses reported screening to be easy and quick {<5 minutes per patient) but only somewhat helpful. Being unfamiliar with many patients and families, floor nurses often felt unable to screen them accurately for some issues. In conclusion, screening was feasible, increasing access to PC, but accuracy and usefulness are concerns. With a consult indicated in 64% patients, yet with 30% being manageable by the primary team, the current criteria may be too sensitive for the inpatient environment of a CCC. |
| Golden et al (2016)  Quantitative  Descriptive/Explorative | This article describes the creation of an interprofessional  palliative care service to provide person-centred support in the homes of Veterans with advanced diseases. A retrospective descriptive review was conducted to measure the clinical and psychosocial interventions utilized by the consult service to address the complex issues of Veterans with advanced illnesses. The challenges developing such a home-based team are also discussed. | USA | Patients. 73 participants. Other; Unspecified | Conferences, courses, workshops; Mass media campaigns or other public campaigns; Multi-professional group meetings; Consultations, facilitators, support services or helplines; | The most common interventions of the consult team included discussion of advance directives, completion of a ‘‘do not resuscitate’’ form, reduction/stoppage of at least 1 medication, explanation of diagnosis, referral to home-based primary care program, referral to hospice, and assessment/support for caregiver stress. The home-based consult service was therefore able to address clinical and psychosocial issues that can demonstrate a direct benefit to Veterans, families, and referring clinicians. |
| Gradwohl et al (2019)  Quantitative  Quality improvement | The Preventing Readmissions through Effective Partnerships—Communication and Palliative Care (PREP-CPC) intervention was designed to increase the frequency of GOC conversations for hospitalized patients facing serious illness. | USA | Physicians, Nurses, Other healthcare professionals, Other. Unclear number. Surveys; Other | Conferences, courses, workshops; Multi-professional group meetings; Consultations, facilitators, support services or helplines; Other social interaction strategies; Reminders or triggers, check-lists, templates; | Over the 3-year study period, 134 clinicians from29hospital teams were trained to facilitate GOC conversations. After the kick-off conference, participants reported improvements in their confidence in facilitating GOC conversations. The hospital teams then instituted site-specific pilot interventions to promote GOC conversations, identifying essential elements required for ongoing improvement. Since projects varied by hospital, results did as well, but reported positive outcomes included increased GOC conversations, increased Practitioner Orders for Life-Sustaining Treatment form completion rates, new screening and documentation methods, and increased support from leadership. |
| Grainger et al (2010)   Quantitative  Unspecified design | We developed a brief communication skills workshop to assist OHP with these conversations, and examined satisfaction with the workshop and perceived conﬁdence regarding these discussions. | Australia | Physicians, Nurses, Other healthcare professionals, Unspecified healthcare professionals. 62 participants. Surveys | Conferences, courses, workshops; Educational outreach visits | Sixty-two OHP participated in workshops. Overall, participants were highly satisﬁed  with the workshop content and format. All participants felt the workshop provided relevant practical information, and .80% thought that participation beneﬁted their work. Over 98% said that the workshop had increased conﬁdence in their communication skills. |
| Grant et al (2017)   Mixed methods  Unspecified design | This paper sets out to provide evidence of impact through a large trial of the integration of palliative care within the health systems of Kenya, Uganda, Rwanda and Zambia. | Kenya, Rwanda, Uganda, Zambia | Unspecified healthcare professionals, Patients, Other. Unclear number. Surveys; Individual interviews; Group interviews; Other | Conferences, courses, workshops; Other organizational strategies; Other | Palliative care was integrated into all 12 hospital settings to various degrees through concurrent interventions of these four approaches. Overall, 218 advocacy activities were undertaken and 4153 community members attended awareness training. 781 staff were equipped with the skills and resources to cascade palliative care through their hospitals and into the community. Patients identified for palliative care increased by a factor of 2.7. All 12 hospitals had oral morphine available and consumption increased by a factor of 2.4 over two years. Twenty–two UK mentors contributed 750 volunteer days to support colleagues in each hospital transfer knowledge and skills. |
| Hahne et al (2017)  Qualitative  Other | The aim of this study is to evaluate how the implementation of palliative care, using a combination of integration and consultation strategies, can change beliefs regarding palliative care among professionals in a surgical department. | Sweden | Nurses, physicians. 7 participants. Group interviews | Conferences, courses, workshops; Educational materials; Consultations, facilitators, support services or helplines | Beliefs regarding palliative care were identified in seven areas; the importance of palliative care, working methods in palliative care, team collaboration in palliative care, collegial support, discussions about diagnosis, symptoms at the end of life, and families of patients in palliative care. Changes in beliefs were seen in all areas except one: team collaboration in palliative care. |
| Hall K et al (2016)  Qualitative  Unspecified design | To explore barriers to the design, implementation and delivery of a hospital-based palliative care program, this study used qualitative research methods to identify, describe, and analyse the scope of hospital-based palliative care programs in the Western New York region as of 2013. | USA | Physicians, Nurses, Other healthcare professionals. 9 participants. Individual interviews; Group interviews | Pathways or programmes | The findings identify challenges facing both existing/evolving palliative care programs, and establish a foundation for strategies to attain best practices not yet implemented. This study affirms the growing availability of palliative care services among these selected hospitals along with opportunities to improve the scope of services in line with national recommendations. |
| Hall S et al (2011)   Qualitative  Unspecified design | The aim of this study is to explore the perceived benefits of, and barriers to, implementation of the Gold Standards Framework for Care Homes (GSFCH), a quality improvement programme in palliative care | UK | Nurses, Other healthcare professionals, Patients, Families. 44 participants. Individual interviews | Pathways or programmes | Perceived benefits of the GSFCH included: improved symptom control and team communication; finding helpful external support and expertise; increasing staff confidence; fostering residents’ choice; and boosting the reputation of the home. Perceived barriers included: increased paperwork; lack of knowledge and understanding of end-of-life care; costs; and gaining the cooperation of GPs. Many of the tools and tasks in the GSFCH focus on improving communication. Participants described effective communication within the homes, and with external providers such as general practitioners and specialists in palliative care. However, many had experienced problems with general practitioners. Although staff described the benefits of supportive care registers, coding predicted stage of illness and advance care planning, which included improved communication, some felt the need for more experience of using these, and there were concerns about discussing death. |
| Hanson, Collichio, et al (2017)  Mixed methods  Quality improvement | To increase goals-of-care (GOC) communication for hospitalized patients with Stage IV cancer. | USA | Physicians, Nurses, Patients, Other. 330 participants. Surveys; Patient journal or record data; Other | Other dissemination or educational strategies; Reminders or triggers, check-lists, templates | In the 11-month study period, n=330, Stage IV cancer patients were hospitalized. Comparing the ﬁrst three months with the ﬁnal three months, rates of GOC discussion increased from 29% to 48% ( p=0.013), and specialty palliative care consultation increased from 18% to 33%, (p= 0.026). Rates of symptom screening, intensive care unit transfer, hospice, and 30-day re-admission did not change overall. However, patients with specialty palliative care more frequently had pain screening (91% vs. 81%, p=0.020), spiritual assessment (48% vs. 10%, p<0.001), and hospice referral (39% vs. 9%, p<0.001), and they were less likely to be re-admitted within 30 days (12% vs. 21%, p=0.059). |
| Hanson, Zimmerman et al (2017)  Quantitative  Experimental design | To test a goals of care (GOC) decision aid intervention to improve quality of communication and palliative care for nursing home residents with advanced dementia. | USA | Patients, Families. Unclear number. Individual Interviews; Patient journal or record data | Educational materials; Conferences, courses, workshops; Multi-professional group meetings; Other decision-support strategies | Residents’ mean age was 86.5 years, 39 (12.9%) were African American, and 246 (81.5%) were women. With the GOC intervention, family decision makers reported better quality of communication (QOC, 6.0 vs 5.6; P = .05) and better end-of-life communication (QOC end-of-life subscale, 3.7 vs 3.0; P = .02). Goal concordance did not differ at 3 months, but family decision makers with the intervention reported greater concordance by 9 months or death (133 [88.4%] vs 108 [71.2%], P = .001). Family ratings of treatment consistent with preferences, symptom management, and quality of care did not differ. Residents in the intervention group had more palliative care content in treatment plans (5.6 vs 4.7, P = .02), MOST order sets (35%vs 16%, P = .05), and half as many hospital transfers (0.078 vs 0.163 per 90 person-days; RR, 0.47; 95% CI, 0.26-0.88). Survival at 9 months was unaffected (adjusted hazard ratio [aHR], 0.76; 95% CI, 0.54-1.08; P = .13). |
| Harding et al (2013)   Quantitative  Quasi-experimental | This study aimed to evaluate, in terms of patient outcomes, palliative care delivered by the existing HIV outpatient clinical personnel in Tanzania, compared to standard HIV outpatient care. | Tanzania | Nurses, Patients. 60; 68 participants. Surveys | Conferences, courses, workshops; Educational outreach visits; Other dissemination or educational strategies; Multi-professional group meetings; | For the primary pain outcome, the required sample size of 120 patients was recruited. Odds of reporting pain reduced significantly more at intervention site (OR00.60, 95% CI 0.50~0.72) than at control (OR00.85, 95% CI 0.80~0.90), p00.001. For secondary outcomes, longitudinal analysis revealed significant difference in slope between intervention and control, respectively: Medical Outcomes Study-HIV (MOS-HIV) physical score 1.46 vs. 0.54, p00.002; MOS-HIV mental health 1.13 vs. 0.26, p00.006; and POS total score 0.84 vs. 0.18, p00.001. Neither baseline CD4 nor antiretroviral therapy (ART) use was associated with outcome scores. These data are the first to report outcomes evaluating integrated HIV outpatient palliative care in the presence of ART. The data offer substantive evidence to underpin the existing WHO clinical guidance that states an essential role for palliative care alongside HIV treatment, regardless of prognosis. |
| Hauser et al (2015)  Mixed methods  Unspecified design | Our aim was to describe the development of EPEC adaptations and document the dissemination of our curriculum. | USA, Korea, India, Saudi Arabia, Canada, Turkey, Honduras, Argentina, Mexico, Guatemala, | Other healthcare professionals, Unspecified healthcare professionals. 554 participants. Surveys; Other | Educational materials; Other dissemination or educational strategies; Reminders or triggers, check-lists, templates | In its second 5 years of active development, teaching, and dissemination, we have created ﬁve major adaptations (EPEC-Oncology, EPEC-Oncology-Canada, EPEC-Emergency Medicine, EPEC-India, and EPEC for Veterans) and trained more than 1000 trainers. Through the efforts of these Trainers and our online dissemination, more than 74,000 reported end-learners have been taught parts of the EPEC curriculum. In addition, we discovered multiple medical school courses, continuing medical education (CME), courses and specialty guidelines that have incorporated material from EPEC. |
| Haydar et al (2017)  Quantitative  Observational | To evaluate (1) providers’ perceptions regarding the feasibility of SQ use in emergency and inpatient settings, (2) clinician perceptions regarding the utility of the SQ, and (3) barriers to SQ use. | USA | Physicians, Nurses, Other healthcare professionals. 111 participants. Surveys | Other decision-support strategies | A total of 111/203 (55%) providers participated: 48/57 (84%) emergency physicians (EPs) and 63/146 (43%) inpatient providers (IPs). Most reported no difﬁculty using the SQ. Modest numbers in both groups reported that the SQ inﬂuenced care delivery (EPs 37%, IPs 42%) as well as goals of care (EPs 45%, IPs 52%). At least some advance care planning discussions were prompted by the SQ (EPs 45%, IPs 58%). Team discussions were inﬂuenced by SQ use for more than half of each group. Most respondents (55%) expressed some concern that their SQ responses could be inaccurate. |
| Head et al (2010)   Mixed methods  Unspecified design | The purpose of this pilot project was to integrate palliative care principles and practices into the day-to-day operations of a Medicaid managed care provider. | USA | Physicians, Other healthcare professionals. 15 participants. Surveys; Group interviews | Educational materials; Conferences, courses, workshops; Consultations, facilitators, support services or helplines; Reminders or triggers, check-lists, templates; Other decision-support strategies; Guidelines, toolkits, policies, tech tools; Other | Expert staff was hired and modelled effective PCCM. This, as well as the training program, had significant influence on both the palliative care knowledge and attitudes of existing case managers. Involved patients demonstrated improved symptom management and satisfaction with care. Patient scenarios demonstrated desirable outcomes in healthcare utilization, and timely, appropriate hospice referrals were realized. |
| Herce et al (2014)  Mixed methods  Other design | We conducted a situation analysis to evaluate early NPCP outcomes and better understand palliative care needs, knowledge, and preferences. | Malawi | Patients, Families, Other. 74 participants. Individual interviews; Patient journal or record data | Other dissemination or educational strategies; Consultations, facilitators, support services or helplines; Pathways or programmes | The NPCP enrolled 63 patients in its first 9 months. Frequent diagnoses were cancer (n = 50, 79%) and HIV/AIDS (n = 37 of 61, 61%). Nearly all (n = 31, 84%) patients with HIV/AIDS were on antiretroviral therapy. Providers registered 112 patient encounters, including 22 (20%) home visits. Most (n = 43, 68%) patients had documented pain at baseline, of whom 23 (53%) were treated with morphine. A majority (n = 35, 56%) had $1 follow-up encounter. Mean African Palliative Outcome Scale pain score decreased non-significantly between baseline and follow-up (3.0 vs. 2.7, p= 0.5) for patients with baseline pain and complete pain assessment documentation. Providers referred 48 (76%) patients for psychosocial services, including community health worker support, socioeconomic assistance, or both. We interviewed 36 patients referred to the NPCP after the chart review period. Most had cancer (n = 19, 53%) or HIV/AIDS (n = 10, 28%). Patients frequently reported needing income (n = 24, 67%) or food (n = 22, 61%). Stakeholders cited a need to make integrated palliative care widely available. |
| Higginson et al (2013)  Mixed methods  Unspecified design | To adapt or develop and  evaluate a new evidence-based tool to better meet patient and family communication and holistic care needs in ICU. | UK (England) | Physicians, Nurses, Families. 260 participants. Surveys; Individual interviews | Other dissemination or educational strategies; Guidelines, toolkits, policies, tech tools; Other | PACE provides individualized assessments of all patients entering the ICU. It is completed within 24 to 48 hours of admission, and covers five aspects (key relationships, social details and needs, patient preferences, communication and information status, and other concerns), followed by recording of an ongoing communication evaluation. Implementation is supported by a training program with specialist palliative care. A post-implementation survey of 95 ICU staff found that 89% rated PACE assessment as very or generally useful. Of 213 family members, 165 (78%) responded to their survey, and two-thirds had PACE completed. Those for whom PACE was completed reported significantly higher satisfaction with symptom control, and the honesty and consistency of information from staff (Mann–Whitney U-test ranged from 616 to 1247, P-values ranged from 0.041 to 0.010) compared with those who did not. |
| Ho et al (2016)  Qualitative  Unspecified design | To critically examine the underpinnings of palliative long-term care provision from the perspectives of multi-stakeholders involved in the program, as well as, to identify the interplaying dynamics, mechanisms, and systemic factors that underscore the implementation practice of EoL-ICP in the Chinese context of Hong Kong. | China | Physicians, Unspecified healthcare professionals, Families, Others. 30 participants. Other | Pathways or programmes | Framework analysis revealed 10 themes, organized into 3 categories, namely, (1) Regulatory Empowerment (interdisciplinary teamwork, resource allocation, culture building, collaborative policy making), (2) Family-Centred Care (continuity of care, family care conference, partnership in care), and (3) Collective Compassion (devotion in care, empathic understanding, compassionate actions). |
| Hockley (2014)  Qualitative  Participatory/action research | This article reports on data collected from ten reﬂective debrieﬁng groups following the deaths of the residents in two nursing homes that were part of a larger action research study | UK | Other healthcare professionals, Unspecified healthcare professionals. 34 participants. Surveys; Individual interviews; Observations; Other | Consultations, facilitators, support services or helplines | Ten reﬂective debrieﬁng groups, led by the researcher (a specialist palliative care nurse), were undertaken. The groups facilitated learning at three different levels (being taught, developing understanding and critical thinking) and enabled staff to feel supported and valued. |
| Hockley & Kinley (2016)  Quantitative  Unspecified design | This paper describes one such initiative and reflects on the practice development model developed. | UK | Patients. Unclear number. Patient journal or record data | Conferences, courses, workshops; Pathways or programmes; Other | The percentage of residents dying in nursing care homes increased from 57% to 79%, with improvement  in other outcomes. |
| Hockley et al (2010)   Quantitative  Unspecified design | This paper is part of a larger project reporting the  impact of implementing both end-of-life care tools together using the same facilitator while proactively visiting the nursing homes two to three times a month using a model of empowerment. The extent to which the goals of the GSF are met are explored. | UK (Scotland) | Nurses, Other healthcare professionals, Patients. 288 participants. Surveys; Existing register data | Conferences, courses, workshops; Reminders or triggers, check-lists, templates; Pathways or programmes; Guidelines, toolkits, policies, tech tools | There was a highly statistically significant increase in use of Do Not Attempt Resuscitation (DNAR) documentation, advance care planning and use of the LCP. An apparent reduction in unnecessary hospital admissions and a reduction in hospital deaths from 15% deaths pre-study to 8% deaths poststudy were also found. |
| Holdsworth (2019)  Qualitative  Other design | To meet the multidimensional needs of patients, health services are increasingly implementing complex programmes of care through partnerships between public, private and voluntary sector organisations. The purpose of this paper is to explore the implementation process of a complex, multi-innovative regional health and social care partnership to coordinate end-of-life care in the South East of England. | UK | Other healthcare professionals, Other. 17 participants. Individual interviews; Group interviews; Observations; Other | Multi-professional group meetings; Consultations, facilitators, support services or helplines; Computerized decision support; Other decision-support strategies; Pathways or programmes; Guidelines, toolkits, policies, tech tools; Other organizational strategies | While progress was made towards greater collaboration in the provision of end-of-life care, regional coordination of care among the 13 partner organisations was not achieved as envisioned. Low engagement stemming from national health system changes delayed decision making and shifted partners’ priorities. Individual stakeholder interest and motivation carried the elements that were successful. |
| Hopkins et al (2017)  Mixed methods  Unspecified design | The primary objective of this study was to determine whether a supportive care plan program facilitates effective integration of palliative care and haematology. The second objective was to address cancer centres’ vision for improved communication and decision making for patients with incurable disease. |  | Nurses, Other healthcare professionals, Patients. 26 participants. Surveys | Multi-professional group meetings; Consultations, facilitators, support services or helplines; Other decision-support strategies; Pathways or programmes; Other | Seventy-seven percent of patients understood palliative care to be the primary team managing their symptoms, with 75% of patients viewing symptom control as the main goal of treatment. Staff findings demonstrated a significant improvement in the communication of treatment goals (53% pre-implementation vs 86% post-implementation). Early timing of referrals remains a significant issue |
| Horey et al (2012)   Mixed methods  Participatory/action research design | To investigate the acceptability and feasibility of using end-of-life (EOL) care pathways in residential aged care facilities (RACFs). | Australia | Physicians, Nurses, Other healthcare professionals, Patients. 70 participants. Surveys; Individual interviews; Other | Pathways or programmes | The use of EOL care pathways across the RACFs fell into low-, moderate- and high-uptake groups (for 10%, 34% and 68% of all deaths at the facility, respectively). Feedback from RACF staff and GPs indicated that acceptability was critical to successful implementation. The use of EOL care pathways demonstrated improvements in care, sometimes over extended periods. There were fewer unnecessary admissions to hospital before death, although not all RACF staff and GPs were aware of the project. |
| Hsu-Kim et al (2015)  Quantitative  Quality Improvement | As part of an ongoing quality improvement project to identify factors associated with quality and utilization of this service, we retrospectively evaluated the association between HPM, our palliative care consultation (PCC) service, and a number of clinical and economic outcomes of patients admitted to our medical and neurologic critical care services. We hypothesized that significant differences in clinical and economic outcomes exist between patients admitted to the ICU who received PCC and those who did not. | USA | Patients. 121 participants. Patient journal or record data; Other. | Multi-professional group meetings; Consultations, facilitators, support services or helplines | Patients in the PCC group were older (average 64 years, standard deviation [SD] 19.2 vs 55.6 years, SD 14.5; P ¼ .021) and sicker (median Acute Physiology and Chronic Health Evaluation IV score 85.5, interquartile range [IQR] 60.5-107.5 vs 60, IQR 39.2-74.75; P < .001) than the non-PCC controls. PCC patients received significantly more total days of ICU care on average (8 days, IQR 4-15 vs 4 days, IQR 2-7; P < .001), had more ICU admissions, and were more likely to die during their ICU stay (64.3% vs 12.5%, P < .001). Median total hospital charges per patient attributable to ICU care were higher in the PCC group than in the controls (US$315,493, IQR US$156,470-US$486,740 vs US$116,934, IQR US$54,750-US$288,660; P < .001). After we adjusted for ICU length of stay, we found that median ICU charges per day per patient did not differ significantly between the groups (US$37,463, IQR US$27,429-US$56,230 vs US$41,332, IQR US$30,149-US$63,288; P ¼ .884). Median time to PCC during the ICU stay was 7 days (IQR 2-14.5 days). |
| Hudson et al (2017)  Mixed methods  Unspecified design | We aimed to design and evaluate a prognostic screening tool to routinely identify inpatients with decompensated cirrhosis at high risk of dying over the coming year, alongside the development of a supportive care intervention | UK | Patients. 73 participants. Other. | Guidelines, toolkits, policies, tech tools | 73 admissions were scrutinised (79.5% male, 63% alcohol-related liver disease, median age 54). The presence of three or more poor prognosis criteria at admission predicted 1-year mortality with sensitivity, specificity and positive predictive value of 72.2%, 83.8% and 81.3%, respectively, and was used as a trigger for implementing the supportive care intervention. Following modification from six PDSA cycles, prognostic screening was integrated into the assessment of all patients admitted with  decompensated cirrhosis, with the supportive care intervention (developed simultaneously) instigated for appropriate patients. |
| Hurst et al (2018)  Quantitative  Quasi-experimental | We sought to explore the efficacy of using a simple, objec-  tive palliative care screening tool within the first 24 hours of ICU admission as a proactive means for increasing the frequency and timeliness of PCC. | USA | Unspecified healthcare professionals, Patients. 223 participants. Existing register data | Guidelines, toolkits, policies, tech tools | A total of 223 MICU admissions were evaluated: 156 patients in the control group and 67 patients in the intervention group. More consults were generated in the intervention group (22.39%) compared to the control group (7.05%; P < .001). The median time to consultation was lower in the intervention group compared to the control group (1 day vs 2 days; P < .01). |
| Hussainy et al (2010)  Quantitative  Unspecified design | The aim of the program was to address pharmacists’ education needs in this area, thereby improving their knowledge and conﬁdence. Because cancer is the mostcommon reason for palliation in Australia,5 the program speciﬁcally dealt with palliative cancer care. | Australia | Other healthcare professionals. 34 participants. Surveys | Educational materials; Other dissemination or educational strategies; Multi-professional group meetings | A program that pharmacists could access at a time and place convenient to them via the Internet was developed. Pharmacists indicated the program positively impacted their practice. |
| Hydeman (2013)  Quantitative  Unspecified design | The project’s goal was to increase the appropriateness of referrals to palliative care by the primary services in the hospital. The aim was to achieve this by developing a process to assess patient functioning in critical psychosocial and functional areas, and providing this data to referring medical staff to educate them on the contribution of palliative care to symptom control and patient quality of life. |  | Patients. 165 participants. Surveys | Other dissemination or educational strategies; Other decision-support strategies; Guidelines, toolkits, policies, tech tools | The findings show that referrals to palliative care have increased over 100% from a broader range of services since initiating this project. |
| Iliffe et al (2016)   Quantitative  Quasi-experimental design | The IMPACT project (IMplementation of Quality Indicators in PAlliative Care sTudy, 2011 to 2015) aimed to evaluate the potential of QIs as tools to improve palliative care for people with cancer or dementia in five European sites (England, Germany, Italy, Norway, and the Netherlands). We adopted Batalden and Davidoff’s definition of quality improvement [25] as “combined and unceasing efforts… to make changes that will lead to better patient outcomes (health), better system performance (care) and better professional development (learning)”. | UK (England) | Unspecified healthcare professionals, Other. Unclear number. Individual interviews; Observations; Other | Other dissemination or educational strategies; Multi-professional group meetings; Consultations, facilitators, support services or helplines; Feedback to stakeholders; Other decision-support strategies; Other | General practices could not be recruited to the study. Care homes were recruited but not retained. Hospital wards were recruited and retained, and using the Quality Indicator (QI) set achieved some of their desired changes. Hospices and community palliative care teams were able to use the QI set to achieve almost all their desired changes, and develop plans for quality improvements. Improvements included: increasing the utility of electronic medical records, writing a manual for end-of-life care, establishing working relationships with a hospice; standardising information transfer between settings, holding regular multi-disciplinary team meetings, exploration of family carers’ views and experiences; developing referral criteria, and improvement of information transfer at patient discharge to home or to hospital. Realist evaluation suggested that: 1) uptake and use of QIs are determined by organisational orientation towards continuous improvement; 2) the perceived value of a QI package was not powerful enough for GPs and care homes to commit to or sustain involvement; 3) the QI set may have been to narrow in focus, or more specialist than generalist; and 4) the greater the settings’ ‘top-down’ engagement with this change project, the more problematic was its implementation. |
| Imura et al (2014)  Qualitative  Unspecified design | The primary aim of this study was to explore  how and why a regional palliative care program led to changes as a part of a regionwide intervention study. | Japan | Physicians, Nurses, Other healthcare professionals. 101 participants. Individual interviews | Educational outreach visits; Other dissemination or educational strategies; Multi-professional group meetings; Feedback to stakeholders; Financial interventions; Other organizational strategies; Other | Seven themes were identiﬁed as follows: 1) improved communication  and cooperation among regional health care professionals; 2) increased conﬁdence in the system to care for cancer patients at home; 3) improved knowledge/skills, practice, and perception of palliative care; 4) contribution to self-growth; 5) wide variability in perceived changes in the knowledge and perception of patients, family members, and the general public; 6) wide variability in the perceived regionwide effects of the project; and 7) unresolved issues. |
| Jack et al (2011)  Qualitative   Unspecified design | The aim of the study was to evaluate the impact of the  palliative care Community Volunteer Programme. | Uganda | Unspecified healthcare professionals, Patients, Other. 64 participants. Group interviews | Conferences, courses, workshops; Feedback to stakeholders; Pathways or programmes | The results reported the value of the Community Volunteer Programme, including the impact on patients and families, and how the CVWs acted as a ‘bridge to the hospice’ in identifying patients. Developing financial challenges that are emerging which could potentially impact on the programme were reported. The Community Volunteer Programme appears to be having a positive impact on patients, families and the hospice team, and is a model worthy of consideration by other developing countries to allow the expansion of palliative care. |
| Jenko et al (2015)  Mixed methods  Quasi-experimental design | Aim 3: Evaluate the number of ICU palliative care referrals before and after implementation  & Aim 4: Evaluate the number of days between ICU admission and palliative referrals Aim 5: Explore the nurses’ perceptions of use of the PPSv2 as a palliative screening tool | USA | Nurses, Other healthcare professionals. 26 participants. Surveys; Observations; Existing register data; Other | Educational materials; Conferences, courses, workshops; Reminders or triggers, check-lists, templates | Over 610 observations, the rate of uptake increased over time and use of the scale ranged from 24.2% to 85.6%. The nurses’ (n = 26) comfort with palliative care issues increased from preintervention to postintervention, albeit not significantly. Knowledge items did not change. There was a 110% increase in the number of palliative care referrals between preintervention and postintervention and a nearly 1-day decrease in the number of days between medical ICU admission and palliative care referral; this reduction was not statistically significant. A majority of nurses (n = 22 [84.5%]) voted to retain the PPSv2 as an official process of care, stating that the tool facilitated assessment of patient needs that might have been previously overlooked. |
| Kadlec et al (2015)  Quantitative  Unspecified design | In this report, we present the findings from the evaluation surveys of the GPs who participated in the EOL learning module. We report on two sets of outcomes for the specific learning objectives of the EOL module’s faculty and content developers: (1) the GPs’ satisfaction with the module, including their perceptions of whether the module had met its objectives and its impact on their practice and their end-of-life patients, and (2) a number of EOL-specific outcomes and their changes across time, including GPs’ self-rated knowledge, communication skills, collaborations with other care providers, and conducting home visits. | Canada | Physicians. Unclear number. Surveys | Educational materials; Other dissemination or educational strategies; Consultations, facilitators, support services or helplines | Satisfaction and impact were rated very highly by over 90 % of the GP respondents. Module participation increased the GPs’ confidence on EOL-related communication and collaboration skills: e.g., initiating conversations about EOL care, developing an action plan for EOL care, communicating the patient’s needs and wishes to other care providers, participating in collaborative care with home and community care nurses, and accessing and referring patients to EOL specialists in the community. Increased confidence was maintained at 3-6 months following completion of training. |
| Ka-Ming Ho (2016)  Quantitative  Observational | To evaluate the obstacles and supportive behaviours to providing end-of-life care as perceived by emergency nurses after implementing the end-of-life care pathway. | Hong Kong | Nurses. 42 participants. Surveys | Pathways or programmes | Six of the 10 obstacles with the highest scores were related to family issues. Moreover, ﬁve of the 10 supportive behaviours with the highest scores were associated with the provision of favourable environment. Conclusion: Though emergency nurses in different parts of the world face similar obstacles and supportive behaviours, discrepancies exist because of differing care pathways, emergency department design, and nursing education |
| Karim et al (2018)   Quantitative  Quality improvement | We sought to improve the rate of documentation of GOC and referral to PC through the implementation of a quality improvement (QI) initiative | Canada | Patients. 303 participants. Existing register data; Patient journal or record data | Other dissemination or educational strategies; Consultations, facilitators, support services or helplines; Reminders or triggers, check-lists, templates; Computerized decision-support; Other decision-support strategies; Guidelines, toolkits, policies, tech tools | Between May 2016 and November 2017, a total of 303 unique patients were identiﬁed (52%, 21%, 17%, and 10% with lung, breast, colorectal, and pancreatic cancer, respectively). GOC documentation increased signiﬁcantly over the study period (baseline, 0%; passive phase, 3%; active phase, 31%); this increase was likely because of our intervention. PC referral rates also increased over the study period (baseline, 36%; passive phase, 35%; active phase 48%). We did not identify any patient, physician, or disease factors that were associated with GOC discussion or referral to PC. |
| Karlekar et al (2017)  Quantitative  Quality improvement | The purpose of our study was twofold. First, we aimed at determining the feasibility of incorporating a validated screening tool into the daily workﬂow of bedside clinicians to assess for both cognitive impairment and physical frailty among OAs admitted to a trauma unit. Second, we aimed at tracking and reporting our screening results and at determining whether the screening process could serve as an objective trigger for PC consultations and lead to an increase in overall referrals to PC earlier in patients’ clinical trajectories. | USA | Nurses, Patients. 131 participants. Surveys; Other | Conferences, courses, workshops; Other social interaction strategies | During the three-month period, the mean age of all older admissions (N= 131) was 75.5, of which 49% were screened. Among the patients screened, 38% screened positive for frailty, 45% screened positive for possible dementia, and 23% screened positive for both conditions. Palliative care consultations for older adults increased from 13% (before study) to 33% during the study period |
| Kelly et al (2011)  Quantitative  Unspecified design | This article describes a Californian programme to educate nurses and unlicensed staff in providing end-of-life care for older adults in nursing homes, skilled nursing facilities, long-term care facilities, and hospices. It looks at the development of the programme, its implementation, participants’ follow-up evaluations, and their self-reported practical outcomes. | USA | Nurses, Other. 308 participants. Surveys; Group interviews | Conferences, courses, workshops; Educational outreach visits; | This paper describes programme development, implementation, follow-up evaluations, and examples of participants’ use of the ELNEC Geriatric curriculum. |
| Kim et al (2012)  Mixed methods  Unspecified design | The aim of this study was to evaluate nurses’ satisfaction and knowledge following the attendance at the ELNEC‐Geriatric curriculum on nurses’ knowledge of palliative care. | Korea | Nurses. 128 participants. Surveys; Other | Conferences, courses, workshops; Educational outreach visits | Approximately eight nine percent of the nurses reported previous experience in caring for dying patients and attending various hospice palliative care training programs. Overall program satisfaction of the participants was 4.03 on a 5‐point scale, and their mean of the total PCQN score was 12.75 out of 20 after participating in ELNEC‐Geriatric course, which was a significant  improvement (p=.022) from the pre-test. |
| Kinley, Denton et al (2018)   Mixed methods  Unspecified design | To develop and implement a palliative care programme that would meet the needs of people with learning disabilities, their families and care staff. | UK | Other healthcare professionals. Unclear number. Unspecified | Conferences, courses, workshops; Consultations, facilitators, support services; Pathways or programmes | 39 homes were recruited and 86% completed the programme. |
| Kinley, Preston, et al (2018)   Mixed methods  Unspecified design | To identify the type, role, impact and cost of facilitation when implementing the GSFCH programme into NCH practice. | UK | Other healthcare professionals, Other. 17 participants. Surveys; Individual interviews; Other | Other dissemination or educational strategies; Multi-professional group meetings; Consultations, facilitators, support services or helplines; Pathways or programmes | Three facilitation approaches were provided to nursing home staﬀ when implementing the GSFCH programme: ‘ﬁtting it in’ facilitation; ‘as requested’ facilitation; and ‘being present’ facilitation. ‘Being present’ facilitation most eﬀectively enabled the completion of the programme, through to accreditation. However, it was not suﬃcient to just be present. Without mastery and commitment, from all participants, including the external facilitator, learning and initiation of change failed to occur. Implementation of the programme required an external facilitator who could mediate multi-layered learning at an individual, organisational and appreciative system level. The cost savings in the study outweighed the cost of providing a ‘being present’ approach to facilitation. |
| Kinley et al (2014)  Quantitative  Experimental design | It was hypothesised that action learning alongside high facilitation when implementing the Gold Standards Framework for Care Homes programme will result in a reduced proportion of hospital deaths for residents and improvement in the care home staff ability to facilitate good end-of-life care. | UK | Patients. 2 444 participants. Existing register data; Patient journal or record data.; Observations; | Conferences, courses, workshops; Consultations, facilitators, support services or helplines; Pathways or programmes | A greater proportion of residents died in those nursing homes receiving high facilitation and action learning but not significantly so. There was a significant association between the level of facilitation and nursing homes completing the Gold Standards Framework for Care Homes programme through to accreditation. Year-on-year change occurred across all outcome measures. |
| Kinley et al (2017)  Mixed methods  Unspecified design | To implement an end-of-life care programme, namely the 'Steps to Success' programme, in residential care homes. Measurable outcomes were collected through audit. ... The aim was not only to improve their delivery of care to residents but to be able to evidence that they had done so. |  | Other healthcare professionals, Patients. Unclear number. Patient journal or record data | Conferences, courses, workshops; Multi-professional group meetings; Consultations, facilitators, support services or helplines; Other social interaction strategies; Reminders or triggers, check-lists, templates; Pathways or programmes; Guidelines, toolkits, policies, tech tools; Other | Over four years audit of all deceased residents' records in the participating homes was collected. This shows an increase of home deaths in 2011/12 to 2014/15 from 44% (n=8/18) within four residential care homes to 64% (n=74/115) in 23 residential care homes with corresponding increase in advance care plan discussions and completion of 'do not attempt cardiopulmonary resuscitation' forms. |
| Kluger et al (2018)  Mixed methods  Quality improvement | In this manuscript we will describe some of the successes and challenges of this clinical program, including our current clinic operations and lessons from the initiation of the clinic, as well as supplemental information from two programs at other institutions. We also provide data from a one-year retrospective review of this clinic and a formal quality improvement project with the hopes of facilitating the implementation of similar clinics. We conclude with suggestions for further research and educational initiatives. | USA | Physicians, Nurses, Unspecified healthcare professionals, Patients, Families. 135 participants. Surveys; Individual interviews; Existing register data; Patient journal or record data; Other | Pathways or programmes | High satisfaction ratings from patients, caregivers, and referring providers. To maintain standardized and efficient care we have developed materials for patients and referring physicians as well as checklists and other processes used by our interdisciplinary team. Results from our chart review suggest our clinic influences advance care planning and place of death. Common referral reasons include psychosocial support, complex symptom management, and advance care planning. Current challenges for our clinic include developing a strategy for continued growth, creating a sustainable financial model for interdisciplinary care, integrating our services with disease-specific sections, improving primary palliative care knowledge and skills within our referral base, and building effective alliances with community neurologists, geriatrics, primary care, nursing homes, and hospices. |
| Koper et al (2019)   Quantitative  Unspecified design | Thus, in order to facilitate further implementation of  PaTz, this study first aims to compare PaTz-participants’ perceptions of the added value of PaTz and barriers for participating in PaTz with non-participants’ perceptions. As the roles of GPs and DNs in PaTz-groups differ, and the added value and barriers for participation may be different for GPs and DNs [16, 17], this study aims to compare the perceptions of participants and non-participants separately for each professional group. Second, this study aims to examine the association between PaTz-participation and care outcomes, by comparing the care provided to patients of GPs and DNs participating in a PaTz-group with the care provided to patients of GPs and DNs who are not. | The Netherlands | Physicians, Nurses. 327 participants. Surveys | Multi-professional group meetings | While both PaTz-participants and non-participants perceived PaTz to be beneficial for knowledge collaboration, coordination and continuity of care, time (or lack thereof) is considered the most important barrier for participation. PaTz-participation is associated with discussing five or more end-of-life topics with patients (OR = 3.16) and with another healthcare provider (OR = 2.55). PaTz-participation is also associated with discussing palliative sedation (OR = 3.85) and euthanasia (OR = 2.97) with another healthcare provider. Significant associations with other care outcomes were not found. |
| Kortes-Miller et al (2016)  Mixed methods  Unspecified design | This article aims to describe the development, implementation, and evaluation of a pilot educational intervention utilizing HFS to improve unregulated care providers’ confidence and skills communicating about death and dying in LTC homes. | Canada | Other healthcare professionals, Unspecified healthcare professionals. 18 participants. Surveys; Individual interviews; Observations; Other | Conferences, courses, workshops; Other dissemination or educational strategies; Multi-professional group meetings; Other | Results supported the effectiveness of HFS as an educational tool for unregulated health care providers. Quantitative data showed statistically significant improvements in participants’ self-efficacy scores related to communicating about death and dying and end-of-life care. Qualitative data indicated that the experience was a valuable learning opportunity and helped participants develop insights into their own values, beliefs, and fears providing end-of-life care. HFS is therefore recommended as an innovative training strategy to improve palliative care communication in long-term care homes. |
| Kramer et al (2014)  Mixed methods  Case study | The purpose of this study was to assess the feasibility of a ‘‘consultation  model,’’ whereby a hospice nurse (RN) and social worker (SW) were available to provide palliative care consultation to three CWPP teams for a 10-month period. This pilot study sought to address four primary study aims: (a) determine the extent to and ways in which consultants will be used by the teams; (b) identify the potential benefits of consultation from the perspective of the CWPP staff; (c) examine the extent to which consultation will assist CWPP team members to enhance their perceived success in the provision of palliative care; and (d) identify implementation challenges or barriers associated with the consultation model | USA | Nurses, Other healthcare professionals. 40 participants. Surveys; Group interviews; Observations | Consultations, facilitators, support services or helplines | Consults occurred primarily during team meetings and also informally and on joint patient visits and were primarily with the palliative care nurse addressing physical issues. Fifty-seven percent of consultant recommendations were implemented. Benefits of consultation were identified with focus groups of clinical staff as were opportunities and barriers to the implementation. Models of integration are proposed. |
| Krumm et al (2014)  Qualitative  Case study | To describe health professionals’ experiences of assessing the symptoms of people with dementia using a cancer-patient-oriented symptom-assessment tool from a palliative care context. |  | Unspecified healthcare professionals. 13 participants. Individual interviews | Guidelines, toolkits, policies, tech tools | Baseline interviews showed specific concerns about symptom assessment, such as uncertainty about underlying symptoms in residents who appeared to be in distress. After the implementation of the MIDOS tool, participants reported that daily use of the tool was perceived as helpful in evaluating symptoms other than pain and improved internal communication between staff regarding clinical decision making. |
| Kuhn & Forrest (2012)   Quantitative  Unspecified design | This article describes a pilot project involving training, case consultations, and administrative coaching over a period of 1 year aimed at introducing palliative care in 2 nursing homes among 31 residents with advanced dementia. | USA | Unspecified healthcare professionals, Patients, Families. 111 participants. Surveys; Patient journal or record data | Educational materials; Conferences, courses, workshops; Consultations, facilitators, support services or helplines; Other social interaction strategies | Limited improvements were demonstrated on measures for residents, staff members, and family members at the first nursing home (site 1) and significant improvements were demonstrated at the other nursing home (site 2). Top leadership turned over 3 times at site 1 which limited the integration of palliative care, whereas leadership of site 2 remained stable. |
| Kuo et al (2019)   Quantitative  Unspecified design | The study aims to compare the association of the PC policy with end-of-life care between patients with dementia and patients with cancer during the last 6 months of their lives | Taiwan | Patients. 31 715 participants. Existing register data | Pathways or programmes; Other | Among PDC, while the percentage of receiving PC increased from 3.6% in 1999 to 14.2% by the end of 2000 (adjusted OR (aOR)=4.07, 95% CI 2.70 to 6.13) and from 20.9% in 2010 to 41.0% in 2013 (aOR=1.40, 95% CI 1.33 to 1.47), vasopressor use decreased from 71.6% in 1999 to 35.5% in 2001 (aOR=0.90, 95% CI 0.82 to 0.98). Among PDD, PC use increased from 0.2% in 2009 to 4.9% in 2013 (aOR=2.05, 95% CI 1.60 to 2.63) and cardiopulmonary resuscitation use decreased from 17.6% in 2009 to 10.0% in 2013 (aOR=0.83, 95% CI 0.76 to 0.90) |
| Kurozumi et al (2019)  Unclear method  Observational | The purpose of the present study was to investigate the relationships between palliative care conferences and positive outcomes of palliative care for patients with end stage heart failure using the survey data. | Japan | Other healthcare professionals. 544 participants. Surveys | Conferences, courses, workshops | The institutions that had held such conferences (n = 223) had a larger number of hospital beds, beds in the cardiovascular department, and patients admitted to the cardiovascular department, compared with institutions that had not held these conferences (n = 321). The usage rates of opioids, non-steroidal anti-inﬂammatory drugs, and sedatives were signiﬁcantly higher in institutions that held these conferences. Multivariate analysis revealed that nutritionists and medical social workers had greater involvement in the improvement of mental symptoms and ensuring that patients could stay where they wished, respectively. The presence of palliative care physicians, physical therapists, or pharmacists was associated with multiple positive outcomes. |
| Lau et al (2018)  Quantitative  Unspecified design | Objective: We assessed whether there were comparable differences in the care of a dying patient when the CMOS was utilized and when it was not. | Canada | Patients. 83 participants. Existing register data | Multi-professional group meetings; Reminders or triggers, check-lists, templates; Other organizational strategies | Of 83 patients, 56 (67%) received initiation of the CMOS and 27 (33%) did not for EOL care. There was significant involvement of spiritual care with the CMOS (66%), as compared to the group without CMOS (19%), P < .05. The use of CMOS resulted in 1.7 adjustments to symptom management per patient by palliative care, which was significantly less than the number of symptom management adjustments per patient when CMOS was not used (3.3), P < .05. However, initiating CMOS did not result in a significant difference in patient distress around the time of death |
| LeBaron et al (2011)  Mixed methods  Descriptive/Explorative | The primary purpose of this descriptive study was to design, implement, and evaluate a program of advanced training of a palliative care resource nurse (PCRN) team and to explore the benefits of the program from the perspectives of the PCRN participants. Secondary aims included (1) empower PCRNs to advocate for optimal patient symptom management, serve as mentors and knowledge disseminators, and identify appropriate referrals for formal palliative care consultation; (2) foster community partnerships in building a university medical center’s (UMC’s) capacity to deliver palliative care services; and (3) provide a forum for professional collaboration among community stakeholders.21 Another key goal of this study was to propose a strategy for training acute-care nurses in palliative care that may be useful to similar institutions and to relate ‘‘lessons learned’’ that may assist others interested in palliative care nursing education. | USA | Nurses. 15 participants. Surveys; Group interviews | Conferences, courses, workshops; Other social interaction strategies | Quantitative and qualitative data collected suggest that palliative care resource nurse teams can help improve the frontline delivery of palliative care in the acute care setting and that hospital-based nurses are eager for this information and recognize its relevance to their clinical practice. |
| Lee et al (2013)   Mixed methods  Unspecified design | A knowledge transfer program was carried out to improve knowledge in end-of-life care staff at all levels in residential care homes for the elderly, using a model similar to that developed for a non-acute care hospital setting. | Hong Kong | Nurses, Other healthcare professionals, Patients, Families. Unclear number. Surveys; Individual interviews | Other social interaction strategies | Knowledge gaps among RCHE staff existed in the areas of mortality relating to chronic diseases, pain and use of analgesics, feeding tubes, dysphagia, sputum management, and attitudes towards end-of-life care issues, which improved after the program. From the qualitative study, RCHE staff highlighted knowledge and service gaps, issues relating to use of feeding tubes and refusal to eat, lack of conﬁdence in managing the dying process, application of Advance Care Plan (ACP) in the RCHE setting, and the need for training in these areas. Residents and family members highlighted the preference for death over suffering, planning for death, misconceptions about life-sustaining |
| Lesperance et al (2014)  Qualitative  Unspecified design | The main goal of this project was to educate and train mid-level providers (ARNPs, PAs, and LCSWs) from 3 to 4 community-based medical oncology practices and from 2 to 3 academic medical centres on the principles of palliative care for patients with metastatic cancer and on the presentation of the Five Wishes document7 to these patients in the outpatient setting, with the ultimate goal of providing these palliative care interventions within their respective oncology practices. | USA | Unspecified healthcare professionals, Patients. 11 participants. Other | Conferences, courses, workshops; Other | Of the 11 mid-level providers, 8 participated in the follow-up session, and 9 of the 11 providers implemented advanced directive’s discussions and symptom assessment and management for patients with metastatic cancer. Main barriers included uncertainties about reimbursement, patients’ lack of knowledge about palliative care, and lack of access to supportive services. This program successfully promoted advanced directive discussions and supportive/palliative care symptom assessment and management to community oncology practices, which will hopefully translate into improved quality of life for patients with metastatic cancer. |
| Liang et al (2016)  Mixed methods  Unspecified design | The overarching aim of this project was to develop decentralized specially trained staff nurses as PNCs, 2 PNCs for each of the 44 inpatient nursing units to enhance NNH knowledge and comfort in the delivery of EOL care. | USA | Nurses. Unclear number. Surveys | Other dissemination or educational strategies | The difference in total scores was not significant, although there were significant positive improvements in individual questionnaire items. Overall, the peer nurse coach model holds promise as a mechanism for engaging new nurse hires and for improving end-of-life care |
| Lin et al (2010)   Mixed methods  Observational | To explore the prevalence and reasons for implementation of hospital-wide and ICU practices relevant to quality care in key end-of-life care domains, and to discern major structural determinants of practice implementation. | USA | Other. 129 participants. Surveys; Individual interviews | Other | The response rate was 74% (129 of 174). The prevalence of hospital and ICU practices ranged from 95% for a hospital-wide formal code policy to 6% for regularly scheduled family meetings with an attending physician in the ICU. Most practices had less than 50% implementation; most were implemented primarily for quality improvement or to keep up with the standard of care. In a multivariable model including hospital structural characteristics, only hospital size independently predicted the presence of one or more hospital initiatives (ethics consult service, OR 6.13, adjusted p=0.02; private conference room in the ICU for family meetings, OR 4.54, adjusted p<0.001). |
| Lind et al (2017)   Qualitative  Explorative/Descriptive | the aim of this study was two-fold; to investigate the perceptions of local politicians, chief medical ofﬁcers and health professionals regarding national palliative care guidelines, and to identify obstacles to and opportunities for implementing these guidelines in acute care hospitals. | Sweden | Physicians, Nurses, Other. 46 participants. Individual interviews; Group interviews | Guidelines, toolkits, policies, tech tools | The results showed little knowledge of the two documents at all levels of the health care organisation. Palliative care was primarily described as end-of-life care and only few of the participants talked about the opportunity to integrate palliative care early in a disease trajectory. The environment and culture at hospitals, characterised by quick decisions and actions, were perceived as obstacles to implementation. Health professionals expressed need for palliative care training is an opportunity for implementation of clinical guidelines. There is a need for further implementation of palliative care in hospitals. One option for further research is to evaluate implementation strategies tailored to acute care. |
| Lind et al (2018)   Mixed methods  Explorative/Descriptive | Although hospitals have been described as inadequate place for end-of-life care, many deaths still occur in hospital settings. Although patient-reported outcome measures have shown positive effects for patients in need of palliative care, little is known about how to implement them. We aimed to explore the feasibility of a pilot version of an implementation strategy for the Integrated Palliative care Outcome Scale (IPOS) in acute care settings. | Sweden | Physicians, Nurses, Other healthcare professionals. Unclear number. Surveys; Individual interviews; Group interviews; Patient journal or record data | Conferences, courses, workshops; Consultations, facilitators, support services or helplines; Guidelines, toolkits, policies, tech tools | Factors related to the design and performance of the strategy and the context contributed to the results. The prevalence of completed IPOS in the patient’s records varied from 6% to 44% in the acute care settings. At the palliative care unit, the prevalence in the inpatient unit was 53% and the specialized home care team 35%. The qualitative results showed opposing perspectives concerning the training provided: Related to everyday work at the acute care units and Nothing in it for us at the palliative care unit. In the acute care settings, A need for an improved culture regarding palliative care was identified. A context characterized by A constantly increasing workload, a feeling of Constantly on-going changes, and a feeling of Change fatigue were found at all units. Furthermore, the internal facilitators and the nurse managers’ involvement in the implementation differed between the units. |
| Livingston et al (2013)  Mixed methods  Unspecified design | This study aimed to improve end-of-life care for people with dementia in a care home by increasing the number and implementation of advanced care wishes. | UK (England) | Unspecified healthcare professionals, Patients, Families. 164 participants. Surveys; Individual interviews; Patient journal or record data | Conferences, courses, workshops | Post-intervention there were signiﬁcant increases in documented advance care wishes arising from residents’ and relatives’ discussions with staff about end-of-life. These included do not resuscitate orders (16/22, 73% vs. 4/28, 14%; p < 0.001); and dying in the care homes as opposed to hospital (22/29, 76% vs. 14/30, 47%; p< 0.02). Bereaved relatives’ overall satisfaction increased from 7.5 (SD= 1.3) pre-intervention to 9.1 (SD= 2.4) post-intervention; t = 17.6, p = 0.06. Relatives reported increased consultation and satisfaction about decisions. Staff members were more conﬁdent about end-of-life planning and implementing advanced wishes. |
| Luckett et al (2017)  Qualitative  Other design | This qualitative sub-study aimed to explore PCPC and health professional perceptions of the beneﬁts of facilitated case conferencing and identify factors inﬂuencing implementation. | Australia | Physicians, Nurses, Other healthcare professionals. 37 participants. Individual interviews | Conferences, courses, workshops; | Perceived beneﬁts of facilitated case conferencing included better communication between staff and families, greater multi-disciplinary involvement in case conferences and care planning, and improved staff attitudes and capabilities for dementia palliative care. Key factors inﬂuencing implementation included: stafﬁng levels and time; support from management, staff and physicians; and positive family feedback. ... The facilitated approach, led by PCPCs, seems to have been successful in overcoming many of the barriers found to impede less structured case conferencing, described in detail as follows. |
| Mackenzie et al (2011)  Mixed methods  Other design | To evaluate the role and value of a New Zealand National LCP Ofﬁce (NZ LCP Ofﬁce) from the perspective of key stakeholders. | New Zealand | Other. 64 participants. Surveys; Individual interviews | Consultations, facilitators, support services or helplines; Guidelines, toolkits, policies, tech tools; Financial interventions; Other | When considering the role of the NZ LCP Ofﬁce, key stakeholders identiﬁed two core services as highly important, namely the promotion of the sustainable implementation of LCP and the provision of a national LCP information network. Other key initiatives identiﬁed by key stakeholders as important included the NZ LCP Ofﬁce continuing to work in consultation and collaboration with the LCP Central Team and to be a voice for end-of-life care issues in New Zealand. The value or beneﬁt of the NZ LCP Ofﬁce was endorsed, in that service performance was rated as good or very good by at least 90% of the respondents, plus 40% of participants perceived the NZ LCP Ofﬁce had contributed to positive changes in LCP document compliance, program integrity and improvement in care of the dying to a moderate extent. |
| Maeda et al (2014  Quantitative  Unspecified design | To evaluate changes in the structure and processes of palliative care  services after implementation of the Cancer Control Act. | Japan | Other. Unclear number. Surveys | Other | The response rates were $99%. All domains showed an increasing  trend (P < 0.001). There were signiﬁcant increases in full-time PCT physicians (27.4%e45.7%, Ptrend < 0.001), full-time PCT nurses (38.9%e88.0%, Ptrend < 0.001), and the median number of annual referrals to PCTs (60e80 patients, P< 0.001). Essential drugs were available in most DCCHs from baseline. Although outpatient clinics increased signiﬁcantly (27.0%e58.9%, Ptrend < 0.001), community outreach programs did not (9.0%e12.6%, P¼ 0.05). Basic education was actively introduced for in-hospital physicians and nurses (78.2% and 91.4% in 2010), but often unavailable for regional health care providers (basic education for regional physicians and nurses: 63.9% and 71.1% in 2010). |
| Mason et al (2015)  Mixed methods  Unspecified design | This paper reports an evaluation of the first two years  of the DES. It provides an insight into the impact of the DES and the practical issues faced by GPs in attempting to deliver high-quality palliative care in the community. |  | Physicians, Other healthcare professionals. Unclear number. Individual interviews; Patient journal or record data; Other | Other | In 2012-13, 72 % of patients who died of cancer were listed on the palliative care register (PCR) before death while 27 % of patients who died as a result of non-malignant conditions were listed on the PCR. In 2013-14, cancer identification remained the same but identification of people dying with other long-term conditions had improved to 32.5 %. We identified several key issues needed to improve palliative care in the community. The need for training to identify patients with palliative care needs (particularly non-cancer); communication skills training; improvements in sharing information across the NHS; under-resource of and lack of coordination with district nurses; improvements in information technology; and tools for working with enlarged palliative care registers. |
| McConnell et al (2015)  Qualitative  Case study | The main objective of this research was to identify the influences that facilitated or hindered successful LCP implementation. | Northern Ireland | Physicians, Nurses, Unspecified healthcare professionals, Other. 22 participants. Individual interviews | Other social interaction strategies; Guidelines, toolkits, policies, tech tools | Key resource inputs included facilitation with a view to maintaining LCP ‘visibility’, reducing anxiety among nurses and increasing their confidence regarding the delivery of end-of life care; and nurse and medical education designed to increase professional self-efficacy, and reduce misuse and misunderstanding of the LCP. Key enabling contexts were consistent senior management support; ongoing education and training tailored to the needs of each professional group; and an organisational cultural change in the hospital setting that encompassed end-of-life care |
| McGrath et al (2013)  Quantitative  Unspecified design | The authors sought to assess  the cost effectiveness of palliative care in a community hospital setting by comparing cost per day of patients receiving palliative care to cost per day for patients not receiving palliative care. Three research questions guided the study: (a) What are the costs per day for patients receiving palliative care? (b) Can the costs of palliative care services be covered by cost savings in aggressive treatment? (c) Is there a statistically significant difference in costs of care between patients who receive palliative care and those who do not? The analyses were planned as daily, weekly, and end-of-hospitalization cost comparisons. | USA | Patients. 201 participants. Existing register data | Conferences, courses, workshops; Mass media campaigns or other public campaigns; Multi-professional group meetings; Other social interaction strategies | Key success factors for implementing the palliative care program included assessing the facility's desire to implement palliative care and a readiness for major change in medical and nursing practice. |
| Moore et al (2017)  Mixed methods  Other design | To (1) understand how the Intervention operated in nursing homes in different health economies; (2) collect preliminary outcome data and costs of an interdisciplinary care leader (ICL) to facilitate the Intervention; (3) check the Intervention caused no harm. | UK | Nurses, Unspecified healthcare professionals, Patients, Families. 41 participants. Individual interviews; Observations; Existing register data; Other | Conferences, courses, workshops; Multi-professional group meetings; Other social interaction strategies; Other organizational strategies; Other | Contextual differences were identified between sites: nursing home 2 had lower involvement with external healthcare services. Core components were implemented at both sites but multidisciplinary meetings were only established in nursing home 1. The Intervention prompted improvements in advance care planning, pain management and person-centred care; we observed no harm. Six-month ICL costs were £18 255. |
| Mun et al (2016)  Quantitative  Quality improvement | Incorporate palliative care into the routine ICU workflow to increase the numbers of palliative care consultations, improve end-of-life care in the ICU, and demonstrate an impact on ICU and/or hospital LOS. | USA | Patients. 198 participants. Patient journal or record data | Reminders or trigger, check-lists, templates; Guidelines, toolkits, policies, tech tools | A comparison between pre- and postintervention data showed positive trends  in measured outcomes, including increased early identification of advance directives, code status, and goals of care along with a decrease in ICU LOS and hospital LOS. In addition, the number of ICU family meetings and palliative care consultations increased. |
| Mun et al (2018)   Quantitative  Unspecified design | Improve end-of-life care in the ICU by incorporating basic palliative care processes into the daily routine ICU workflow, thereby reserving the palliative care team for refractory situations. | USA | Patients. 198 participants. Patient journal or record data | Reminders or trigger, check-lists, templates; Guidelines, toolkits, policies, tech tools | Early identification of Goals-of-Care, advance directives, and code status by the ICU staff led to a proactive ICU family meeting with resultant increases in changes in code status and treatment. The numbers of palliative care consultations also rose, but not significantly. |
| Namisango et al (2016)  Quantitative  Unspecified design | A mHealth approach was adopted to explore ways of strengthening the supply chain and service delivery components of palliative care pharmaceutical systems. This study reports on the piloting of an electronic pharmaceutical management application, accessed via tablet computers, to capture key patient and medicines data as part of opioids management in palliative care services. | Uganda | Patients. 455 participants. Observations; Other | Conferences, courses, workshops; Consultations, facilitators, support services or helplines; Feedback to stakeholders; Other social interaction strategies; Other | Improvements in all measures were identified at both sites. The application supported the registration and management of 455 patients and a total of 565 consultations. Improvements in both time efficiency and medicines management were noted. Time taken to collect and report pharmaceuticals data was reduced from 7 days to 30 min and10daysto1hatthe urban hospice and rural hospital respectively. Stock expiration reduced from 3 to 0.5 % at the urban hospice and from 58 to 0 % at the rural hospital. Additional observations relating to the use of the application across the two sites are reported. |
| Neo et al (2012)  Quantitative  Unspecified design | In this study, we investigate whether adoption of a modiﬁed LCP in a Singapore hospital translated to better end-of-life care for cancer patients. | Singapore | Patients. 60 participants. Other | Conferences, courses, workshops; Consultations, facilitators, support services or helplines; Guidelines, toolkits, policies, tech tools; Other | Five types of end-of-life symptoms were analysed. There was only 1 uncontrolled symptom at death in the post-implementation group compared to 24 uncontrolled symptoms in the retrospective audit group. The prescription of breakthrough medications for symptom control increased from 21% in the retrospective audit group to 79% in the post-implementation group. Inappropriate monitoring was discontinued in 25 patients in the post-implementation group compared to none in the retrospective audit group. The documentation of resuscitation status and religion of the patient was improved, achieving full documentation in the post-implementation group. |
| Nikbakht-Van de Sande et al (2014)   Qualitative  Participatory/action research | Here we report on the design and evaluation of the CPPR. The leading research questions are as follows. (1) What were the reasons for developing a new care programme for patients receiving palliative radiotherapy? (2) How was it designed and implemented? (3) How did patients and healthcare professionals perceive the outcomes in terms of satisfaction, attention to patients’ needs, and sustainability of the programme? | The Netherlands | Physicians, Nurses, Other healthcare professionals, Patients. 29 participants. Individual interviews; Group interviews; Observations | Other dissemination or educational strategies; Consultations, facilitators, support services or helplines | After a shift from inpatient to outpatient radiotherapy treatment, patients and healthcare professionals perceived shortcomings in the oncological chain care. The CPPR was developed in a participative way giving a key role to the NP. Evaluation after implementation of the programme showed that patients and professionals were predominantly positive about its effects. However, implementation was not sustained due to lack of institutional and managerial support. |
| Noble et al (2018)   Qualitative  Other design | This study aimed to investigate if and how EOL care excellence can be embedded or normalised in acute healthcare settings. Our objectives were to: 1) generate a rich description of individual and contextual barriers and enablers surrounding implementation of the Clinical Guidelines for Dying Patients (CgDp), in an acute setting; 2) identify learning strategies to mitigate barriers and strengthen enablers; 3) integrate our data analysis, using normalization process theory (NPT) (see below), to generate a conceptual model to inform further implementation research. | Australia | Physicians, Nurses, Other healthcare professionals, Unspecified healthcare professionals. 28 participants. Individual interviews; Group interviews | Pathways or programmes | The CgDp afforded staff support, but the reality of the clinical process was invariably perceived as more complex than the guidelines suggested. The CgDp ‘made sense’ to nursing and medical staff, but, because allied health staff were not ward-based, they were not as engaged (coherence). Implementation was challenged by competing concerns in the acute setting where most patients required a different care approach (cognitive participation). The CgDp is designed to start when a patient is dying, yet staff found it difficult to diagnose dying. Staff were concerned that they lacked ready access to experts (collective action) to support this. Participants believed using CgDp improved patient care, but there was an absence of participation in real time monitoring or quality improvement activity. |
| Noome et al (2017)  Mixed methods  Experimental | The aim of this study was to examine the effectiveness of supporting intensive care units on implementing the guidelines. | The Netherlands | Nurses, Families. Unclear number. Surveys; Individual interviews | Conferences, courses, workshops; Educational outreach visits; Other dissemination or educational strategies; Other social interaction strategies; Pathways or programmes | Overall, an increase in adherence to the guidelines was found in both groups. Overall, use of the guidelines in the intervention group was higher, but on some aspects the control group showed a higher score. Care for the patient and the overall nursing care scored signiﬁcantly higher according to family in the intervention group. |
| Norton et al (2011)  Mixed methods  Other design | To describe the institutionalization of a new PCCS in a quaternary  care academic medical centre (AMC) and highlight two themes, cost and quality, that pervaded the dynamics involved from the inception to the successful integration of the service. | USA | Physicians, Other healthcare professionals, Other. 79 participants. Individual interviews; Observations; Other | Multi-professional group meetings; Financial interventions | Key infrastructure components that contributed to the successful  integration of the PCCS included top level interprofessional administrative buy-in to the quality and cost arguments for PCCS, PCCS leadership selection, robust data collection strategies emphasizing quality outcome data, the adoption of the  ‘‘physician referral only’’ rule, and incremental and sustainable growth. The PCCS service grew an average of 23% per year from 2003 to 2009. |
| O'Brien et al (2016)  Qualitative  Unspecified design | To explore the experiences of the facilitators of the programme, specifically with regard to the implementation approach they had adopted. To obtain a detailed account of the impact of Six Steps  on individual care homes. | UK (England) | Unspecified healthcare professionals. 16 participants. Surveys; Individual interviews; Other | Conferences, courses, workshops; Educational outreach visits; Consultations, facilitators, support services or helplines; Other social interaction strategies; Guidelines, toolkits, policies, tech tools; Other organizational strategies; | The programme was flexibly designed so that it could be individually tailored to the geographical location and the individual cohort requirements. Facilitators provided comprehensive and flexible support to care homes. Challenges to programme success were noted as; lack of time allocated to champions to devote to additional programme work, inappropriate staff selected as ‘Champions’ and staff sickness/high staff turnover presented challenges to embedding programme values. Benefits to completing the programme were noted as; improvement in Advance Care Planning, improved staff communication/confidence when dealing with multi-disciplinary teams, improved end-of-life processes/documentation and increased staff confidence through acquisition of new knowledge and new processes. |
| Oosterveld-Vlug et al (2019)  Mixed methods  Unspecified design | the aim of this process evaluation study was to provide a more detailed understanding of the implementation of the PACE Steps to Success Programme, across countries and within specific countries. Specifically, we sought to assess the programme’s reach, adoption, implementation and the intention of staff to Maintain the PACE Steps to Success Programme in future practice, and the factors affecting these. | Europé | Other health care professionals, Other 269 participants Surveys; Group interviews; Other | Pathways or programmes; Other dissemination or educational strategies | The performance of the PACE Programme on the RE-AIM components was highly variable within and across countries, with a high or medium score for in total 28 (out of 37) LTCFs on Reach, for 26 LTCFs on Adoption, for 35 LTCFs on Implementation and for 34 LTCFs on intention to Maintenance. The factors affecting performance on the different RE-AIM components could be classified into three major categories: (1) the PACE Programme itself and its way of delivery, (2) people working with the PACE Programme and (3) contextual factors. Several country specific challenges in implementing the PACE Programme were identified. |
| Ouchi et al (2014)  Quantitative  Quality improvement | The study’s objective was to track the rate of ED-initiated PC consultation for patients with advanced dementia (AD) after an educational intervention, and to categorize decision making for physicians who chose not to initiate consultation. | USA | Physicians, Patient. 74 participants. Surveys; Patient journal or record data | Conferences, courses, workshops; Other decision-support strategies; Guidelines, toolkits, policies, tech tools | Patients (N= 548) over 70 who visited the ED were approached and 304 completed the screening. Fifty-one of the 304 met criteria for AD. Their average age was 86; 33% were male. Eighteen of the 51 (35%) patients received a PC consultation sometime during their ED or hospital stay. Four of the 18 (22%) consultations were ED initiated. In 23 of 51 (45%) unique cases, physicians responded to the questionnaire. The majority felt that a PC consult was not appropriate for patients based on their knowledge, attitudes, or beliefs. |
| Pesut et al (2015)  Mixed methods  Unspecified design | The purpose of this community-based research project is to test the feasibility and identify potential outcomes of implementing a rural palliative supportive service (RPaSS) for older adults living with life-limiting chronic illness and their family caregiver in the community. This paper reports on the feasibility aspects of the study. | Canada | Patients, Families. 33 participants. Individual interviews; Other | Educational outreach visits; Multi-professional group meetings; Consultations, facilitators, support services or helplines; Other social interaction strategies; Other organizational strategies | A community-based advisory committee worked with the investigative team over a 1-year period to plan RPaSS, negotiating the best fit between research methods and the needs of the community. Recruitment took longer than anticipated with service capacity being reached at 8 months. Estimated service capacity of one nurse coordinator, based on bi-weekly visits, is 25 participants and their family caregivers. A total of 393 in-person visits and 53 telephone visits were conducted between January 2013 and May 2014. Scheduled in-person visit duration showed a mean of 67 minutes. During this same time period only 19 scheduled visits were declined, and there was no study attrition except through death, indicating a high degree of acceptability of the intervention. The primary needs that were addressed during these visits have been related to chronic disease management, and the attending physical symptoms were addressed through teaching and support. The use of structured quality of life and family caregiver needs assessments has been useful in facilitating communication, although some participants experienced the nature of the questions as too personal in the early stages of the relationship with the nurse coordinator. |
| Porzio et al (2013)   Quantitative  Unspecified design | To evaluate the efficacy of a home care program, closely integrated with a medical oncology department. | Italy | Patients. 461 participants. Patient journal or record data | Multi-professional group meetings; Other organizational strategies | A total of 461 patients was followed at home for a total of 10,503 home accesses (median accesses/patient, 20; range, 1-159). The median length of home care was 76 days (range, 2-643 days}. The median was 101 days for patients coming from the medical oncology department and 53 days for patients coming from other origins (P <0.0005). There were 428 emergency calls (4.1 % of all the home accesses}. Emergency calls accounted for 253 of 7,364 home accesses (3.4 %} among patients coming from the medical oncology department and for 175 of 3,139 home accesses (5.6%) among patients coming from other origins (P = 0.00005). Eighty of 461 patients (17.3%} required one in-hospital admission and 19/461 patients (4.1%) more than one. Fifty-nine of259 (17.8%) patients coming from the medical oncology department and 40 of 186 (26.9%) coming from other origins required in-hospital admissions (P = 0.04). A total of 311 patients died (163 coming from the medical oncology department and 148 from other origins). Twenty-eight of 163 (17.1%} coming from the medical oncology department and 52 of 148 (35.1 %) coming from other origins died in the hospital (P = 0.0002). |
| Pype et al (2015)   Mixed methods  Quasi-experimental | : To describe the development and evaluation of a training programme for nurses in primary care. The programme aimed to prepare palliative home care team nurses to act as facilitators for general practitioners’ workplace learning. | Belgium | Nurses. Unclear number. Individual interviews; Other | Conferences, courses, workshops; Consultations, facilitators, support services or helplines | A total of 35 nurses followed the programme. The overall satisfaction was high. Homework assignments interfered with the practice workload but showed to be fundamental in translating theory into practice. Median score on the summative assessment was 7 out of 14 with range 1–13. Interviews revealed some aspects of the training (e.g. incident analysis) to be too difficult for implementation or to be in conflict with personal preferences (focus on patient care instead of facilitating general practitioners’ learning). |
| Raijmakers et al (2014  Qualitative  Other design | This study evaluates barriers and facilitators to its implementation in the Netherlands from the perspective of key stakeholders, to inform future implementation processes. | The Netherlands | Physicians, Nurses, Other healthcare professionals, Other. 28 participants. Individual interviews; Group interviews | Conferences, courses, workshops; Other social interaction strategies; Other decision-support strategies; Pathways or programmes; Other organizational strategies; Other | According to the stakeholders, a context analysis prior to implementation was useful to find the appropriate orientation to adequately motivate healthcare professionals as well as management. The main contributing factors were the quality of the LCP (including its evidence-based character and completeness), and that it fitted the needs of healthcare professionals. During the implementation phase, a multidisciplinary project team, competent support and continuous monitoring were identified as important facilitators. Furthermore, for successful implementation, a facilitator working in liaison with others was helpful. To guarantee sustainability of the use of the LCP, it was important to disentangle tasks from the project leader and formally integrate these into the quality systems of the organisation. |
| Rauenzahn et al (2017)   Quantitative  Quality improvement | Our aims were to quantitatively describe the palliative referral rates and symptom burden in a South Texas cancer centre and establish a palliative referral system by implementing the Edmonton Symptom Assessment Scale (ESAS). | USA | Patients. Unclear number. Surveys | Conferences, courses, workshops | On average, one patient per month was referred before implementation of the intervention compared with 10 patients per month after implementation across all clinics. In ﬁve sample clinics, 607 patients completed the initial assessment, and 430 follow-up forms were collected over 5 months, resulting in a total of 1,037 scores collected in REDCap. The mean ESAS score for initial patient visits was 20.0 (standard deviation, 18.1), and referred patients had an initial mean score of 39.0 (standard deviation, 19.0). |
| Raunkiaer & Timm (2010)  Qualitative  Unspecified design | The purpose of this study was to evaluate an attempt to develop—through three pedagogical methods—the palliative care competencies of the personnel and make organizational improvements at three Danish nursing homes. | Denmark | Nurses, Unspecified healthcare professionals, Patients, Families, Other. 97 participants. Group interviews | Conferences, courses, workshops; Other social interaction strategies; Other organizational strategies; Other | Results: Both the nursing home employees and the teachers felt that the project as a whole, and the three methods used, contributed to an improvement in the staff ’s palliative care competencies and the organization. |
| Reymond et al (2011)  Quantitative  Unspecified design | The objective of this study was to develop, implement and evaluate an end-of-life (tenninal) care pathway and associated infrastructure suitable for Australian residential aged care facilities that improves resident and health system outcomes | Australia | Nurses, Patients, Families. 510 participants. Surveys; Others | Conferences, courses, workshops; Consultations, facilitators, support services or helplines; Other social interaction strategies; Reminders or triggers, check-lists, templates; Pathways or programmes; Other organizational strategies | Results indicated that the pathway, delivered within a care framework that guides provision of palliative care, resulted in improved resident outcomes and decreased inappropriate transfers to acute care settings. |
| Rocque et al (2015)  Quantitative  Quasi-experimental | To identify the impact of implementing triggered palliative care consultation (TPCC) as part of standard care  for patients admitted to the solid-tumor oncology service with advanced cancer. |  | Patients. 203 participants. Surveys; Patient journal or record data | Consultations, facilitators, support services or helplines; Reminders or triggers, check-lists, templates; Other organizational strategies | Sixty-ﬁve patients were evaluated before TPCC implementation (Cohort 1). Seventy patients (Cohort 2) were  evaluated after initiation of TPCC, and 68 patients (Cohort 3) were evaluated after modiﬁcations based on implementation barriers identiﬁed in Cohort 2. The percentage of patients correctly identifying their cancer as incurable increased from 65% in Cohort 1 to 94% in Cohorts 2 and 3. TPCC had minimal impact on hospice utilization, cost of care, survival, patient reported symptoms, and patient satisfaction, likely because of the limited nature of the intervention. Implementation was challenging, with only 60% of patients in Cohort 2 and 62% in Cohort 3 receiving TPCC. Overall, the intervention was viewed favourably by 74% of oncologists. |
| Sánchez et al (2014)   Quantitative  Observational | The aim of this study was to determine whether health professionals have incorporated the requirements of this legislation into their clinical practice and whether there have been improvements in decision-making procedures affecting the quality of dying in hospitals. | Spain | Patients. 398 participants. Patient journal or record data | Other | Provision of information on measures to facilitate comfort and the relief of physical suffering increased from 15.7% to 22.0%, although this was not significant. There was a significant increase in the number of patients who received joint counselling in this regard from physicians and nurses, from 0% in 2009 to 7.1% in 2011. |
| Schweitzer et al (2016)  Quantitative  Experimental design | The aim was to evaluate the effect of the implementation of an information handover form regarding patients receiving palliative care. Outcome was the information available for the out-of-hours GP co-operative. | The Netherlands | Physicians. 426 participants. Existing register data; Patient journal or record data | Conferences, courses, workshops; Other decision-support strategies; Other organizational strategies | Overall information was transferred by the GPs in 179 of the 772 first palliative contacts (23.2%). The number of contacts in the experimental group in which information was available increased significantly after intervention from 21% to 30%, compared to a decrease from 23% to 19% in the control group. The training had no additional effect. The content of the transferred information was adequate in 61.5%. There was no significant difference in the quality of the content between the groups. |
| Selman et al (2016)  Mixed methods  Quasi-experimental design | To evaluate course participants’ self-rated confidence, competence and knowledge of EoLC topics. | UK | Physicians, Nurses, Other healthcare professionals, Other. 236 participants. Surveys | Conferences, courses, workshops | All 14 self-assessment topics improved significantly (p<0.001); most improved was ‘understanding and implementing Fast Track discharge’. Qualitative data showed increased knowledge and confidence in EoLC, particularly in communication, commitment to team work and holistic care. Overall, 217 (92%) participants would recommend the course and 215 (98%) indicated it would influence their practice. |
| Selvaggi et al (2014)  Quantitative  Unspecified design | In this paper, we describe a quality improvement project that sought to close this gap at a tertiary care hospital in Pittsburgh, Pennsylvania, from August 2006 to May 2010. |  | Physicians, Patients. 256 participants. Surveys | Conferences, courses, workshops; Multi-professional group meetings; Consultations, facilitators, support services or helplines; Other | During the program, 392 PC consultations were provided to 256 unique patients. Of these 256 patients, the PC clinicians documented the first goals of care conversations in 67% of patients (n = 172). Of the 278 consults referred for pain, 70% (n = 194) involved reports of unacceptable or very unacceptable pain at baseline. Sixty-six percent (n = 129) of these 194 consults involved reports of pain that was acceptable or very acceptable within 48 hours of consultation. In addition, the hospice referral rate grew from a pre-implementation rate of 5% to 41% (n = 67) of 165 patients who died during the period of program implementation. Lastly, haematological oncologists reported high levels of satisfaction with the program. |
| Seow et al (2010)   Quantitative  Unspecified design | The objective of this study was to evaluate whether Ontario's End-of-Life Care Strategy, which aimed to shift care from acute settings to the home, improved performance on quality indicators for end-of-life home care patients in use of more home care services and fewer acute care services. | Canada | Patients. 9 368 participants. Existing register data or journal data | Other | Among home care patients (n = 9,368), per-patient use  of home care and acute care did not change significantly over the time periods. The pattern of average nursing and PSW hours per week used in the last 12 weeks of life did not change a year after the strategy implementation, averaging 3.8 nursing hours per week and 3.8 PSW hours per week over the study period. The proportion of in-hospital deaths remained stable at approximately 38% over the study period. Approximately 16% of patients had an ED visit and 32% had a hospitalization in the last two weeks of life across time periods. Similarly, the proportion of patients who had zero, one or greater than one ED visit or hospitalization remained constant across time periods. |
| Shah et al (2013)   Quantitative  Unspecified design | The development of healthcare information systems in the developing world has been driven primarily by the need to report aggregate statistics to the government or funders [17]. Toward this end, this study describes the development and evaluation of DataPall, a new EMR catered to palliative care providers in low-resource settings. | Malawi | Unspecified healthcare professionals. 17 participants. Observations; Other | Guidelines, toolkits, policies, tech tools | In a study of health professionals in a Malawian hospital, DataPall enabled palliative care providers to find patients’ appointments, on average, in less than half the time required to locate the same record in current paper records. Moreover, participants generated customizable reports documenting patient records and comprehensive reports on providers’ activities with little training necessary. Participants affirmed this ease of use on the system usability scale. |
| Singh et al (2018)   Quantitative  Quality improvement | We introduced the surprise question (SQ), “Would I be surprised if this patient died in the next 1 year, 6 months, and 1 month?” at multidisciplinary rounds to increase palliative care referrals through the introduction of this prognostic prompt. | USA | Physicians, Nurses, Other healthcare professionals, Patients. 205 participants. Existing register data | Other decision-support strategies | Regular discussion of prognosis of patients with cancer in an inpatient medical setting did not increase referrals to inpatient or outpatient palliative care or hospice. Increased clinical experience impacted hospital medicine providers and bedside nurses’ estimation of prognosis differently than oncology providers. Medical oncologists were significantly more optimistic than hospital medicine providers. |
| Slort et al (2013)  Quantitative  Experimental design | In this paper we report on a controlled clinical trial which evaluated the effectiveness of this ACA training programme on GP-patient communication in palliative care | The Netherlands | Physicians. 126 participants. Observations; Other | Educational materials; Conferences, courses, workshops | Sixty-two GPs were assigned to the intervention and 64 to the control group. We found no effect of the ACA training programme on how the GPs communicated with the patient or on the number of issues discussed by GPs with the patient. The total number of issues discussed by the GPs was eight out of 13 before and after the training in both groups. |
| Slort et al (2014)  Quantitative  Experimental design | To evaluate the effectiveness of the Availability, Current issues and Anticipation training programme for general practitioners on patient-reported outcomes. | The Netherlands | Physicians, Patients. 326 participants. Surveys | Educational materials; Conferences, courses, workshops | Questionnaire data were available for 145 patients (89 in intervention and 56 in control group). We found no significant differences over time between the intervention and control groups in any of the five outcome measures. Ceiling effects were observed for the Rest & Peace Scale, Patient Satisfaction Questionnaire–III and Availability, Current issues and Anticipation Scale. |
| Smith et al (2019)   Quantitative  Quality improvement | The purpose of this quality improvement (QI) project was 2-fold: (1) improve PC service measures (PC referral rate and time to PC referral) and (2) improve patient outcomes (LOS, direct cost, transition of care, 30-day mortality) for hospitalized adult patients following implementation of IDRs on a hospitalist service in a non-ICU setting. |  | Patients. 800 participants. Existing register data; Other | Multi-professional group meetings; Other organizational strategies | Results support IDRs as a mechanism to improve time to PC referral, decrease LOS, direct cost, and 30-day mortality among hospitalized adults. A more objective method of identifying patients with unmet PC needs may be warranted. |
| Tan et al (2014)   Quantitative  Unspecified design | A physician order form to withhold inappropriate life-sustaining interventions was initiated in 2009. The use of the form was facilitated by staff educational sessions and a palliative care consult service. This study aims to evaluate the impact of these interventions in 2010. | Singapore | Patients. Unclear number. Patient journal or record data | Conferences, courses, workshops; Consultations, facilitators, support services or helplines; Other decision-support strategies; Other organizational strategies | There was an increase in orders to withhold life-sustaining therapies, such as cardiopulmonary resuscitation (66.2%–80.0%). There was a decrease in burdensome interventions such as antibiotics (44.9%–24.9%) and a small increase in palliative treatments such as analgesia (29.1%–36.7%). There were more discussions on the role of cardiopulmonary resuscitation with conversant patients (4.6%–10.2%) and families (56.5%–79.8%) (p-value all < 0.05). On multivariate analysis, the physician order form independently predicted orders to withhold cardiopulmonary resuscitation. |
| Temkin-Greener et al (2018)  Mixed methods  Experimental design | Test the impact of PC teams on end-of-life outcomes. | USA | Nurses, Unspecified healthcare professionals, Patients, Other. Unclear number. Surveys; Individual interviews; Existing register data; Patient journal or record data | Conferences, courses, workshops; Other organizational strategies | Overall, we found no statistically signiﬁcant effect of the intervention. However, independent analysis of the interview data found that only 6 of the 14 treatment facilities had continuously working PC teams throughout the study period. Decedents in homes with working teams had signiﬁcant reductions in the odds of in-hospital death compared to the other treatment [odds ratio (OR), 0.400; P<0.001), control (OR, 0.482; P<0.05), and nonrandomized control NHs (0.581; P<0.01). Decedents in these NHs had reduced rates of depressive symptoms (OR, 0.191; P≤0.01), but not pain or hospitalizations. |
| Thurston & Waterworth (2012)   Qualitative  Unspecified design | To explore nurses’ experiences of a top-down change that involved the introduction of the Liverpool Care Pathway for the Dying Patient (LCP). | New Zealand | Nurses. Unclear number. Individual interviews; Other | Conferences, courses, workshops; Pathways or programmes | The nurses viewed their experience with the LCP positively, highlighting an improvement in their knowledge base regarding care of the dying. ‘Making sense’ was a key theme that reflected how the nurses attempted to manage the impact of the change on their own practice and on the nursing team. |
| Usher et al (2015)  Mixed methods  Quality improvement | New nurse hires lacked end-of-life nursing experience in the hospital, and mechanisms were unavailable to guide them. A quality improvement project was developed to address this. | USA | Nurses. 73 participants. Surveys; Observations; Other | Educational materials; Conferences, courses, workshops; Consultations, facilitators, support services or helplines | Peer nurse coach self-perceived competencies in end-of-life care delivery improved after training, at 6 months (P < .01), and at 1 year (P < .05). Qualitative findings highlighted various ways peer nurse coaches manifest these new roles. The plan-do-study-act method, the peer nurse coach approach, availability of unit-based end-of-life resources, and peer nurse coach mentoring had positive effects on peer nurse coach self-perceived end-of-life competence and their abilities to coach new nurse hires. |
| Walczak et al (2017)  Quantitative  Experimental design | To evaluate the efﬁcacy of a nurse-facilitated communication support program for patients with advanced, incurable cancer to assist them in discussing prognosis and end-of-life care. | Australia | Patients. 110 participants. Surveys | Educational materials; Conferences, courses, workshops; Other organizational strategies | Communication support program recipients gave signiﬁcantly more cues for discussion of prognosis, end-of-life care, future care options and general issues not targeted by the intervention during recorded consultations, but did not ask more questions about these issues or overall. Oncologists’ question prompt list and question asking endorsement was inconsistent. Communication support program recipients’ self-efﬁcacy in knowing what questions to ask their physician signiﬁcantly improved at follow-up while control arm patients’ self-efﬁcacy declined. The communication support program did not impact patients’ health-related quality-of-life or the likelihood that their health information or shared decision-making preferences would be met. Satisfaction with the communication support program was high. |
| Walker & Read (2010)  Qualitative  Unspecified design | The palliative care team at our inner-city community teaching hospital developed and implemented a palliative care order set (PCOS). We assessed frequency of use and effectiveness of this order set on medication availability after implementation. | UK | Physicians, Nurses. 6 participants. Other | Educational outreach visits; Guidelines, toolkits, policies, tech tools | Of 106 patients in the study group, 31 (29%) were treated using the PCOS, 6 (6%) received the CMO order, and 69 (65%) did not receive either. Patients in the PCOS group had signiﬁcantly more orders for every palliative medication ( p~0.05). Opioids and anxiolytics were made available to every patient in the PCOS group. Most PCOS patients received orders for antiemetic, antipsychotic, anti-secretion, and laxative medication during the end-of-life period. No CMO patients and few patients in the no palliative care orders group received orders for these medications. The PCOS and CMO group similarly limited nonpalliative interventions, whereas the nonpalliative group had relatively high use of these interventions until death. |
| Walker et al (2011)  Quantitative  Unspecified design | The aim of this study was to explore the doctors’ and nurses’ experiences of using the LCP in intensive care and, in particular, its potential impact on practice. | USA | Patients. 106 participants. Individual interviews | Pathways or programmes | The staff experience of the LCP was dependent on their role, with mixed reports about frequency of use and level of education received on the LCP. Education and adequate support was identified as being pivotal to the successful implementation of any type of LCP. |
| Waller et al (2012)  Mixed methods  Quasi-experimental | The aim of this study was to assess the im-  pact of the systematic and ongoing use of the Guidelines and NAT: PD-C on patient outcomes including level of need, quality of life, anxiety, and depression. It was hypothesized that using the Guidelines and NAT: PD-C would result in reductions in the unmet needs, clinical anxiety and depression of participants, and an increase in their quality of life.15 | Australia | Patients. 219 participants. Surveys; Individual interviews; | Guidelines, toolkits, policies, tech tools | Moderate to high needs across all domains were frequently seen in the  preintervention phase. The use of the NAT: PD-C was associated with a signiﬁcant reduction in health system and information and patient care and support needs. |
| Walling et al (2011)  Quantitative  Unspecified design | In order to identify quality improvement targets, we  studied a decedent sample using medical record abstraction to describe use of the protocol (including protocol adherence, timing of the protocol initiation, and dosing of opiates) and to identify patient and provider characteristics associated with potential missed opportunities for use of the ESMO protocol | USA | Patients. 496 participants. Existing register data; Patient journal or record data | Reminders or triggers, check-lists, templates | Half of patients who died received ESMO protocol care (n¼248). All had documentation of a do-not-resuscitate (DNR) order (a requirement of the protocol). An opiate drip was used for 95% of patients placed on the ESMO protocol and it was titrated up at least once for 67% of those patients. Patients had a mean of 4 opiate titrations, but for only a mean of 2.2 was a justiﬁcation documented (symptom documentation is required for each titration per the protocol). In a multivariable regression accounting for other demographic, clinical and provider variables, uninsured patients (risk ratio [RR] 0.25, 95% conﬁdence interval [CI] 0.06–0.62), patients admitted from a nursing home (RR 0.57, 95% CI 0.30–0.99), and patients considered for transplant (RR 0.60, 95% CI 0.40–0.85) were signiﬁcantly less likely to be placed on the ESMO protocol prior to death. |
| Walling et al (2015)  Quantitative  Unspecified design | Palliative care and preparation for liver transplantation are often perceived as conﬂicting for patients with end-stage liver disease (ESLD). We sought to improve both simultaneously through a case ﬁnding and care coordination quality improvement intervention. | USA | Patients. 110 participants. Existing register data; Other | Other social interaction strategies; Other organizational strategies | Results: We compared rates of consultation for 49 hospitalized veterans and compared their outcomes to 61 pre-intervention veterans. Veterans were more likely to be considered for liver transplantation (77.6% versus 31.1%, p < 0.001) and receive palliative care consultation during the intervention period, although the latter ﬁnding did not reach statistical signiﬁcance (62.5% versus 47.1%, p = 0.38). ... Conclusions: Active case ﬁnding improved consideration for liver transplantation without decreasing palliative care consultation. |
| van der Plas et al (2015)  Quantitative  Unspecified design | To compare cancer patients with and without additional support from a case manager on: 1) the patients’ general characteristics, 2) characteristics of care and support given by the GP, 3) palliative care outcomes. | The Netherlands | Physicians, Nurses, Patients. Unclear number. Surveys; Other | Consultations, facilitators, support services or helplines | The GP is more likely to know the preferred place of death (OR 7.06; CI 3.47-14.36), the place of death is more likely to be at the home (OR 2.16; CI 1.33-3.51) and less likely to be the hospital (OR 0.26; CI 0.13-0.52), and there are fewer hospitalisations in the last 30 days of life (none: OR 1.99; CI 1.12-3.56 and one: OR 0.54; CI 0.30-0.96), when cancer patients receive additional support from a case manager compared with patients receiving the standard GP care. |
| van der Plas et al (2018)   Quantitative  Quasi-experimental | To study the effects of the implementation of PaTz, and provide additional analyses on two important elements: the PaTz register and patient discussions. | The Netherlands | Physicians. Unclear number. Surveys | Multi-professional group meetings | Identification of patients with palliative care needs was done systematically for more patients after implementation of PaTz compared with before (54.3% versus 17.6%). After implementation, 64.8% of deceased patients had been included on the PaTz register. For these patients, when compared with patients not included on the PaTz register, preferred place of death was more likely to be known (88.1% of patients not on the register and 97.3% of deceased patients included on the register), GPs were more likely to have considered a possible death sooner (>1 month before death: 53.0% and 80.2%), and conversations on life expectancy, physical complaints, existential issues, and possibilities of care occurred more often (60.8% and 81.3%; 68.6% and 86.1%; 22.5% and 34.2%; 60.8% and 84.0%, respectively) |
| van der Steen et al (2012)   Quantitative  Unspecified design | To evaluate a booklet on comfort care in dementia from the perspective of family with relevant experience, and assess nursing home resident and family factors associated with evaluations. | Canada, the Netherlands, Italy | Families. 138 participants. Surveys; Individual interviews | Educational materials | Almost all families (94%) perceived the booklet as useful. Canadian and Dutch families evaluated the booklet’s contents and format favourably, whereas Italian families’ evaluations were less favourable. Almost all families endorsed roles for physicians or nurses and about half additionally accepted availability through own initiative, in print or through the Internet. Preference of timing was highly variable. |
| Weng et al (2017)  Quantitative  Unspecified design | In Taiwan, annual ED visits increased by 40%, from 4664,209 per year in 1995, the year of initiation of National Health Insurance, to 6569,247 per year in 2006, and are still increasing.[11] Overcrowding greatly impedes the implementation of hospice and palliative care in the ED, which reﬂects the fact that there was no ED in Taiwan providing hospice and palliative care before our intervention. Therefore, we implemented a novel model for Taiwan in our ED according to our internal and external resources to improve the quality of care of end-of-life ED patients and popularize this important issue. | Taiwan | Patients, Other. Unclear number. Surveys; Existing register data | Conferences, courses, workshops; Multi-professional group meetings; Consultations, facilitators, support services or helplines; Other social interaction strategies | . Compared with 4 in the preintervention period, the cases of do not resuscitate (DNR) per month increased signiﬁcantly to 30.1 in the early stage of intervention, 23.9 in late stage of intervention, and 34.6 in the postintervention period (all P<.001 compared with the preintervention period). Compared with 10.8% in the preintervention period, the ratio of DNR orders signed in the ED/total DNR orders signed in the study hospital was increased to 17.1% in early stage of intervention, 12.5% in late stage of intervention, and 22.8% in postintervention. Compared with zero in preintervention and early intervention, the cases of consultation with the hospice team increased signiﬁcantly to 19 cases per month in the late stage of intervention and postintervention. The ability of nurses in hospice and palliative care, including knowledge and the timing and method of consultation with the hospice team, was also signiﬁcantly improved. We successfully implemented a novel model of hospice and palliative care in the ED via a champion, education, and close collaboration with the hospice team, which could be an important reference for other EDs and intensive care unit in the future |
| Venkatasalu et al (2015)   Qualitative  Other design | To explore and critically examine stakeholders’ views and perceptions concerning the nurse-led Palliative Care Discharge Service in an acute hospital setting and to inform sustainability, service development and future service conﬁguration. | UK (England) | Unspecified healthcare professionals, Other. 12 participants. Individual interviews; Group interviews | Other social interaction strategies | Four key themes emerged relating to the role of the Discharge Facilitator Service: achieving preferred place of care; the Discharge Facilitator as the ‘conduit’ between hospital and community settings; delays in hospital discharge and stakeholders’ perceptions of the way forward for the service |
| Verhofstede et al (2016)  Mixed methods  Other design | The aim of this study is (1) to determine the feasibility of implementing the Care Programme for the Last Days of Life in the acute geriatric hospital setting and (2) to explore the health care professionals’ perceptions of the effects of the Care Programme on end-of-life care. | Belgium | Nurses, Physicians, Unspecified health care professionals. 18 participants. Individual interviews; Group interviews; Observations; Other | Pathways or programmes; Other organizational strategies | The process evaluation tool showed that implementing the Care Programme for the Last Days of Life in the geriatric ward was successful and thus feasible; a steering group was formed consisting of two facilitators, health care staff of the geriatric ward were trained in using the Care Guide for the Last Days of Life which was subsequently introduced onto the ward and approximately 57 % of all dying patients were cared for according to the Care Guide for the Last Days of Life. With regard to health care professionals’ perceptions, nurses and physicians experienced the Care Guide for the Last Days of Life as improving the overall documentation of care, improving communication among health care staff and between health care staff and patient/family and improving the quality of end-of-life care. Barriers to implementing the Care Programme for the Last Days of Life successfully are, among others, difficulties with the content of the documents used within the Care Programme for the Last Days of Life and the low participation rate of physicians in the training sessions and audits. |
| Verreault et al (2018)  Quantitative  Quasi-experimental | To evaluate the impact of a multidimensional intervention to improve quality of care and quality of dying in advanced dementia in long-term care facilities. | Canada | Nurses, Families. Unclear number. Surveys | Educational materials; Conferences, courses, workshops; Other dissemination or educational strategies; Consultations, facilitators, support services or helplines; Other social interaction strategies; Guidelines, toolkits, policies, tech tools; Other organizational strategies | The Family Perception of Care score was significantly higher in the intervention group than in the usual care group (157.3 vs 149.1; p = 0.04). The Comfort Assessment and Symptom Management scores were also significantly higher in the intervention group. |
| Wharton et al (2015)  Mixed methods  Unspecified design | The overall aim of the integration pilot project was to  expand patient access to PC through 3 simultaneous and related objectives (1) to enhance HBPC interdisciplinary teams’ palliative medicine expertise through education, (2) to improve the identification of veterans with PC needs by piloting the use of a validated assessment tool, and (3) to build meaningful working relationships between those team members providing primary care to veterans and those members of the PC consult team | USA | Patients, Other. 157 participants. Other. | Conferences, courses, workshops; Multi-professional group meetings; Guidelines, toolkits, policies, tech tools | Palliative Performance Scale implementation added little burden on nurses and triggered a discussion in 51 flagged patients. The tool successfully identified 75% of patients who died or were discharged. Screening was systematic and consistent and resulted in targeted discussions about PC needs without generating additional burden on our PC consult service. This model shows promise for enhancing collaborative patient care and access to PC. |
| Wickson-Griffiths et al (2015)   Qualitative  Descriptive/Explorative | Using a qualitative descriptive design described by Sandelowski (2000), the research team sought to evaluate the implementation, format, process, and outcomes of CCRs | Canada | Nurses, Other healthcare professionals. 40 participants. Individual interviews; Group interviews | Consultations, facilitators, support services or helplines; Other social interaction strategies; Other organizational strategies | Study participants identified that effective advertising, interest, and assigning staff to attend CCRs facilitated their participation. The key barriers to their attendance included  difficulty in balancing heavy workloads and scheduling logistics. Interprofessional team member representation was sought but was not consistent. Study participants recognized the benefits of attending; however, they provided feedback on how the scheduling, content, and focus could be improved. Overall, study participants found CCRs to be beneficial to their palliative and end-of-life care knowledge, practice, and confidence. However, they identified barriers and recommendations, which warrant ongoing evaluation. |
| Williams et al (2014)  Qualitative  Unspecified design | The purpose of this study was to formatively evaluate the BEACON implementation. In this article, we explore the processes and pathways by which changes in provider behaviour were achieved in the BEACON study and identify factors that facilitated or hindered uptake of the educational invention by providers and staff. | USA | Unspecified healthcare professionals. 14 participants. Individual interviews | Educational materials; Conferences, courses, workshops; Guidelines, toolkits, policies, tech tools | Qualitative data analysis revealed processes that facilitated or impeded uptake of the intervention. Results will be used to inform ongoing and future clinical initiatives and optimize future implementation of education-based interventions to improve adoption of best practices for end-of-life care within acute care settings. |
| Virani et al (2014)  Quantitative  Unspecified design | The purpose of this article was to describe the implementation and evaluation of the End-of-Life Nursing Education Consortium for Public Hospitals (ELNEC-PH), a comprehensive, state-wide initiative to enhance palliative care education and leadership in 16 (out of 17) California P/SN hospitals. | USA | Nurses. 58 participants. Surveys; Existing register data; Unspecified | Conferences, courses, workshops; Other social interaction strategies | At 18 months after course, participants reported that the program significantly increased their effectiveness and ability to teach palliative care content to their colleagues (P= .028). There was a significant increase in the number of educational programs for all modules offered across all 16 hospitals (P G .05). Lessons learned included developing a team, aligning the palliative care program with the goals and mission of the organization, and identifying key stakeholders and their needs. The ELNEC-PH project has been an invaluable educational effort that has attempted to address the growing need for palliative care education in California P/SN hospitals, which can be a model for other P/SN hospitals nationally. |
| Woo et al (2011)   Quantitative  Quasi-experimental | Objectives: The burden of suffering among patients with end-stage chronic diseases may be greater than those of cancer patients, as a result of longer duration of illness trajectory and high prevalence of symptoms, yet they may be less likely to receive palliative care services. To improve the quality of care of these patients, we carried out a continuous quality improvement initiative among medical and nursing staff of a convalescent facility. | China | Patients. 169 participants. Surveys; Individual interviews; | Educational materials; Conferences, courses, workshops; Other social interaction strategies; Guidelines, toolkits, policies, tech tools; Other organizational strategies | There were 80 and 89 participants in the pre- and post-intervention phase. The initiative resulted in shorter duration of stay, fewer investigations, fewer transfers back to the afﬁliated acute care hospital, and more follow-up by the outreach team, with no signiﬁcant difference in mortality after adjusting for age and comorbidity. Symptoms of pain and cough were reduced, while there was a trend toward more constipation but less dizziness. Family members’ satisfaction improved. |
| Yamamoto et al (2015)  Quantitative  Unspecified design | Palliative care is an essential part of medicine, but most physicians have had no formal opportunity to acquire basic skills in palliative care. In Japan, the Palliative care Emphasis program on symptom management and Assessment for Continuous Medical Education (PEACE) was launched to provide formal primary palliative care education for all physicians engaged in cancer care. This study sought to determine whether PEACE could improve physicians’ knowledge of, practices in, and difﬁculties with palliative care. | Japan | Physicians. 85 participants. Surveys | Conferences, courses, workshops | Among 223 physicians participating in the program, 85 (38%) answered the follow-up survey. Signiﬁcant improvements were noted on the PEACE-Q compared with baseline immediately after completion of the program, and this progress was maintained at 2 months (21.7 – 5.56 versus 29.5 – 2.10 versus 28.7 – 3.28, respectively; p < 0.0001). Similarly, signiﬁcant improvements were noted for total scores on both the PCPS and the PCDS at 2 months after completion of the program (62.1 – 13.9 versus 69.6 – 9.94 [p < 0.0001] for the PCPS; 44.4 – 9.96 versus 39.4 – 10.7 [p < 0.0001] for the PCDS). |
| Yoshioka et al (2014)  Quantitative  Quasi-experimental | This study aimed to examine effectiveness of the End-of-life nursing care continuing education program for general ward nurses. | Japan | Nurses. 22 participants. Surveys | Educational materials; Conferences, courses, workshops; Reminders or triggers, check-lists, templates | The primary outcome, implementation ability of end-of-life nursing care, was significantly improved after the program; improvements continued even at 2 months after. Similar results were obtained for nurses’ confidence and knowledge concerning end-of-life nursing care. As for attitude toward end-of-life care, participants’ scores were further elevated after the program. The participants rated the usefulness of the program as high. The effectiveness of the program was suggested from these results. |

Supplementary File 3. References to the included studies (n=183)

Albizu-Rivera, A., Portman, D. G., Thirlwell, S., Codada, S. N., & Donovan, K. A. (2016). Implementation of NCCN Palliative Care Guidelines by member institutions. Support Care Cancer 24(2), 929-932.

Arenella, C., Finke, B., Domer, T., Kaur, J. S., Merriman, M. P., & Ousley, A. (2010). Adaptation, dissemination, and evaluation of a cancer palliative care curriculum for the Indian health system. Journal of palliative care, 26(1), 15-21.

Arenella, C., Yox, S., Eckstein, D. S., & Ousley, A. (2010). Expanding the reach of a cancer palliative care curriculum through Web-based dissemination: a public-private collaboration. Journal of Cancer Education, 25(3), 418-421.

Armstrong, B., Jenigiri, B., Hutson, S. P., Wachs, P. M., & Lambe, C. E. (2013). The impact of a palliative care program in a rural appalachian community hospital: A quality improvement process. American Journal of Hospice & Palliative Medicine, 30(4), 380-387.

Badger, F., Plumridge, G., Hewison, A., Shaw, K. L., Thomas, K., & Clifford, C. (2012). An evaluation of the impact of the Gold Standards Framework on collaboration in end-of-life care in nursing homes. A qualitative and quantitative evaluation. International Journal of Nursing Studies, 49(5), 586-595.

Bailey, F. A., Williams, B. R., Woodby, L. L., Goode, P. S., Redden, D. T., Houston, T. K., Granstaff, U. S., Johnson, T. M., Pennypacker, L. C., Haddock, K. S., Painter, J. M., Spencer, J. M., Hartney, T., & Burgio, K. L. (2014). Intervention to improve care at life's end in inpatient settings: The BEACON trial. Journal of General Internal Medicine, 29(6), 836-843.

Beck, I., Jakobsson, U., & Edberg, A. K. (2015). Applying a palliative care approach in residential care: Effects on nurse assistants' work situation. Palliative and Supportive Care, 13(3), 543-553.

Beck, I., Tornquist, A., & Edberg, A. K. (2014). Nurse assistants' experience of an intervention focused on a palliative care approach for older people in residential care. International Journal of Older People Nursing, 9(2), 140-150.

Beernaert, K., Smets, T., Cohen, J., Verhofstede, R., Costantini, M., Eecloo, K., Noortgate, N., & Deliens, L. (2017). Improving comfort around dying in elderly people: a cluster randomised controlled trial. Lancet (london, england), 390(10090), 125-134.

Bekelman, D., Hooker, S., Nowels, C., Main, D., Meek, P., McBryde, C., Hattler, B., Lorenz, K., & Heidenreich, P. (2014). Feasibility and acceptability of a collaborative care intervention to improve symptoms and quality of life in chronic heart failure: mixed methods pilot trial. Journal of Palliative Medicine, 17(2), 145-151.

Bergman, J., Lorenz, K. A., Ballon-Landa, E., Kwan, L., Lerman, S. E., Saigal, C. S., Bennett, C. J., & Litwin, M. S. (2015). A Scalable Web-Based Module for Improving Surgical and Medical Practitioner Knowledge and Attitudes about Palliative and End-of-Life Care. Journal of Palliative Medicine, 18(5), 415-420.

Berkowitz, R. E., Jones, R. N., Rieder, R., Bryan, M., Schreiber, R., Verney, S., & Paasche-Orlow, M. K. (2011). Improving disposition outcomes for patients in a geriatric skilled nursing facility. Journal of the American Geriatrics Society, 59(6), 1130-1136.

Bernacki, R. E., Ko, D. N., Higgins, P., Whitlock, S. N., Cullinan, A., Wilson, R., Jackson, V., Dahlin, C., Abrahm, J., Mort, E., Scheer, K. N., Block, S., & Billings, J. A. (2012). Improving access to palliative care through an innovative quality improvement initiative: An opportunity for pay-for-performance Journal of Palliative Medicine, 15(2), 192-200.

Beyea, A., Fischer, J., Schenck, A., & Hanson, L. C. (2013). Integrating palliative care information and hospice referral in Medicaid primary care. Journal of Palliative Medicine, 16(4), 376-382.

Blackwell, R. W. n., Lowton, K., Robert, G., Grudzen, C., & Grocott, P. (2017). Using Experience-based Co-design with older patients, their families and staff to improve palliative care experiences in the Emergency Department: A reflective critique on the process and outcomes. International Journal of Nursing Studies, 68, 83-94.

Bokberg, C., Behm, L., Wallerstedt, B., & Ahlstrom, G. (2019). Evaluation of person-centeredness in nursing homes after a palliative care intervention: pre- and post-test experimental design. BMC Palliative Care, 18(1), 44.

Bove, D. G., Lavesen, M., Jellington, M. O., Marsaa, K. B., & Herling, S. F. (2018). First year experiences with a palliative out-patients structure for patients with COPD: a qualitative study of health professionals' expectations and experiences. BMC Palliative Care, 17(1), 113.

Bradley, C., Weaver, J., & Brasel, K. (2010). Addressing access to palliative care services in the surgical intensive care unit. Surgery, 147(6), 871-877.

Bristowe, K., Carey, I., Hopper, A., Shouls, S., Prentice, W., Higginson, I. J., & Koffman, J. (2018). Seeing is believing – healthcare professionals’ perceptions of a complex intervention to improve care towards the end of life: A qualitative interview study. Palliative Medicine, 32(2), 525-532.

Brousseau, R. T., Jameson, W., Kalanj, B., Kerr, K., O'Malley, K., & Pantilat, S. (2012). A multifaceted approach to spreading palliative care consultation services in California public hospital systems. Journal for healthcare quality: official publication of the National Association for Healthcare Quality, 34(2), 77-85.

Brown, K. L., & Ashcraft, A. S. (2019). Comfort or Care: Why Do We Have to Choose? Implementing a Geriatric Trauma Palliative Care Program. Journal of Trauma Nursing, 26(1), 2-9.

Brown-Saltzman, K., Upadhya, D., Larner, L., & Wenger, N. S. (2010). An intervention to improve respiratory therapists' comfort with end-of-life care. Respiratory Care, 55(7), 858-865.

Calvel, L., Blondet, L. V., Chedotal, I., Grosshans, D., Lefebvre, F., Mounier, G., Mangin, E., & Kopferschmitt, M. C. (2019). Difficulties in providing palliative care in identified palliative care beds: An exploratory survey. la Presse Medicale, 48(7), e209-e215.

Campion-Smith, C., Austin, H., Criswick, S., Dowling, B., & Francis, G. (2011). Can sharing stories change practice? A qualitative study of an interprofessional narrative-based palliative care course. Journal of Interprofessional Care, 25(2), 105-111.

Carey, I., Shouls, S., Bristowe, K., Morris, M., Briant, L., Robinson, C., Caulkin, R., Griffiths, M., Clark, K., Koffman, J., & Hopper, A. (2015). Improving care for patients whose recovery is uncertain. The AMBER care bundle: design and implementation. BMJ Supportive & Palliative Care, 5(4), 405-411.

Centeno, C., Garralda, E., Carrasco, J. M., Den Herder-Van Der Eerden, M., Aldridge, M., Stevenson, D., Meier, D. E., & Hasselaar, J. (2017). The Palliative Care Challenge: Analysis of Barriers and Opportunities to Integrate Palliative Care in Europe in the View of National Associations. Journal of Palliative Medicine, 20(11), 1195-1204.

Chan, C. W., Chui, Y. Y., Chair, S. Y., Sham, M. M., Lo, R. S., Ng, C. S., Chan, H. Y., & Lai, D. C. (2014). The evaluation of a palliative care programme for people suffering from life-limiting diseases. Journal of Clinical Nursing, 23(1), 113-123.

Chi-Yin, K., Wen-Yu, H., Tai-Yuan, C., & Ching-Yu, C. (2014). Effects of the hospital-based palliative care team on the care for cancer patients: An evaluation study. International Journal of Nursing Studies, 51(2), 226-235.

Childers, J. W., & Arnold, R. M. (2018). Expanding Goals of Care Conversations Across a Health System: The Mapping the Future Program. Journal of Pain and Symptom Management, 56(4), 637-644.

Clark, J., Marshall, B., Sheward, K., & Allan, S. (2012). Staff perceptions of the impact of the Liverpool Care Pathway in aged residential care in New Zealand. International Journal of Palliative Nursing, 18(4), 171-178.

Clark, J. B., Sheward, K., Marshall, B., & Allan, S. G. (2012). Staff perceptions of end-of-life care following implementation of the liverpool care pathway for the dying patient in the acute care setting: A New Zealand perspective. Journal of Palliative Medicine, 15(4), 468-473.

Clark, K., Curry, T., & Byfieldt, N. (2015). The effect of a care bundle on nursing staff when caring for the dying. International Journal of Palliative Nursing, 21(8), 392-398.

Clark, K., Willis, A., & Byfieldt, N. (2017). An Observational Study to Explore the Feasibility of Assessing Bereaved Relatives’ Experiences Before and After a Quality Improvement Project to Improve Care of Dying Medical Inpatients. American Journal of Hospice and Palliative Medicine, 34(3), 263-268.

Collins, K. A., Hughes, P. M., Ibbotson, R., Foy, G., & Brooks, D. (2016). Views and experiences of using integrated care pathways (ICPs) for caring for people in the last days to hours of life: results from a cross-sectional survey of UK professionals. BMJ Support Palliat Care, 6(3), 377-380.

Corcoran, K. (2016). Evaluation of an educational workshop to increase comfort levels of professional caregivers with end-of-life care. MEDSURG Nursing, 25(2), 103-109.

Cornetta, K., Kipsang, S., Gramelspacher, G., Choi, E., Brown, C., Hill, A. B., Loehrer, P. J., Busakhala, N., & Chite Asirwa, F. (2015). Integration of Palliative Care Into Comprehensive Cancer Treatment at Moi Teaching and Referral Hospital in Western Kenya. Journal of Global Oncology, 1(1), 23-29.

Costantini, M., Pellegrini, F., Di Leo, S., Beccaro, M., Rossi, C., Flego, G., Romoli, V., Giannotti, M., Morone, P., Ivaldi, G. P., Cavallo, L., Fusco, F., & Higginson, I. J. (2014). The Liverpool Care Pathway for cancer patients dying in hospital medical wards: A before–after cluster phase II trial of outcomes reported by family members. Palliative Medicine, 28(1), 10-17.

Costantini, M., Romoli, V., Leo, S., Beccaro, M., Bono, L., Pilastri, P., Miccinesi, G., Valenti, D., Peruselli, C., Bulli, F., Franceschini, C., Grubich, S., Brunelli, C., Martini, C., Pellegrini, F., & Higginson, I. (2014). Liverpool Care Pathway for patients with cancer in hospital: a cluster randomised trial. Lancet (london, england), 383(9913), 226-237.

Cox, A., Arber, A., Bailey, F., Dargan, S., Gannon, C., Lisk, R., Quinn, B., Samarasinghe, J., Wrigley, M., & Gallagher, A. (2017). Developing, implementing and evaluating an end of life care intervention. Nursing Older People, 29(1), 27-35.

Creutzfeldt, C. J., Engelberg, R. A., Healey, L., Cheever, C. S., Becker, K. J., Holloway, R. G., & Curtis, J. R. (2015). Palliative care needs in the neuro-icu. Critical Care Medicine, 43(8), 1677-1684.

Cronfalk, B. S., Ternestedt, B. M., Larsson, L. L., Henriksen, E., Norberg, A., & Osterlind, J. (2015). Utilization of palliative care principles in nursing home care: Educational interventions. Palliative & Supportive Care, 13(6), 1745-1753.

Cross, H., Cameron, M., Marsh, S., & Tuffrey-Wijne, I. (2012). Practical approaches toward improving end-of-life care for people with intellectual disabilities: Effectiveness and sustainability. Journal of Palliative Medicine, 15(3), 322-326.

Curtis, J., Nielsen, E., Treece, P., Downey, L., Dotolo, D., Shannon, S., Back, A., Rubenfeld, G., & Engelberg, R. (2011). Effect of a quality-improvement intervention on end-of-life care in the intensive care unit: a randomized trial. American Journal of Respiratory and Critical Care Medicine, 183(3), 348-355.

DeMiglio, L., & Williams, A. (2012). Factors enabling shared care with primary healthcare providers in community settings: the experiences of interdisciplinary palliative care teams. Journal of Palliative Care 28(4), 282-289.

Di Leo, S., Beccaro, M., Finelli, S., Borreani, C., & Costantini, M. (2011). Expectations about and impact of the Liverpool Care Pathway for the dying patient in an Italian hospital Palliative Medicine, 25(4), 293-303.

Di Leo, S., Romoli, V., Higginson, I. J., Bulli, F., Fantini, S., Sguazzotti, E., & Costantini, M. (2015). 'Less ticking the boxes, more providing support': A qualitative study on health professionals' concerns towards the Liverpool Care of the Dying Pathway. Palliative Medicine, 29(6), 529-537.

DiMartino, L. D., Birken, S. A., Hanson, L. C., Trogdon, J. G., Clary, A. S., Weinberger, M., Reeder-Hayes, K., & Weiner, B. J. (2018). The influence of formal and informal policies and practices on health care innovation implementation: A mixed-methods analysis Health Care Management Review, 43(3), 249.

DiMartino, L. D., Weiner, B. J., Hanson, L. C., Weinberger, M., Birken, S. A., Reeder-Hayes, K., & Trogdon, J. G. (2019). The impact of two triggered palliative care consultation approaches on consult implementation in oncology. Healthcare: The Journal of Delivery Science and Innovation, 7(1), 38-43.

Downing, J., Batuli, M., Kivumbi, G., Kabahweza, J., Grant, L., Murray, S. A., Namukwaya, E., & Leng, M. (2016). A palliative care link nurse programme in Mulago Hospital, Uganda: An evaluation using mixed methods. BMC Palliative Care, 15(1), 40.

Ellis-Smith, C., Higginson, I. J., Daveson, B. A., Henson, L. A., & Evans, C. J. (2018). How can a measure improve assessment and management of symptoms and concerns for people with dementia in care homes? A mixed-methods feasibility and process evaluation of IPOS-Dem. PLoS One, 13(7), e0200240.

Ersek, M., Hickman, S. E., Thomas, A. C., Bernard, B., & Unroe, K. T. (2018). Stakeholder Perspectives on the Optimizing Patient Transfers, Impacting Medical Quality, and Improving Symptoms: Transforming Institutional Care (OPTIMISTIC) Project. The Gerontologist, 58(6), 1177-1187.

Ersek, M., Sebego, M., Bloom, A. M., Shaibu, S., McMenamin, E., & Mokotedi, M. (2010). Development and evaluation of an international, interdisciplinary palliative care workshop in Botswana. International Journal of Palliative Nursing, 16(1), 13-21.

Evans, J. M., Mackinnon, M., Pereira, J., Earle, C. C., Gagnon, B., Arthurs, E., Gradin, S., Buchman, S., & Wright, F. C. (2019). Integrating early palliative care into routine practice for patients with cancer: A mixed methods evaluation of the INTEGRATE Project. Psychooncology, 28(6), 1261-1268.

Fedel, P., Joosse, L. L., & Jeske, L. (2014). Use of the Palliative Performance Scale version 2 in obtaining palliative care consults. Journal of Clinical Nursing, 23(13), 2012-2021.

Fernandes, R., Riklon, S., Langidrik, J. R., Williams, S. N., & Kabua, N. (2014). Collaboration between a US Academic Institution and International Ministry of Health to develop a culturally appropriate palliative care navigation curriculum. Healthcare: The Journal of Delivery Science and Innovation, 2(4), 275-279.

Finkelstein, M., Goldstein, N. E., Horton, J. R., Eshak, D., Lee, E. J., & Kohli-Seth, R. (2016). Developing triggers for the surgical intensive care unit for palliative care integration. Journal of Critical Care, 35, 7-11.

Finucane, A. M., Stevenson, B., Moyes, R., Oxenham, D., & Murray, S. A. (2013). Improving end-of-life care in nursing homes: Implementation and evaluation of an intervention to sustain quality of care. Palliative Medicine, 27(8), 772-778.

Frank, C., Touw, M., Suurdt, J., Jiang, X., Wattam, P., & Heyland, D. K. (2012). Optimizing end-of-life care on medical clinical teaching units using the CANHELP Questionnaire and a Nurse Facilitator: A feasibility study. Canadian Journal of Nursing Research, 44(1), 40-58.

Frendak, L. S., Wright, S. M., & Wu, D. S. (2019). The Effect of a Standardized Triage Process on Efficiency and Productivity of an Inpatient Palliative Care Team. American Journal of Hospice and Palliative Medicine, 37(6), 413-417.

Friedrichsen, M., Hajradinovic, Y., Jakobsson, M., Milberg, P., & Milberg, A. (2016). Palliative care consultation team on acute wards—an intervention study with pre-post comparisons. Supportive care in cancer, 25(2), 371-380.

Gaertner, J., Wolf, J., Scheicht, D., Frechen, S., Klein, U., Hellmich, M., Ostgathe, C., Hallek, M., & Voltz, R. (2010). Implementing WHO recommendations for palliative care into routine lung cancer therapy: a feasibility project. Journal of Palliative Medicine, 13(6), 727-732.

Gilbert, J. E., Howell, D., King, S., Sawka, C., Hughes, E., Angus, H., & Dudgeon, D. (2012). Quality improvement in cancer symptom assessment and control: the Provincial Palliative Care Integration Project (PPCIP). Journal of Pain and Symptom Management, 43(4), 663-678.

Gillett, K., & Bryan, L. (2016). ‘Quality End of Life Care for All’ (QELCA): the national rollout of an end-of-life workforce development initiative. BMJ supportive & palliative care, 6(2), 225-230.

Glajchen, M., Lawson, R., Homel, P., Desandre, P., & Todd, K. H. (2011). A rapid two-stage screening protocol for palliative care in the emergency department: A quality improvement initiative. Journal of Pain and Symptom Management, 42(5), 657-662.

Glare, P., Plakovic, K., Schloms, A., Egan, B., Epstein, A. S., Kelsen, D., & Saltz, L. (2013). Study using the NCCN guidelines for palliative care to screen patients for palliative care needs and referral to palliative care specialists. Journal of the National Comprehensive Cancer Network, 11(9), 1087-1096.

Golden, A. G., Antoni, C., & Gammonley, D. (2016). A home-based palliative care consult service for Veterans. American Journal of Hospice & Palliative Medicine, 33(9), 858-862.

Gradwohl, K., Wood, G. J., Clepp, R. K., Rivnay, L., & Szmuilowicz, E. (2019). Preventing Readmissions Through Effective Partnerships-Communication and Palliative Care (PREP-CPC): A Multisite Intervention for Encouraging Goals of Care Conversations for Hospitalized Patients Facing Serious Illness. American Journal of Hospice & Palliative Medicine, 37(8), 582-588. DOI: 10.1177/1049909119891996

Grainger, M. N., Hegarty, S., Schofield, P., White, V., & Jefford, M. (2010). Discussing the transition to palliative care: evaluation of a brief communication skills training program for oncology clinicians. Palliative & Supportive Care, 8(4), 441-447.

Grant, L., Downing, J., Luyirika, E., Murphy, M., Namukwaya, L., Kiyange, F., Atieno, M., Kemigisha-Ssali, E., Hunt, J., Snell, K., Murray, S. A., & Leng, M. (2017). Integrating palliative care into national health systems in Africa: a multi-country intervention study. Journal of Global Health, 7(1), 010419.

Hahne, P., Lundström, S., Leveälahti, H., Winnhed, J., & Öhlén, J. (2017). Changes in professionals' beliefs following a palliative care implementation programme at a surgical department: A qualitative evaluation. BMC palliative care, 16(1), 77-77.

Hall, K. L., Rafalson, L., Mariano, K., & Michalek, A. (2016). Evaluation of Hospital-Based Palliative Care Programs. American journal of hospice & palliative medicine, 33(1), 77-83.

Hall, S., Goddard, C., Stewart, F., & Higginson, I. J. (2011). Implementing a quality improvement programme in palliative care in care homes: A qualitative study. BMC geriatrics, 11(1), 31-31.

Hanson, L. C., Collichio, F., Bernard, S. A., Wood, W. A., Milowsky, M., Burgess, E., . . . Lin, F.-C. (2017). Integrating Palliative and Oncology Care for Patients with Advanced Cancer: A Quality Improvement Intervention. Journal of palliative medicine, 20(12), 1366-1371.

Hanson, L. C., Zimmerman, S., Song, M.-K., Lin, F.-C., Rosemond, C., Carey, T. S., & Mitchell, S. L. (2017). Effect of the Goals of Care Intervention for Advanced Dementia: A Randomized Clinical Trial. JAMA internal medicine, 177(1), 24-31.

Harding, R., Simms, V., Alexander, C., Collins, K., Combo, E., Memiah, P., . . . Loy, G. (2013). Can palliative care integrated within HIV outpatient settings improve pain and symptom control in a low-income country? A prospective, longitudinal, controlled intervention evaluation. AIDS care, 25(7), 795-804.

Hauser, J. M., Preodor, M., Roman, E., Jarvis, D. M., & Emanuel, L. (2015). The Evolution and Dissemination of the Education in Palliative and End-of-Life Care Program. Journal of palliative medicine, 18(9), 765-770.

Haydar, S. A., Almeder, L., Michalakes, L., Han, P. K. J., & Strout, T. D. (2017). Using the Surprise Question To Identify Those with Unmet Palliative Care Needs in Emergency and Inpatient Settings: What Do Clinicians Think? Journal of palliative medicine, 20(7), 729-735.

Head, B. A., LaJoie, S., Augustine-Smith, L., Cantrell, M., Hofmann, D., Keeney, C., & Pfeifer, M. (2010). Palliative care case management: increasing access to community-based palliative care for medicaid recipients. Professional case management, 15(4), 206-217.

Herce, M. E., Elmore, S. N., Kalanga, N., Keck, J. W., Wroe, E. B., Phiri, A., . . . Rigodon, J. (2014). Assessing and responding to palliative care needs in rural sub-Saharan Africa: Results from a model intervention and situation analysis in Malawi. PloS one, 9(10), e110457-e110457.

Higginson, I. J., Koffman, J., Hopkins, P., Prentice, W., Burman, R., Leonard, S., . . . Shipman, C. (2013). Development and evaluation of the feasibility and effects on staff, patients, and families of a new tool, the Psychosocial Assessment and Communication Evaluation (PACE), to improve communication and palliative care in intensive care and during clinical uncertainty. BMC medicine, 11(1), 213-213.

Ho, A. H. Y., Luk, J. K. H., Chan, F. H. W., Chun Ng, W., Kwok, C. K. K., Yuen, J. H. L., . . . Chan, C. L. W. (2016). Dignified Palliative Long-Term Care: An Interpretive Systemic Framework of End-of-Life Integrated Care Pathway for Terminally Ill Chinese Older Adults. American journal of hospice & palliative medicine, 33(5), 439-447.

Ho, J. K.-M. (2016). Resuscitation versus end-of-life care: Exploring the obstacles and supportive behaviors to providing end-of-life care as perceived by emergency nurses after implementing the end-of-life care pathway. Applied nursing research, 29, e7-e13.

Hockley, J. (2014). Learning, support and communication for staff in care homes: outcomes of reflective debriefing groups in two care homes to enhance end-of-life care. International journal of older people nursing, 9(2), 118-130.

Hockley, J., & Kinley, J. (2016). A practice development initiative supporting care home staff deliver high quality end-of-life care. International journal of palliative nursing, 22(10), 474-481.

Hockley, J., Watson, J., Oxenham, D., & Murray, S. A. (2010). The integrated implementation of two end-of-life care tools in nursing care homes in the UK: an in-depth evaluation. Palliative medicine, 24(8), 828-838.

Holdsworth, L. M. (2019). Conceptualizing “project resiliency”: A qualitative study exploring the implementation of coordinated care within a context of system change. Journal of Integrated Care, 27(2), 163-172.

Hopkins, B., Gold, M., Wei, A., & Grigoriadis, G. (2017). Improving the Transition to Palliative Care for Patients With Acute Leukemia: A Coordinated Care Approach. Cancer nursing, 40(3), E17-E23.

Horey, D. E., Street, A. F., & Sands, A. F. (2012). Acceptability and feasibility of end-of-life care pathways in Australian residential aged care facilities. Medical journal of Australia, 197(2), 106-109.

Hsu-Kim, C., Friedman, T., Gracely, E., & Gasperino, J. (2015). Integrating Palliative Care into Critical Care: A Quality Improvement Study. Journal of intensive care medicine, 30(6), 358-364.

Hudson, B. E., Ameneshoa, K., Gopfert, A., Goddard, R., Forbes, K., Verne, J., . . . McCune, C. A. (2017). Integration of palliative and supportive care in the management of advanced liver disease: development and evaluation of a prognostic screening tool and supportive care intervention. Frontline gastroenterology, 8(1), 45-52.

Hurst, E., Yessayan, L., Mendez, M., Hammad, A., & Jennings, J. (2018). Preliminary Analysis of a Modified Screening Tool to Increase the Frequency of Palliative Care Consults. American journal of hospice & palliative medicine, 35(3), 417-422.

Hussainy, S. Y., Marriott, J. L., Beattie, J., Nation, R. L., & Dooley, M. J. (2010). A palliative cancer care flexible education program for australian community pharmacists. American journal of pharmaceutical education, 74(2), 24-24.

Hydeman, J. (2013). Improving the Integration of Palliative Care in a Comprehensive Oncology Center: Increasing Primary Care Referrals to Palliative Care. Omega: Journal of Death and Dying, 67(1-2), 127-134.

Iliffe, S., Davies, N., Manthorpe, J., Crome, P., Ahmedzai, S. H., Vernooij-Dassen, M. J. F. J., & Engels, Y. (2016). Improving palliative care in selected settings in England using quality indicators: a realist evaluation. BMC palliative care, 15(1), 69.

Imura, C. R. N., Morita, T. M. D., Kato, M. M. D., Akizuki, N. M. D. P., Kinoshita, H. M. D., Shirahige, Y. M. D. P., . . . Eguchi, K. M. D. P. (2014). How and Why Did a Regional Palliative Care Program Lead to Changes in a Region? A Qualitative Analysis of the Japan OPTIM Study. Journal of pain and symptom management, 47(5), 849-859.

Jack, B. A., Kirton, J., Birakurataki, J., & Merriman, A. (2011). ‘A bridge to the hospice’: The impact of a Community Volunteer Programme in Uganda. Palliative medicine, 25(7), 706-715.

Jenko, M., Adams, J. A., Johnson, C. M., Thompson, J. A., & Bailey, D. E. (2015). Facilitating Palliative Care Referrals in the Intensive Care Unit: A Pilot Project. Dimensions of critical care nursing, 34(6), 329-339.

Kadlec, H., Hollander, M., Clelland, C., Kallstrom, L., & Hollander, M. (2015). Family physicians enhance end-of-life care: Evaluation of a new continuing medical education learning module in British Columbia. BMC medical education, 15(1), 119-119.

Karim, S., Harle, I., O'Donnell, J., Li, S., & Booth, C. M. (2018). Documenting Goals of Care Among Patients With Advanced Cancer: Results of a Quality Improvement Initiative. Journal of Oncology Practice, 14(9), e557-e565.

Karlekar, M. B., Maxwell, C. A., Dietrich, M. S., & Miller, R. S. (2017). Creating new opportunities to educate families on the impact of frailty and cognitive impairment in a trauma intensive care unit: Results of a quality improvement project. Journal of Palliative Medicine, 20(2), 193-196.

Kelly, K., Thrane, S., Virani, R., Malloy, P., & Ferrell, B. (2011). Expanding palliative care nursing education in California: the ELNEC Geriatric project. International Journal of Palliative Nursing, 17(4), 188-194.

Kim, B. H., Kim, H. S., Yu, S. J., Choi, S., Jung, Y., & Kwon, S. H. (2012). Evaluation of End-of-Life Nursing Education Consortium-Geriatric Train-the-Trainer Program in Korea. Korean Journal of Adult Nursing, 24(4), 390-397.

Kinley, J., Denton, L., & Scott, S. (2018). Development and implementation of the Steps to Successful Palliative Care programme in residential care homes for people with a learning disability. International Journal of Palliative Nursing, 24(10), 492-502.

Kinley, J., Preston, N., & Froggatt, K. (2018). Facilitation of an end-of-life care programme into practice within UK nursing care homes: A mixed-methods study. International Journal of Nursing Studies, 82, 1-10.

Kinley, J., Stone, L., Butt, A., Kenyon, B., & Lopes, N. S. (2017). Developing, implementing and sustaining an end-of-life care programme in residential care homes. International journal of palliative nursing, 23(4), 186-193.

Kinley, J., Stone, L., Dewey, M., Levy, J., Stewart, R., McCrone, P., . . . Hockley, J. (2014). The effect of using high facilitation when implementing the Gold Standards Framework in Care Homes programme: A cluster randomised controlled trial. Palliative medicine, 28(9), 1099-1109.

Kluger, B. M., Persenaire, M. J., Holden, S. K., Palmer, L. T., Redwine, H. M., Berk, J., . . . Carter, J. (2018). Implementation issues relevant to outpatient neurology palliative care. Annals of palliative medicine, 7(3), 339-348.

Koper, I., Pasman, H. R. W., Van der Plas, A. G. M., Schweitzer, B. P. M., & Onwuteaka-Philipsen, B. D. (2019). The association between PaTz and improved palliative care in the primary care setting: a cross-sectional survey. BMC Fam Pract, 20(1), 112.

Kortes-Miller, K., Jones-Bonofiglio, K., Hendrickson, S., & Kelley, M. L. (2016). Dying With Carolyn: Using Simulation to Improve Communication Skills of Unregulated Care Providers Working in Long-Term Care. Journal of applied gerontology, 35(12), 1259-1278.

Kramer, B. J., Cleary, J. F., & Mahoney, J. E. (2014). Enhancing Palliative Care for Low-Income Elders with Chronic Disease: Feasibility of a Hospice Consultation Model. Journal of social work in end-of-life & palliative care, 10(4), 356-377.

Krumm, N., Larkin, P., Connolly, M., Rode, P., & Elsner, F. (2014). Improving dementia care in nursing homes: experiences with a palliative care symptom-assessment tool (MIDOS). International journal of palliative nursing, 20(4), 187-192.

Kuhn, D. R., & Forrest, J. M. (2012). Palliative Care for Advanced Dementia: A Pilot Project in 2 Nursing Homes. American Journal of Alzheimer's Disease & Other Dementias, 27(1), 33-40.

Kuo, L. C., Lee, J. J., Cheung, D. S. T., Chen, P. J., & Lin, C. C. (2019). End-of-life care in cancer and dementia: a nationwide population-based study of palliative care policy changes. BMJ Supportive & Palliative Care. DOI: 10.1136/bmjspcare-2019-001782

Kurozumi, Y., Oishi, S., Sugano, Y., Sakashita, A., Kotooka, N., Suzuki, M., Higo, T., Yumino, D., Takada, Y., Maeda, S., Yamabe, S., Washida, K., Takahashi, T., Ohtani, T., Sakata, Y., & Sato, Y. (2019). Possible associations between palliative care conferences and positive outcomes when performing palliative care for patients with end-stage heart failure: a nationwide cross-sectional questionnaire survey. Heart Vessels, 34(3), 452-461.

Lau, C., Stilos, K., Nowell, A., Lau, F., Moore, J., & Wynnychuk, L. (2018). The comfort measures order set at a tertiary care academic hospital: Is there a comparable difference in end-of-life care between patients dying in acute care when CMOS is utilized? American Journal of Hospice & Palliative Medicine, 35(4), 652-663.

LeBaron, V. T., Bohnenkamp, S. K., & Reed, P. G. (2011). A community partnership approach to building and empowering a palliative care resource nurse team. Journal of Hospice and Palliative Nursing, 13(1), 31-40.

Lee, J., Cheng, J., Au, K. M., Yeung, F., Leung, M. T., Ng, J., Hui, E., Lo, R., & Woo, J. (2013). Improving the quality of end-of-life care in long-term care institutions. Journal of Palliative Medicine, 16(10), 1268-1274.

Lesperance, M., Shannon, R., Pumphrey, P. K., Dunbar, E., Genther, R., Coleman, C. L., Tabano, M., Maurer, J., Vazquez, A., Capp, E., McMillan, J., Wilkerson, K., Robbins, G., Phillips, D. G., Howick, P., Solaun, C., Sloan, J., & Colon-Otero, G. (2014). Training mid-level providers on palliative care: bringing advanced directives and symptom assessment and management to community oncology practices. American Journal of Hospice & Palliative Medicine, 31(3), 237-243.

Liang, Z., Dinella, J., Ren, D., Tuite, P. K., & Usher, B. M. (2016). Evaluation of a peer nurse coach quality improvement project on new nurse hire attitudes toward care for the dying. Journal of Hospice and Palliative Nursing, 18(5), 398-404.

Lin, C. Y., Arnold, R. M., Lave, J. R., Angus, D. C., & Barnato, A. E. (2010). Acute care practices relevant to quality end-of-life care: A survey of Pennsylvania hospitals. Quality and Safety in Health Care, 19(6), E12.

Lind, S., Sandberg, J., Brytting, T., Furst, C. J., & Wallin, L. (2018). Implementation of the integrated palliative care outcome scale in acute care settings - a feasibility study. Palliative & Supportive Care, 16(6), 698-705.

Lind, S., Wallin, L., Brytting, T., Furst, C. J., & Sandberg, J. (2017). Implementation of national palliative care guidelines in Swedish acute care hospitals: A qualitative content analysis of stakeholders' perceptions. Health Policy, 121(11), 1194-1201.

Livingston, G., Lewis-Holmes, E., Pitfield, C., Manela, M., Chan, D., Constant, E., Jacobs, H., Wills, G., Carson, N., & Morris, J. (2013). Improving the end-of-life for people with dementia living in a care home: An intervention study. International Psychogeriatrics, 25(11), 1849-1858.

Luckett, T., Chenoweth, L., Phillips, J., Brooks, D., Cook, J., Mitchell, G., Pond, D., Davidson, P. M., Beattie, E., Luscombe, G., Goodall, S., Fischer, T., & Agar, M. (2017). A facilitated approach to family case conferencing for people with advanced dementia living in nursing homes: perceptions of palliative care planning coordinators and other health professionals in the IDEAL study. International Psychogeriatrics, 29(10), 1713-1722.

Mackenzie, T., Innes, J., Boyd, M., Keane, B., Boxall, J., & Allan, S. (2011). Evaluating the role and value of a national office to coordinate Liverpool Care Pathway implementation in New Zealand. International Journal of Evidence-Based Healthcare, 9(3), 252-260.

Maeda, I., Tsuneto, S., Miyashita, M., Morita, T., Umeda, M., Motoyama, M., Kosako, F., Hama, Y., Kizawa, Y., Sasahara, T., & Eguchi, K. (2014). Progressive development and enhancement of palliative care services in Japan: nationwide surveys of designated cancer care hospitals for three consecutive years. Journal of Pain & Symptom Management, 48(3), 364-373.

Mason, B., Buckingham, S., Finucane, A., Hutchison, P., Kendall, M., McCutcheon, H., Porteous, L., & Murray, S. A. (2015). Improving primary palliative care in Scotland: Lessons from a mixed methods study. BMC Family Practice, 16(1), 176.

McConnell, T., O'Halloran, P., Donnelly, M., & Porter, S. (2015). Factors affecting the successful implementation and sustainability of the Liverpool Care Pathway for dying patients: A realist evaluation. BMJ Supportive and Palliative Care, 5(1), 70-77.

McGrath, L. S., Foote, D. G., Frith, K. H., & Hall, W. M. (2013). Cost effectiveness of a palliative care program in a rural community hospital. Nursing Economics, 31(4), 176-183.

Moore, K. J., Candy, B., Davis, S., Gola, A., Harrington, J., Kupeli, N., Vickerstaff, V., King, M., Leavey, G., Nazareth, I., Omar, R. Z., Jones, L., & Sampson, E. L. (2017). Implementing the compassion intervention, a model for integrated care for people with advanced dementia towards the end of life in nursing homes: a naturalistic feasibility study. BMJ Open, 7(6), e015515.

Mun, E., Ceria-Ulep, C., Umbarger, L., & Nakatsuka, C. (2016). Trend of Decreased Length of Stay in the Intensive Care Unit (ICU) and in the Hospital with Palliative Care Integration into the ICU. The Permanente journal, 20(4), 56-61.

Mun, E., Umbarger, L., Ceria-Ulep, C., & Nakatsuka, C. (2018). Palliative Care Processes Embedded in the ICU Workflow May Reserve Palliative Care Teams for Refractory Cases. American Journal of Hospice and Palliative Medicine, 35(1), 60-65.

Namisango, E., Ntege, C., Luyirika, E. B., Kiyange, F., & Allsop, M. J. (2016). Strengthening pharmaceutical systems for palliative care services in resource limited settings: piloting a mHealth application across a rural and urban setting in Uganda. BMC Palliat Care, 15(1), 20.

Neo, P. S., Poon, M. C., Peh, T. Y., Ong, S. Y., Koo, W. H., Santoso, U., Goh, C. R., & Yee, A. C. (2012). Improvements in end-of-life care with a protocol-based pathway for cancer patients dying in a Singapore hospital. Annals Academy of Medicine Singapore, 41(11), 483-493.

Nikbakht-Van de S, V., Ande, C. V., Braat, C., Visser, A. P., Delnoij, D. M., & van Staa, A. L. (2014). Why a carefully designed, nurse-led intervention failed to meet expectations: the case of the Care Programme for Palliative Radiotherapy. European Journal of Oncology Nursing, 18(2), 151-158.

Noble, C., Grealish, L., Teodorczuk, A., Shanahan, B., Hiremagular, B., Morris, J., & Yardley, S. (2018). How can end of life care excellence be normalized in hospitals? Lessons from a qualitative framework study. BMC Palliat Care, 17(1), 100.

Noome, M., Dijkstra, B., Leeuwen, E., & Vloet, L. (2017). Effectiveness of supporting intensive care units on implementing the guideline 'End-of-life care in the intensive care unit, nursing care': a cluster randomized controlled trial. Journal of advanced nursing, 73(6), 1339-1354.

Norton, S. A., Powers, B. A., Schmitt, M. H., Metzger, M., Fairbanks, E., DeLuca, J., & Quill, T. E. (2011). Navigating tensions: Integrating palliative care consultation services into an academic medical center setting. Journal of Pain and Symptom Management, 42(5), 680-690.

O'Brien, M., Kirton, J., Knighting, K., Roe, B., & Jack, B. (2016). Improving end of life care in care homes; An evaluation of the six steps to success programme. BMC Palliative Care, 15(1), 53.

Oosterveld-Vlug, M., Onwuteaka-Philipsen, B., Koppel, M. T., Hout, H. v., Smets, T., Pivodic, L., . . . Pasman, H. R. W. (2019). Evaluating the implementation of the PACE Steps to Success Programme in long-term care facilities in seven countries according to the RE-AIM framework. Implementation science, 14(1), 107-107.

Ouchi, K., Wu, M., Medairos, R., Grudzen, C. R., Balsells, H., Marcus, D., Whitson, M., Ahmad, D., Duprey, K., Mancherje, N., Bloch, H., Jaffrey, F., & Liberman, T. (2014). Initiating palliative care consults for advanced dementia patients in the emergency department. Journal of Palliative Medicine, 17(3), 346-350.

Pesut, B., Hooper, B. P., Robinson, C. A., Bottorff, J. L., Sawatzky, R., & Dalhuisen, M. (2015). Feasibility of a rural palliative supportive service. Rural and Remote Health, 15(2), 3116.

Porzio, G., Aielli, F., Verna, L., Martella, F., Aloisi, P., & Ficorella, C. (2013). Integrating oncology and palliative home care in Italy: The experience of the "l'Aquila per la Vita" Home Care Unit. Tumori, 99(2), 225-228.

Pype, P., Mertens, F., Wens, J., Stes, A., Eynden, B., & Deveugele, M. (2015). Preparing palliative home care nurses to act as facilitators for physicians' learning: evaluation of a training programme. Palliative Medicine, 29(5), 458-463.

Raijmakers, N., Dekkers, A., Galesloot, C., van Zuylen, L., & van der Heide, A. (2014). Barriers and facilitators to implementation of the Liverpool Care Pathway in the Netherlands: a qualitative study. BMJ Supportive & Palliative Care, 5(3), 259-265.

Rauenzahn, S. L., Schmidt, S., Aduba, I. O., Jones, J. T., Ali, N., & Tenner, L. L. (2017). Integrating Palliative Care Services in Ambulatory Oncology: An Application of the Edmonton Symptom Assessment System. Journal of Oncology Practice, 13(4), e401-e407.

Raunkiaer, M., & Timm, H. (2010). Development of palliative care in nursing homes: evaluation of a Danish project. International Journal of Palliative Nursing, 16(12), 613-620.

Reymond, L., Israel, F. J., & Charles, M. A. (2011). A residential aged care end-of-life care pathway (RAC EoLCP) for Australian aged care facilities. Australian Health Review 35(3), 350-356.

Rocque, G. B., Campbell, T. C., Johnson, S. K., King, J., Zander, M. R., Quale, R. M., Eickhoff, J. C., & Cleary, J. F. (2015). A Quantitative Study of Triggered Palliative Care Consultation for Hospitalized Patients With Advanced Cancer. Journal of Pain & Symptom Management, 50(4), 462-469.

Sánchez, J. M. S., Asensio, J. M. M., Gil, I. M. M., Sánchez, J. C. C., Tueba, E. P., & Dominguez, A. R. (2014). Impact of a legislative framework on quality of end-of-life care and dying in an acute hospital in Spain. International Journal of Palliative Nursing, 20(5), 225-231.

Schweitzer, B., Blankenstein, N., Slort, W., Knol, D. L., Deliens, L., & Van Der Horst, H. (2016). Writing information transfers for out-of-hours palliative care: A controlled trial among GPs. Scandinavian Journal of Primary Health Care, 34(2), 185-194.

Selman, L., Robinson, V., Klass, L., Khan, S., George, R., Shepherd, K., Burman, R., & Koffman, J. (2016). Improving confidence and competence of healthcare professionals in end-of-life care: An evaluation of the 'Transforming End of Life Care' course at an acute hospital trust. BMJ Supportive and Palliative Care, 6(2), 231-236.

Selvaggi, K. J., Vick, J. B., Jessell, S. A., Lister, J., Abrahm, J. L., & Bernacki, R. (2014). Bridging the gap: A palliative care consultation service in a hematological malignancy-bone marrow transplant unit. Journal of Community and Supportive Oncology, 12(2), 50-55.

Seow, H., Barbera, L., Howell, D., & Dy, S. M. (2010). Did Ontario's end-of-life care strategy reduce acute care service use? Healthcare quarterly, 13(1), 93-100.

Shah, K. G., Slough, T. L., Yeh, P. T., Gombwa, S., Kiromera, A., Oden, Z. M., & Richards-Kortum, R. R. (2013). Novel open-source electronic medical records system for palliative care in low-resource settings. BMC Palliative Care, 12(1), 31.

Singh, S., Rodriguez, A., Lee, D., Min, S. J., & Fischer, S. (2018). Usefulness of the Surprise Question on an Inpatient Oncology Service. American Journal of Hospice and Palliative Medicine, 35(11), 1421-1425.

Slort, W., Blankenstein, A. H., Schweitzer, B. P., Knol, D. L., Deliens, L., Aaronson, N. K., & van der Horst, H. E. (2013). Effectiveness of the ACA (Availability, Current issues and Anticipation) training programme on GP-patient communication in palliative care; a controlled trial. BMC Family Practice, 14(1), 93.

Slort, W., Blankenstein, A. H., Schweitzer, B. P., Knol, D. L., van der Horst, H. E., Aaronson, N. K., & Deliens, L. (2014). Effectiveness of the palliative care 'Availability, Current issues and Anticipation' (ACA) communication training programme for general practitioners on patient outcomes: a controlled trial. Palliative Medicine, 28(8), 1036-1045.

Smith, C. V., Maduro, R. S., Morgan, M. K., Ver Schneider, P., Rutledge, C. M., & Zimbro, K. S. (2019). Interdisciplinary Rounds on a Hospitalist Service: Impact on Palliative Care Measures, Quality, and Utilization Outcomes. Journal of Nursing Care Quality, 34(4), 295-300.

Tan, A., Seah, A., Chua, G., Lim, T. K., & Phua, J. (2014). Impact of a palliative care initiative on end-of-life care in the general wards: A before-and-after study. Palliative Medicine, 28(1), 34-41.

Temkin-Greener, H., Mukamel, D., Ladd, H., Ladwig, S., Caprio, T., Norton, S., Quill, T., Olsan, T., & Cai, X. (2018). Impact of Nursing Home Palliative Care Teams on End-of-Life Outcomes: a Randomized Controlled Trial. Medical care, 56(1), 11-18.

Thurston, J., & Waterworth, S. (2012). 'Making sense': nurses' experiences of changing practice in caring for dying patients in New Zealand. International Journal of Palliative Nursing, 18(10), 500-507.

Usher, B. M., DiNella, J., Ren, D., Liang, Z., & Tuite, P. K. (2015). Development of end-of-life peer nurse coaches: A hospital-based quality improvement project. Journal of Hospice and Palliative Nursing, 17(6), 551-558.

van der Plas, A. G., Pasman, H. R. W., Schweitzer, B., & Onwuteaka-Philipsen, B. D. (2018). Improving palliative care provision in primary care: a pre- and post-survey evaluation among PaTz groups. British Journal of General Practice, 68(670), e351-e359.

van der Plas, A. G., Vissers, K. C., Francke, A. L., Donker, G. A., Jansen, W. J., Deliens, L., & Onwuteaka-Philipsen, B. D. (2015). Involvement of a Case Manager in Palliative Care Reduces Hospitalisations at the End of Life in Cancer Patients; A Mortality Follow-Back Study in Primary Care. PLoS ONE, 10(7), e0133197.

van der Steen, J. T., Arcand, M., Toscani, F., de Graas, T., Finetti, S., Beaulieu, M., Brazil, K., Nakanishi, M., Nakashima, T., Knol, D. L., & Hertogh, C. M. (2012). A family booklet about comfort care in advanced dementia: three-country evaluation. Journal of the American Medical Directors Association, 13(4), 368-375.

Venkatasalu, M. R., Clarke, A., & Atkinson, J. (2015). 'Being a conduit' between hospital and home: Stakeholders' views and perceptions of a nurse‐led Palliative Care Discharge Facilitator Service in an acute hospital setting. Journal of Clinical Nursing, 24(11), 1676-1685.

Verhofstede, R., Smets, T., Cohen, J., Costantini, M., Noortgate, N., & Deliens, L. (2016). Implementing the care programme for the last days of life in an acute geriatric hospital ward: a phase 2 mixed method study. BMC Palliative Care, 15(1), 27.

Verreault, R., Arcand, M., Misson, L., Durand, P. J., Kroger, E., Aubin, M., . . . Carmichael, P.-H. (2018). Quasi-experimental evaluation of a multifaceted intervention to improve quality of end-of-life care and quality of dying for patients with advanced dementia in long-term care institutions. Palliative medicine, 32(3), 613-621.

Virani, R., Malloy, P., Dahlin, C., & Coyne, P. (2014). Creating a fabric for palliative care in safety net hospitals: End-of-life nursing education consortium for public hospitals. Journal of Hospice and Palliative Nursing, 16(5), 312-319.

Walczak, A., Butow, P., Tattersall, M., Davidson, P., Young, J., Epstein, R., Costa, D., & Clayton, J. (2017). Encouraging early discussion of life expectancy and end-of-life care: a randomised controlled trial of a nurse-led communication support program for patients and caregivers. International Journal of Nursing Studies, 67, 31-40.

Walker, K. A., Nachreiner, D., Patel, J., Mayo, R. L., & Kearney, C. D. (2011). Impact of standardized palliative care order set on end-of-life care in a community teaching hospital. Journal of Palliative Medicine, 14(3), 281-286.

Walker, R., & Read, S. (2010). The Liverpool Care Pathway in intensive care: an exploratory study of doctor and nurse perceptions. International Journal of Palliative Nursing, 16(6), 267-273.

Waller, A., Girgis, A., Johnson, C., Lecathelinais, C., Sibbritt, D., Forstner, D., Liauw, W., & Currow, D. C. (2012). Improving outcomes for people with progressive cancer: interrupted time series trial of a needs assessment intervention. Journal of Pain and Symptom Management, 43(3), 569-581.

Walling, A. M., Ettner, S. L., Barry, T., Yamamoto, M. C., & Wenger, N. S. (2011). Missed opportunities: Use of an end-of-life symptom management order protocol among inpatients dying expected deaths. Journal of Palliative Medicine, 14(4), 407-412.

Walling, A. M., Schreibeis-Baum, H., Pimstone, N., Asch, S. M., Robinson, L., Korlekar, S., Lorenz, K., Nwajuaku, T., & Rosenfeld, K. (2015). Proactive case finding to improve concurrently curative and palliative care in patients with end-stage liver disease. Journal of Palliative Medicine, 18(4), 378-381.

Weng, T. C., Yang, Y. C., Chen, P. J., Kuo, W. F., Wang, W. L., Ke, Y. T., Hsu, C. C., Lin, K. C., Huang, C. C., & Lin, H. J. (2017). Implementing a novel model for hospice and palliative care in the emergency department: An experience from a tertiary medical center in Taiwan. Medicine (United States), 96(19), e6943.

Wharton, T., Manu, E., & Vitale, C. A. (2015). Enhancing provider knowledge and patient screening for palliative care needs in chronic multimorbid patients receiving home-based primary care. American Journal of Hospice & Palliative Medicine, 32(1), 78-83.

Wickson-Griffiths, A., Kaasalainen, S., Brazil, K., McAiney, C., Crawshaw, D., Turner, M., & Kelley, M. L. (2015). Comfort Care Rounds: a staff capacity-building initiative in long-term care homes. Journal of Gerontological Nursing, 41(1), 42-48.

Williams, B. R., Woodby, L. L., Bailey, F. A., & Burgio, K. L. (2014). Formative evaluation of a multi-component, education-based intervention to improve processes of end-of-life care. Gerontology & Geriatrics Education, 35(1), 4-22.

Woo, J., Cheng, J. O. Y., Lee, J., Lo, R., Hui, E., Lum, C. M., Or, K. H., Yeung, F., Wong, F., & Mak, B. (2011). Evaluation of a Continuous Quality Improvement Initiative for End-of-Life Care for Older Noncancer Patients. Journal of the American Medical Directors Association, 12(2), 105-113.

Yamamoto, R., Kizawa, Y., Nakazawa, Y., Ohde, S., Tetsumi, S., & Miyashita, M. (2015). Outcome evaluation of the palliative care emphasis program on symptom management and assessment for continuous medical education: Nationwide physician education project for primary palliative care in Japan. Journal of Palliative Medicine, 18(1), 45-49.

Yoshioka, S., Moriyama, M., & Ohno, Y. (2014). Efficacy of the End-of-Life Nursing Care Continuing Education Program for Nurses in General Wards in Japan. American Journal of Hospice and Palliative Medicine, 31(5), 513-520.
